# A Novel Liquid Chromatographic Time-of-Flight Tandem Mass Spectrometric Method for the Determination of Secondary Metabolites in Functional Flours Produced from Grape Seed and Olive Stone Waste

**DOI:** 10.3390/molecules30071527

**Published:** 2025-03-29

**Authors:** Achilleas Panagiotis Zalidis, Natasa P. Kalogiouri, Ioannis Mourtzinos, Dimitris Sarris, Konstantinos Gkatzionis

**Affiliations:** 1 Laboratory of Consumer and Sensory Perception of Food & Drinks, Department of Food Science and Nutrition, University of the Aegean, Metropolite Ioakeim 2, 81400 Myrina, Greece; achilles.zalidis@gmail.com (A.P.Z.); dsarris@aegean.gr (D.S.); 2 Laboratory of Analytical Chemistry, School of Chemistry, Aristotle University of Thessaloniki, 54124 Thessaloniki, Greece; 3 Laboratory of Food Chemistry and Biochemistry, Department of Food Science and Technology, School of Agriculture, Aristotle University of Thessaloniki, 54124 Thessaloniki, Greece; mourtzinos@agro.auth.gr

**Keywords:** phenolics, liquid chromatography, functional flours, olive by-products, pulse flours

## Abstract

Agricultural by-products like grape pomace and olive stones are rich in bioactive compounds and can be processed into grape seed and olive stone flours.The phenolic composition of such flours still remains underexplored. This study introduces a liquid chromatographic time-of-flight tandem mass spectrometric method (LC-QTOF-MS/MS) to assess the phenolic profiles of functional flours from different origins and evaluate their potential use within the frame of a circular economy. Grape seed and olive stone flours from Lemnos and commercial sources were analyzed employing target, suspect, and non-target screening. Target screening resulted in the determination of 23 phenolic compounds. Suspect screening revealed phenolic diversity in flours produced in Lemnos island. Non-target screening resulted in the detection of 1042 and 1620 mass features in grape seed and olive stone flours, respectively. Principal component analysis (PCA) and partial least squares-discriminant analysis (PLS-DA) successfully differentiated samples between commercially available and those produced in Lemnos. These results underscore the phenolic richness of grape seed and olive stone flours, supporting their use as functional ingredients and reinforcing sustainability and circular economy principles in the agri-food sector.

## 1. Introduction

The concept of circular economy has gained substantial traction in recent years as a sustainable framework aiming to minimize waste generation and promote resource efficiency. Central to this paradigm is the valorization of waste streams, transforming them into valuable resources through innovative approaches [1]. In the agricultural sector, where considerable quantities of by-products are generated, there exists significant potential for circular economy practices.

Among the diverse agricultural waste streams, the olive oil and wine industry by-products, predominant in Mediterranean regions, constitute a significant portion of agricultural waste [2]. These by-products are rich in bioactive compounds, dietary fibers, and other valuable components, rendering them suitable candidates for fortifying food and enriching its health-promoting properties [3,4]. By-products from the aforementioned streams include grape pomace, which could be milled into flour-producing grape seed flour, as well as olive stones, resulting in a fine olive stone powder/flour. Polyphenols in grapes are mainly reported in the seeds, in the range between 60% and 70% of the total extractable polyphenols [5]. Olive stones are a valuable source of micronutrients as well. Grinding olive stone into a powder and incorporating it into flour can enhance the nutritional content of dough products and snacks, enriching it with dietary fibers and phenolic compounds [6]. High concentrations of oleoside, nuezhenide, oleuropein verbascoside, ligstroside, and glycosides of tyrosol and hydroxytyrosol [7] with positive health effects have been reported in the literature [8,9]. A recent study [10] has illustrated the antioxidant and anti-inflammatory properties of the biomolecules in grape by-products and wastes as well as the cardiovascular protection and diabetes management they provide. Among the active metabolites that play a very important role are phenols, tannins, resveratrol, quercetin, flavonoids, and anthocyanins [11]. Additionally, grape seeds are rich in catechin and gallic acid [12], while the presence of epicatechin gallate and gallates of dimeric and trimeric procyanidins has also been reported [13].

The valorization of olive stones and grape seeds aligns with the principles of the circular economy by converting these agricultural wastes into valuable resources with diverse applications [14]. By incorporating them into food products and nutraceuticals, these by-products could contribute to reducing waste generation, promoting sustainable practices across various industries and further enriching the available nutrients. The current state of the art indicates that the primary use of olive stone flour is centered on creating polypropylene composites [15], with few applications in food, mainly in dough products [6,16]. Considering grape seed flour, the majority of the available studies focus on the effects of wheat flour substitution and the rheological parameters of doughs [17,18]. Additionally, the nutritional profile of food products has been investigated [19] along with the antioxidant activity and consumer acceptance [20,21].

The environmental conditions under which olive trees and grapevines grow significantly impact the composition of their by-products and, consequently, the properties of the functional flours derived from them. Olive trees are typically cultivated in dry, arid, and nutrient-poor soils, requiring minimal water input and exhibiting resilience to harsh climatic conditions [22]. These factors influence the chemical composition of olive stones, often resulting in a higher concentration of specific phenolic compounds such as oleuropein and hydroxytyrosol [23]. In contrast, grapevines thrive in more temperate and humid environments with richer soil conditions that promote the accumulation of diverse polyphenolic compounds, including flavonoids and tannins [24]. Additionally, the processing methods for olives and grapes differ considerably; olive processing involves mechanical extraction and prolonged exposure to water [25], while winemaking subjects grape seeds to fermentation processes that can alter their polyphenolic profile [26]. These environmental and processing differences contribute to distinct nutritional and bioactive properties in olive stone and grape seed flours, influencing their potential applications in food and nutraceutical products.

Even though numerous works are focusing on the analysis of olive and grape by-products as a valuable alternative source of bioactive compounds, there are limited studies evaluating the phenolic profile of the flour that is produced from grape seeds and olive stones. The analysis of grape seed and olive stone functional flours is vital for unlocking their full potential as an antioxidant and health-promoting sustainable ingredient in food production. Even though there are numerous works proposing analytical methods for food by-products, the literature regarding the in-depth characterization of functional flours is still scarce. High-resolution mass spectrometry plays a key role in food fingerprinting studies. Liquid chromatography coupled with time-of-flight tandem mass spectrometry (LC-QTOF-MS/MS) enables the detailed characterization of bioactive compounds, such as polyphenols and other secondary metabolites, with high resolution owing to its ability to differentiate among compounds with same unit mass but differing in mass defects. This is particularly useful, as LC-QTOF-MS/MS allows the comprehensive profiling of both known and unknown compounds, through target, suspect, and non-target screening.

Despite the promising potential of small molecules as biomarkers for assessing the quality and origin of olive stone and grape seed flours, their variability due to environmental factors poses a challenge to their reliability. Climatic conditions, soil composition, and agricultural practices can significantly alter the metabolite profile, potentially impacting their consistency as origin markers and affect data interpretation [27]. In this study, the phenolic fingerprint of grape seed and olive stone flours from Lemnos island and commercial sources was investigated. Target, suspect, and non-target screening were employed to assess their phenolic profile, evaluate their potential as raw materials in technological applications, and investigate if these small molecules could be used as markers in origin authenticity studies to distinguish between commercially available functional flours and those originating from Lemnos island. Future studies should aim to conduct multi-location studies to better assess the robustness of small molecules as authenticity markers.

## 2. Results and Discussion

### 2.1. Grape Seed Flour (GSF)

#### 2.1.1. Target Screening Results

By scanning the flour samples and referring to the initial target list (Table S1, Supplementary Material), the presence of 23 target compounds was identified. These compounds included flavonoids: apigenin, chrysin, diosmin, luteolin, kaempferol, myricetin, quercetin, taxifolin (dihydroquercetin), naringenin, catechin, epicatechin, epigallocatechin, epicatechin gallate, rutin (quercetin-3-*O*-rutinoside), quercitrin (quercetin-3-*O*-rhamnoside), and myricitrin (myricetin-3-*O*-rhamnoside); phenolic acids: caffeic acid, coumaric acid, ferulic acid, sinapic acid, protocatechuic acid, gallic acid, and vanillic acid; and phenolic aldehyde (vanillin). Their presence was confirmed by comparing the experimental molecular ions and retention times with the corresponding standards, using a maximum threshold of ΔRt = 0.2 min. The most abundant fragments from the MS/MS spectra were recorded along with their elemental formulas, and the compounds were quantified based on peak areas. The target screening results for GSF are summarized in Table S2 along with the concentration (mean) for each compound. The extracted ion chromatograms (EICs), MS, and MS/MS spectra for GSF are shown in Figures S1–S14 in the Supplementary Materials.

**Figure 1:**
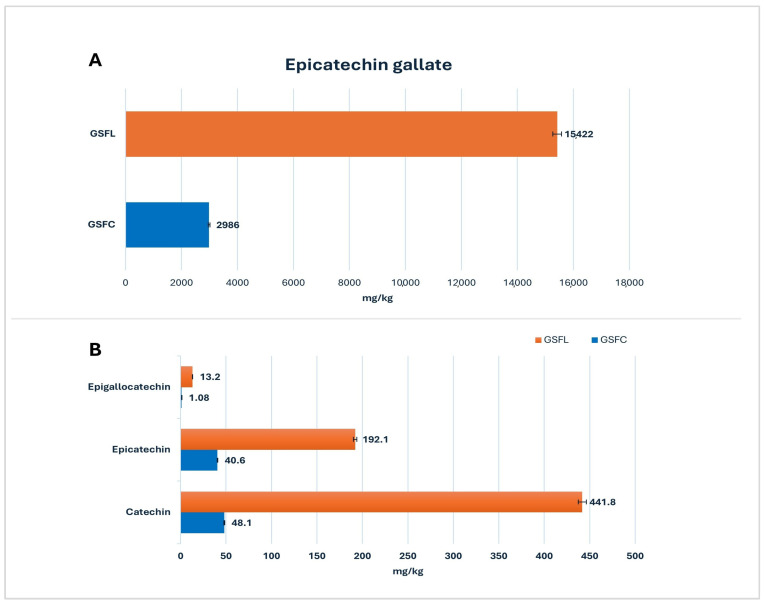
(**A**) Mean concentration (mg/kg) of epicatechin gallate in commercial grape seed flour (GSFC: *n* = 10) and grape seed flour from Lemnos (GSFL: *n* = 10). (**B**) Mean concentration (mg/kg) of major phenolic constituents in commercial grape seed flour (GSFC: *n* = 10) and grape seed flour from Lemnos (GSFL: *n* = 10).

Grape seeds offer several advantages for human consumption due to their phenolic content and other substances [28]. Catechin, epicatechin, gallic acid, gallocatechin, and epicatechin gallate are major phenolic constituents of grape seeds and could make up to 45% of the total phenolic content [29,30]. In Figure 1A, the mean concentration of epicatechin gallate is shown, which was the most abundant phenolic compound in grape seed flour from Lemnos (GSFL) and five times higher than that in commercial grape seed flour (GSFC). In Figure 1B, catechin, epicatechin, and epigallocatechin, the aforementioned compounds, are shown. GSFL again exhibited a higher mean concentration in the detected compounds with significant differences when compared to GSFC. The detected catechins have been previously associated with cardioprotective properties [31]. In a skin cancer cell line model, a grape seed extract rich in catechins significantly reduced apoptosis, lipid peroxide levels, lesion scores, and DNA damage [32].

In addition, GSFL yielded a higher concentration in the majority of the target substances with the exception of: kaemferol (GSFC: 3.22 mg/kg (sd = 0.31) and GSFL: 2.20 mg/kg (sd = 0.08)), myricitrin (GSFC: 4.85 mg/kg (sd = 0.10) and GSFL: 4.51 mg/kg (sd = 0.06)), and quercitrin (GSFC: 5.72 mg/kg (sd = 1.087) and GSFL: 3.88 mg/kg (sd = 0.18)).

#### 2.1.2. Suspect Screening Results

GSF samples were further screened using the suspect list (Table S3, Supplementary Material), which was created from the literature. The presence of the compounds was tentatively verified on the basis of the accurate mass and examining the MS/MS spectra using in silico fragmentation tolls, such as MetFrag [33] and MassBank [34], and literature records. The suspect compounds were tentatively semi-quantified using the calibration curves of same-class compound derivatives. In total, 34 compounds were identified in GSF, and the suspect screening results from commercial grape seed flour (GSFC) and grape seed flour from Lemnos (GSFL) are shown in Table S4. The identified compounds belong to various phytochemical classes, including phenolic acids, flavonoids, anthocyanins, coumarins, and stilbenes. The phenolic acids include hydroxycinnamic acid derivatives such as 3-caffeoylshikimic acid, caftaric acid, chicoric acid, coutaric acid, and fertaric acid, as well as hydroxybenzoic acids like ellagic acid, ellagic acid hexoside 1, and p-hydroxybenzoic acid. The flavonoids are well represented, encompassing flavanols, flavonols, flavones, and their glycosides. Notable flavonoids include dihydrokaempferol-3-*O*-rhamnoside, epicatechin-3-*O*-gallate/trimer, epigallocatechin gallate, eriodyctiol-7-*O*-glucoside, gallocatechin, isorhamnetin-3-*O*-glucoside, isorhamnetin-3-*O*-rutinoside, kaempferol-3-*O*-glucoside, laricitrin-3-*O*-glucoside, luteolin-7-*O*-glucoside, myricetin-3-glucoside, myricetin-3-*O*-glucuronide, procyanidin A1, procyanidin B1/B2, quercetin-3-*O*-galactoside, quercetin-3-*O*-glucoside, quercetin-3-*O*-glucuronide, quercetin-3-*O*-rhamnoside, quercetin-3-*O*-rutinoside (rutin), taxifolin-3-*O*-glucoside, taxifolin-3-*O*-rhamnoside, and trifolin. The anthocyanin cyanidin-3-*O*-glucoside was also identified. Additionally, the coumarin fraxin was detected, along with stilbenes such as trans-piceatannol, trans-piceid (trans-polydatin), and trans-resveratrol.

Grape seeds have been previously confirmed as a rich source of phenolic compounds, including procyanidins [35], cinnamic acid derivatives [36], and flavonoids [29], all of them linked with numerous health benefits. Procyanidin A1 and B1 were identified in both GSFC and GSFL, with higher concentrations in GSFL. Specifically, procyanidin A1 was 4.5 times higher in GSFL compared to GSFC, while procyanidin B1/B2 was 1.5 times higher in GSFL. Proanthocyanidins in grape seeds have been previously associated with cholesterol metabolism, and their incorporation in animal feed serves as an effective dietary supplement improving lipid composition [37]. Additionally, recent evidence showed promise as nutraceuticals since they affect microbial ecology and gut microbiota [38].

Ellagic acid and its derivative (ellagic acid hexoside) were abundant in GSFC, and evidence indicates that the intake of ellagic acid is effective in reducing obesity and improving obesity-related metabolic complications [39]. Coutaric acid was significantly higher in GSFL (17.6 mg/kg (sd = 0.005) compared to 87.7 mg/kg (sd = 0.02)) and caftaric acid was not detected in GSFC (GSFL: 10.7 mg/kg). Significant flavonoids found in grape skin and seeds, such as gallocatechin, epigallocatechin gallate, and epicatechin-3-*O*-gallate, were notably higher in GSFL. Overall, GSFL exhibited higher concentrations of major phenolic constituents.

This detailed identification of bioactive compounds in grape seed products not only highlights their potential health benefits but also provides a foundation for waste stream valorization. The sustainable management of food waste, such as grape seeds, involves converting these by-products into valuable resources. While utilizing the bioactives from these by-products, a circular economy is supported by reintegrating these compounds into the food chain, reducing environmental impact, and enhancing the nutritional value of food products [40]. Furthermore, these identified compounds could potentially act as markers for authenticity and origin determination, offering a valid method to distinguish among those from different sources. This approach transforms what was once considered waste into a resource that aligns with sustainability goals, contributes to waste reduction, and fosters transparency in food sourcing and product labeling.

### 2.2. Olive Stone Flour (OSF)

#### 2.2.1. Target Screening Results

The target screening process for OSF was the same that was used for GSF, and the target screening results with the respective mean concentration are summarized in Table S5. The extracted ion chromatograms (EICs), MS, and MS/MS spectra for OSF are shown in Figures S15–S29 in the Supplementary Materials.

A lower number of phenolic compounds was detected in OSF compared to GSF. Protocatechuic acid, vanillin, and luteolin were the most abundant compounds, at 95.7 mg/kg (sd = 0.98), 89.8 mg/kg (sd = 1.07), and 28.3 mg/kg (sd = 0.66) respectively. The Lemnos-variety olive stone flour (OSFL) exhibited an increased concentration in most substances, with quercitrin, a valuable curative agent [35], having been detected in abundance. Moreover, diosmin and taxifolin were unique to OSFL and not detected in commercial olive stone flour (OSFC). Diosmin has been found to be effective in reducing the proliferation of colon cancer cell metabolism [41], while taxifolin has demonstrated biological activities, such as anti-Alzheimer activity, anti-microbial activity, hepatoprotective activity, among others [42]. These compounds are already utilized as dietary supplements and in novel food products [43], while there are studies that support the addition of olive stone powder in biscuits in order to boost their nutritional properties [16]. However, the research did not include a detailed characterization of the phenolic content using chromatographic techniques. This omission means that, while the overall phenolic content increased, the specific phenolic compounds present were not identified or quantified, limiting the deeper understanding of the bioactive properties of olive stone powder. Incorporating chromatography could offer more precise insights into these bioactives, such as diosmin and taxifolin, and take advantage of them being unique to GSFL.

#### 2.2.2. Suspect Screening Results

OSF samples were further screened using the suspect list (Table S6, Supplementary Materials). The tentative verification followed the GSF suspect screening protocol. In total, 63 compounds were present in OSF, and the suspect screening results for OSFC and OSFL are shown in Table S7. The compounds identified in OSF flours include a variety of phytochemical classes. Phenolic acids, such as 4-hydroxybenzoic acid, benzoic acid, chlorogenic acid, gentisic acid, and homovanillic acid, were present, along with flavonoids, including apigenin derivatives, chrysoeriol-*O*-glucoside, luteolin derivatives, kaempferol derivatives, quercetin derivatives, eriodictyol, naringin, gallocatechin, genistein, and 2′-hydroxygenistein. Secoiridoids, such as decarboxymethyl oleuropein aglycone (oleacein), oleuropein aglycone, oleoside, and elenolic acid, were also detected. Additionally, lignans, like hydroxypinoresinol, olivil, pinoresinol, and syringaresinol, as well as coumarins such as esculetin and fraxamoside, were identified. Stilbenes, like trans-polydatin, and triterpenoids, such as maslinic acid and oleanolic acid, were also found. Other phenolic compounds included tyrosol, tyrosol glucoside (salidroside), and verbascoside.

Olive stone contains a wide variety of phenolic compounds, including polyphenols [44], prenol lipids, secoiridoids [45], and flavonoids [46], which are potent antioxidants and linked to positive effects on human health by a large number of studies [28]. Tyrosol and hydroxytyrosol, two of the main compounds of the phenolic fraction of olive stones [47], were significantly higher in OSFL compared to OSFC. Moreover, oleuropein and its derivative (oleuropein aglycone), which are considered the most prevalent polyphenols in olives [48], were abundant in GSFC. The homovanillic acid concentration was six times higher in GSFC, with similar results for the 4-hydroxybenzoic acid, both major constituents in olives [49]. Concerning lignans in olive stone, olivil and pinoresinol were both identified and exhibited higher concentrations in OSFC. Verbascoside, which has been reported in olive mill wastewater and is considered a possible food antioxidant [50], was notably higher in commercial grape seed flour. In addition, oleanolic acid, a triterpenic acid with therapeutic potential [51], was detected in a high concentration in OSFC.

Given the importance of phenolic compounds in OSF, it is crucial to characterize them before they are incorporated into functional food products. These compounds could serve as unique chemical markers, reflecting specific environmental conditions tied to the geographical origin, thus enabling the distinction between flours from Lemnos and their commercial counterparts. Furthermore, in order to address the presence of soluble and hydrolyzable phenolic compounds in olive stone, studies highlight the importance of these compounds for their antioxidant potential [52]. Soluble phenols in olive stone can be criticized for their instability during food processing, which can reduce their functional efficacy. By deploying HRMS methods, it is possible to identify and monitor the turn-over of the specific classes of polyphenols, offering more precise insights into their composition and allowing for better utilization in functional food products.

### 2.3. Non-Target Screening Results

**Figure 2:**
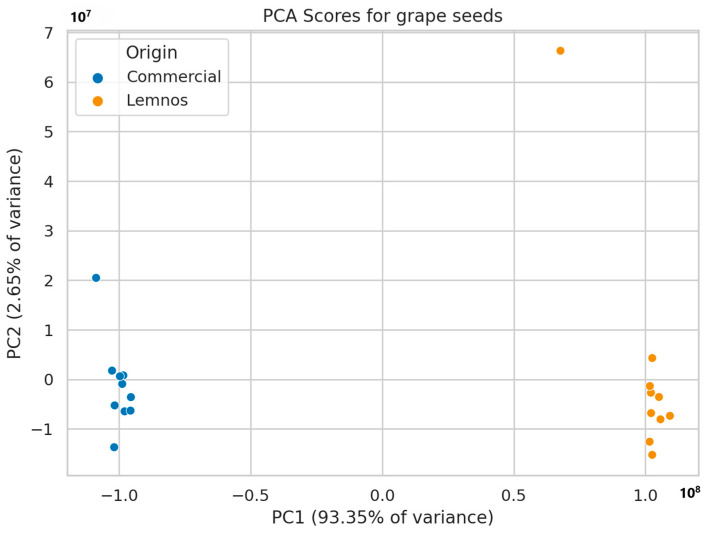
PCA model applied to GSFC (blue) and GSFL (orange).

**Figure 3:**
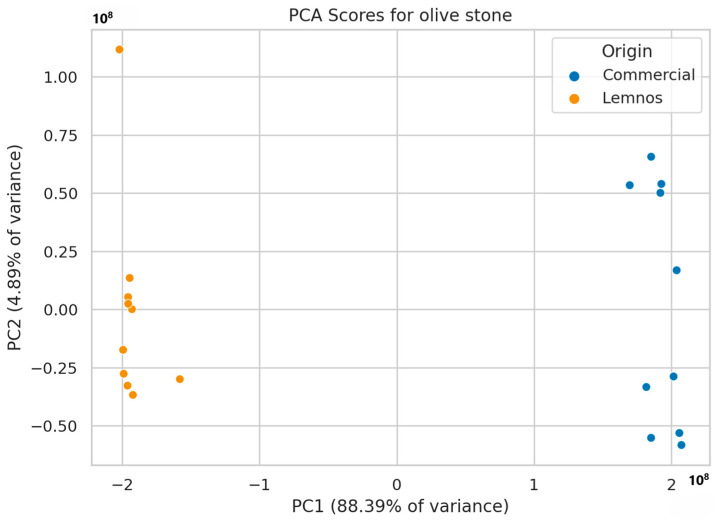
PCA model applied to OSFC (blue) and OSFL (orange).

Non-target screening resulted in the generation of 1042 mass features in total for grape seed flour (GSFC and GSFL) and 1620 for olive stone flour (OSFC and OSFL). A principal component analysis (PCA) was utilized for the initial assessment of sample distribution. In GSF, the first two principal components explained 96.0% of the variation and two distinct groups were formed, namely commercial and Lemnos grape seed flours, as seen in Figure 2 (see also Supplementary Figure S30 for the loadings plot), while similar results were obtained for OSF (Figure 3), with PC1 and PC2 explaining 93.3% of the variation (see also Supplementary Figure S31 for the loadings plot).

**Figure 4:**
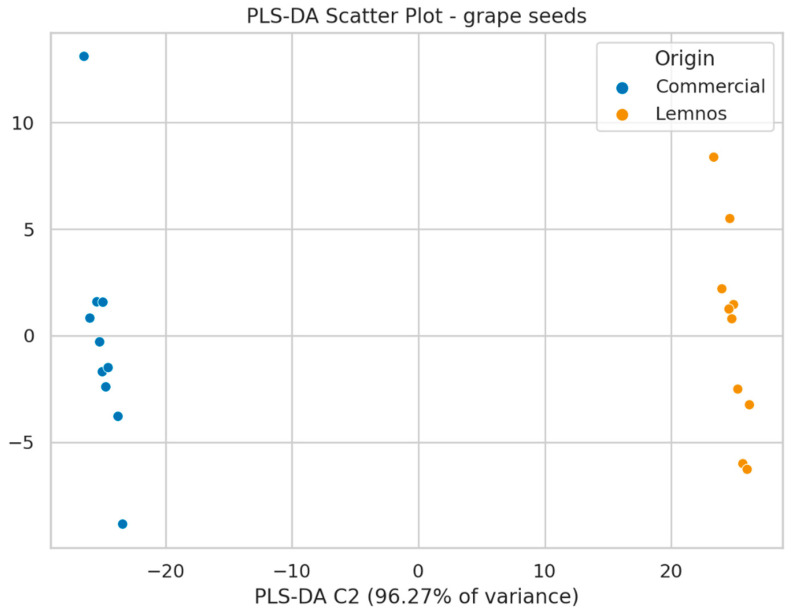
PLS-DA model applied to GSFC (blue) and GSFL (orange).

**Figure 5:**
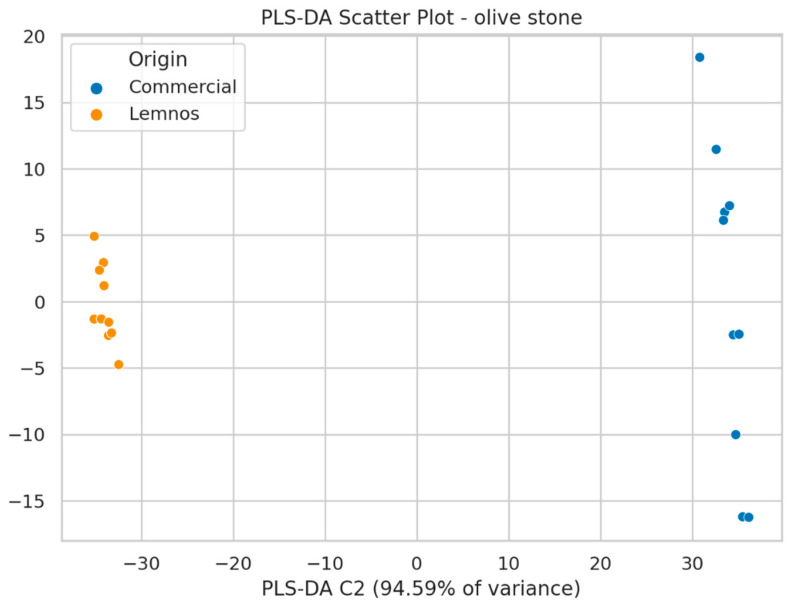
PLS-DA model applied to OSFC (blue) and OSFL (orange).

The next step involved the use of partial least squares-discriminant analysis (PLS-DA), which successfully distinguished commercial flours and the flour from Lemnos, both for grape seeds and olive stones (Figure 4 and Figure 5; see also Supplementary Figures S32 and S33 for the loadings plots). VIP scores were calculated for the PLS-DA model to identify the most significant features responsible for the classification of the samples. Compounds with VIP scores above 1.0 were selected as the most important, and in total, 56 mass features had high values for GSF, indicating they significantly contributed to the sample clustering, while 99 mass features contributed to the clustering for OSF (Figures S34 and S35). The model’s predictive value was satisfactory, with the goodness of fit (R2Y = 0.972) and predictability (Q2 = 0.796) values being acceptable. Permutation test statistics with 100 random permutations confirmed the model’s validity, as all permuted R2 and Q2 values were lower than the original values [53] (Figures S36 and S37). A receiver operating characteristic (ROC) curve was constructed to evaluate the model’s performance by plotting true positive and false positive rates. The area under the ROC curve (AUC) indicated that the model could classify all samples (GSF and OSF samples as well) with 100% accuracy (Figures S38 and S39).

The aforementioned VIP mass features for GSF and OSF are shown in Table 1. All features were tentatively identified using the SCIEX Natural Products Library, with a Library Match Score above 50.0. The MS/MS fragments were compared using literature records [54,55,56,57].

In OSF samples, 4-dihydroxybenzoic acid is a phenolic compound, related to other phenolics found in olives like hydroxytyrosol and oleuropein. Elenaic acid and one derivative of elenaic acid were tentatively identified. 16-Hydroxy-hexadecanoic acid, 9,10,18-trihydroxyoctadecanoic acid, (9R,10S)-dihydroxystearate, 13-hydroperoxylinolenic acid, and hexadecanedioate are fatty acid derivatives that are present in olives and, therefore, it is concluded that the functional ingredients of the matrix are present in the flour samples as well. Furthermore, 18-hydroxyoleate is a derivative of oleic acid, the primary fatty acid in olives. Chrysoeriol is a flavonoid that can be found in olive leaves and possibly in small amounts in olive oil. An intermediate in fatty acid oxidation. Salidroside, a glucoside derivative of tyrosol, is also found in olives as a marker and has been revealed to improve memory retrieval [58]. Cornoside is a lingstroside found in olives that is relatively abundant in young small olives [59]. All compounds are either directly found in olives or are related to compounds present in olives. Their nutritional significance lies primarily in their roles as antioxidants, anti-inflammatory agents, and contributors to lipid metabolism. The non-target screening results of the OSF samples are summarized in Table 1.

**Table 1:** Non-target screening results for olive stone flour (OSF) samples, including mass features with a variable importance in projection (VIP) score above 1.0, along with their library matches and plausible molecular formulas, tentative analytes, and Formula Finder scores.

[M − H]^−^/RT	VIP	Plausible Molecular Formula	Tentative Analyte	Phytochemical Class	Formula Finder Score	MassBank ID/Reference
109.0292/3.32	1.70990591	C_6_H_5_O_2_			99	
123.0449/3.61	3.88331912	C_7_H_8_O_2_			99.9	
153.0191/3.32	1.24806817	C_7_H_6_O_4_	2,4-dihydroxybenzoic acid	Phenolic acid	91.2	BS003106
153.0554/3.61	4.87762151	C_8_H_10_O_3_			94.6	
171.1023/7.77	1.32240845	C_8_H_14_O			90	
187.0966/7.65	2.20468526	C_9_H_16_O_4_			82.76	
199.0611/4.37	1.10983312	C_8_H_10_O_3_	Halleridone	Terpenoid	90.7	
215.0556/4.16	2.44049438	C_9_H_12_O_6_			90.8	
215.0914/6.36	1.91315487	C_11_H_20_S_2_			90.4	
223.0612/5.43	2.02782415	C_12_H_16_S_2_			89	
229.0717/4.57	1.35870769	C_10_H_14_O_6_	Elenaic acid	Lipid	85.1	[60]
241.1186/5.76	2.91752206	C_11_H_14_O_6_	Elenaic acid derivative	Lipid	84.1	
253.2153/13.65	1.68171724	C_16_H_32_O_3_	16-Hydroxy-hexadecanoic acid	Lipid	91.1	PR100487
265.0753/7.30	1.11639129	C_10_H_18_O_6_S			90.5	
265.1462/13.56	5.82308316	C_12_H_26_O_4_S			90.9	
266.1507/13.64	1.01144158	C_6_H_21_N_9_OS			91.2	
267.1956/12.45	1.32783509	C_16_H_30_O_4_	Hexadecanedioate	Lipid derivative	86.3	JP001078
269.0445/9.02	2.64017779	Apigenin (C_15_H_10_O_6_)			98.9	
275.1039/6.15	1.30711949					
279.2311/13.76	1.75842018	C_14_H_30_N_6_O			99.6	
281.2468/13.99	3.41644995	C_18_H_36_O_3_			77.7	
282.2510/13.99	3.53055616					
283.2614/14.36	2.88130084	C_14_H_32_N_6_			90.9	
285.0386/8.36	9.07714971	C_15_H_10_O_6_	Luteolin	Flavone	95.9	PM000420
286.0441/8.50	1.61411498					
287.2212/10.64	5.0420702	C_16_H_32_O_4_			90.6	
293.2108/12.78	4.11240994					
294.2158/12.84	1.05069407	C_18_H_34_O_3_				
295.2255/12.81	2.38947953	C_16_H_12_O_6_				
297.2417/13.64	1.15555122		18-Hydroxyoleate	Lipid	97	[61]
299.0558/9.11	1.06125804	C_16_H_30_O_5_	Chrysoeriol	Flavone	92.1	BS003342
299.1136/4.50	2.42334052	C_5_H_10_N_10_O_6_				
301.2009/10.45	2.5807432	C_18_H_30_O_4_			92.3	
305.0701/5.34	1.59445723	C_18_H_34_O_5_			94.3	
309.2069/12.08	1.20749047		13(S)-Hydroperoxylinolenic acid	Lipid derivative	91.9	EQ331602
311.2220/12.14	1.00496969	C_14_H_20_O_8_			90.2	
313.2372/12.31	2.63234722	C_18_H_36_O_4_				
315.1086/3.82	1.87543365	C_12_H_31_N_9_O	Cornoside	Monoterpenoid	82.2	[62]
315.2516/12.68	5.75432159	C_18_H_32_O_5_	(9R,10S)-Dihydroxystearate	Lipid	83.8	
316.2560/12.68	1.87253721	C_18_H_34_O_6_			99.6	
327.2165/9.86	1.8513419	C_18_H_34_O_5_			74.5	
327.2170/10.58	1.27970038	C_18_H_34_O_5_			89.4	
329.2309/9.90	8.7666785	C_18_H_36_O_5_			84.9	
329.2312/10.46	15.8827924	C_18_H_36_O_5_			85	
331.2397/10.58	3.15270061	C_20_H_18_O_5_			73.7	
331.2471/10.32	4.29045396	C_15_H_24_N_10_	9,10,18-Trihydroxyoctadecanoic acid	Lipid	82.9	CB000003
337.1071/7.59	1.13852175	C_14_H_20_O_7_			98.7	
343.2103/9.76	5.61777449	C_18_H_34_O_6_			90.1	
345.1194/4.45	1.59752959	C_21_H_38_O_5_	Salidroside	Phenol	93.2	[63]
345.2267/10.14	2.43118178	C_14_H_30_O_4_S			94.7	
351.2535/13.80	1.65705829				90.7	
353.1979/14.39	1.32301547	C_29_H_14_N_2_			90.1	
353.2683/14.03	3.8098374	C_24_H_40_O_4_				
389.1082/5.26	2.22477443	C_30_H_16_N_2_			98.7	
391.2846/13.98	1.54222771	C_30_H_16_N_2_			99.9	
403.1231/5.43	11.927081	C_10_H_24_N_6_O_9_S			97.7	
403.1241/6.21	1.51585553	C_27_H_19_NO_3_			93	
403.1248/4.90	1.70275032	C_22_H_22_N_2_O_6_			91.9	
404.1284/5.46	2.71837775	C_26_H_42_O_4_			83.2	
409.1404/9.40	1.02744581	C_25_H_36_O_6_			94.9	
417.2995/14.02	2.96165422	C_25_H_36_O_7_			86	
431.2417/12.15	4.06742772	C_30_H_48_O_3_			98.4	
447.2371/12.05	1.69705193				79.9	
455.3500/13.59	7.58565491	C_30_H_48_O_4_			89.5	
465.3191/13.91	2.81214421	C_21_H_46_N_8_O_4_				
471.3445/13.17	7.15603201	C_30_H_46_O_5_			88.3	
473.3526/13.15	1.17624378	C_26_H_44_N_6_O_3_			91.2	
485.3251/12.40	1.38424034	C_22_H_24_N_10_O_6_			94.9	
487.3408/12.62	1.05541295	C_26_H_28_N_4_O_8_			89.4	
523.1812/7.95	2.2817832	C_22_H_24_N_10_O_7_			92.5	
523.1823/6.69	1.98662549	C_31_H_28_N_4_S_3_			94.9	
539.1758/7.35	4.19041331	C_30_H_62_N_6_O_4_			93.2	
551.1401/7.20	1.30540864	C_27_H_30_N_4_O_9_			89.5	
551.4651/14.77	1.80521386	C_27_H_34_N_4_O_9_			94.5	
553.1923/8.17	1.01121826	C_27_H_26_N_4_O_10_			80.5	
557.2215/9.09	1.91276674	C_34_H_64_O_6_			92.1	
565.1570/7.80	1.48905009	C_37_H_72_S_2_			95.5	
567.4602/14.38	1.17748434	C_32_H_46_N_4_O_2_S_2_			91.2	
579.4967/15.89	1.53743162	C_36_H_65_O_6_			94	
581.2984/15.37	1.15630577	C_38_H_46_O_4_S			94.9	
593.4753/14.42	2.86762481	C_30_H_42_N_6_O_7_			87.7	
597.3028/14.89	1.26409343	C_26_H_28_N_10_O_9_			91.7	
597.3045/15.81	2.16408575	C_27_H_50_N_8_O_9_			90.4	
623.1956/6.31	6.10963709	C_36_H_38_N_4_O_8_S			93.1	
629.3615/3.80	1.63848932	C_36_H_38_N_4_O_8_S			87.2	
685.2318/6.70	11.0557952	C_37_H_34_N_8_O_4_S			94.3	
685.2332/8.13	4.26228369	C_36_H_38_N_4_O_8_S			94.8	
685.2340/7.49	2.87707627	C_34_H_44_N_2_O_9_S_2_			94.4	
685.2350/8.85	2.42497379	C_29_H_46_N_6_O_6_S_4_			95.6	
687.2405/6.71	2.44188861	C_37_H_40_N_4_O_9_S			94.7	
701.2282/6.27	3.26078709	C_39_H_48_O_10_S_3_			95.7	
715.2448/6.86	2.14610112	C_33_H_42_N_9_O_7_S_3_			95.2	
771.2324/7.56	6.88415023	C_39_H_44_N_4_O_7_S_3_			91.3	
772.2375/7.58	3.06952732	C_40_H_46_N_4_O_7_S_3_			90.7	
775.2283/7.13	5.04216623	C_32_H_42_N_10_O_10_S_2_			97.6	
789.2423/6.92	5.89403491	C_38_H_74_N_8_O_10_S			94.3	
789.2428/7.76	6.82049513	C_43_H_80_N_8_S_4_			94.8	
833.5162/13.97	1.10683778	C_6_H_5_O_2_			99	
835.5312/13.98	2.88542004	C_7_H_8_O_2_			96	

In GSF samples, non-target screening resulted in the tentative identification of 12 compounds, which are listed in Table 2. Tartaric acid is a major organic acid in grapes, contributing to the acidity of grape products. Gluconic acid is another compound found in grapes. Catechin and quercetin were already determined via target screening. Brevifolincarboxylic acid is a phenolic compound with potential health effects. Ellagic acid is present in grapes. Phloionolic acid is a triterpenoid compounds that can be found in certain grape species. Amurensisin is also found in grape species. Epicatechin gallate is a flavonoid known for its antioxidant activity. Oleanolic acid is a triterpenoid found in grape skins, though it is more abundant in other plants. Myricetin-3-*O*-glucoside is a myricetin derivative common in grape skins that contributes to the antioxidant properties. Persicogenin was also detected. Quercetin 3-*O*-glucuronide (miquelianin) is a quercetin derivative found in grape skins. These compounds can potentially be used as markers for the discrimination of flours on the basis of their geographical origin.

In conclusion, non-target analysis facilitated the creation of a robust PLS-DA model that accurately classified the samples according to their origin. The further application of chemometric models to GSF and OSF revealed distinct bioactive fingerprints among functional flours from different origins. This variation is likely due to the samples originating from different geographical regions, agricultural practices, and milling processes.

## 3. Materials and Methods

### 3.1. Flour Samples

Twenty flours originating from Lemnos were sourced locally, namely ten grape seed flours (GSFLs) and ten olive stone flours (OSFLs). Concerning the grape seed flours, a mass of grapes was provided by local distilleries, from which the seeds were separated and dried for 24 h. Then, they were ground in a professional mixer, and the resulting powder was passed through a sieve to obtain the smallest grain-size flour. Olive stone flour was prepared similarly to grape seed flour. A mass of olive skins and stones was supplied from local farmers, was dried for 24 h, and then ground into a fine powder, which was sieved. Additionally, ten commercially available grape seed flours (GSFCs) and ten olive stone flours (OSFCs) were procured from two separate retailers. Specifically, GSFC was sourced from PaleoCentrum in Budapest, Hungary, and OSFC was purchased from Nutexa in Valencia, Spain.

### 3.2. Chemicals and Standards

Methanol and water (LC-MS grade) were purchased from HiPerSolv CHROMANORM, VWR Chemicals BDH (Amsterdam, The Netherlands). Formic acid 98–100% was purchased from Merck (Darmstadt, Germany). For the determination of phenolic compounds, apigenin 98%, caffeic acid 98%, catechin 97%, cinnamiccinnamic acid 97%, chrysin 98%, diosmin 97%, epicatechin, epigallocatechin 97%, ferulic acid 98%, epicatechin gallate 98%, hesperidin 98% (internal standard), kaempferol 98%, luteolin 98%, myritecin 97%, myricitrin 97%, naringin 98%, p-coumaric acid 98%, quercetin 98%, quercitrin 99%, rosmarinic acid 98%, protocatechuic acid 97%, rutin 98%, sinapic acid 98%, syringaldehyde 97%, syringic acid 98%, taxifolin 98%, vanillic acid 98%, and vanillin 98% were used and were purchased from Sigma-Aldrich (Stenheim, Germany). Stock standard solutions of all the analytical standard compounds were prepared with LC-MS-grade methanol at 1000 mg/L and were afterward stored in dark brown glass bottles at −20 °C.

### 3.3. Sample Preparation

For sample preparation, 0.1g of flour sample was weighted in an Eppendorf tube and 1 mL of MeOH: H_2_O (80:20, *v*/*v*) was added for the extraction of bioactive compounds. There was no incubation applied to the samples. The mixture was vortexed for 1 min and then centrifuged at 14,000 rpm at 25 °C for 10 min. Then, the extract was collected and filtered using 0.22 μm nylon syringe filters (Captiva, Agilent Technologies, Santa Clara, CA, USA). Hesperidin was as added as an internal standard at a 1 mg/Kg concentration level to monitor instrument response, and the samples were then directly injected into the chromatographic system.

### 3.4. Instrumental Analysis

Chromatographic analysis was conducted using an ExionAC LC system (SCIEX, Framingham, MA, USA) coupled with a quadrupole time-of-flight (QTOF) mass spectrometer. The X500R Q-TOF mass spectrometer (SCIEX, Framingham, MA, USA) equipped with an electrospray ionization (ESI) turboV^TM^ source was connected to the LC system and it was operated in the negative ionization mode. TOF–MS and TOF–MS/MS data were acquired using the dependent acquisition electrospray ionization mode. Nebulizer gas, heater gas, and curtain gas pressures were set at 55 psi, 50 psi, and 30 psi, respectively. The spray ion spray voltage was −4500 V, with a declustering potential of 80 V. MS/MS spectra were obtained at a collision energy of 45 eV and a collision energy spread of 15 eV. External calibration was performed before the analysis with a cluster solution pro-vided by SCIEX, and additionally, the calibration solution was injected at the beginning of each run for internal calibration and once per five samples during batch acquisition. Mass spectra were recorded in the *m*/*z* range from 50 to 1000, at an accumulation time of 0.25 s. MS/MS experiments were conducted in the data-dependent acquisition mode, at an accumulation time of 0.08 s for the 10 most abundant precursor ions per full scan. Sample acquisition was monitored by the SCIEX OS software v. 3.4.5. Extraction ion chromatograms were generated using the SCIEX OS software. The established parameters were as follows: mass accuracy window, 5 ppm; S/N threshold, 3; minimum area threshold, 1000; minimum intensity threshold, 500.

Chromatographic separation was carried out using a C18 column (2.1 × 100 mm, 2.6 µm) from Fortis (Cheshire, UK), thermostated at 40 °C. The mobile phase (A) was an aqueous solution with 0.1% formic acid, and mobile phase (B) consisted of a methanolic solution with 0.1% formic acid. The adopted gradient elution program began with 1% organic phase (B) at a flow rate of 0.2 mL min^−1^ for one minute, gradually increasing to 39% over the next four minutes, then to 95% (12–15 min), and maintaining this composition for the subsequent three minutes (flow rate of 0.4 mL min^−1^). Subsequently, the organic phase increased gradually to 99% within one minute at a flow rate of 0.2 mL min^−1^ and remained constant for an additional four minutes (16–20 min). Finally, the system was returned to its initial conditions (1% B–99% A) within 0.1 min (flow rate decreased to 0.2 mL min^−1^) to re-equilibrate the column for 5 min before the next injection.

### 3.5. Screening Methodology

**Figure 6:**
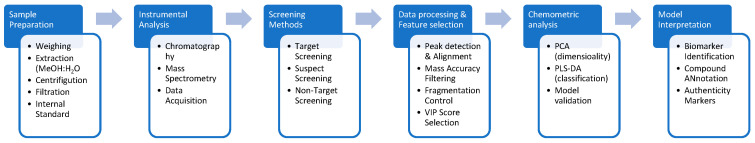
Screening workflow.

In LC-QTOF-MS/MS methodologies, target, suspect, and non-target screening are complementary approaches for compound detection. Target screening focuses on identifying and quantifying known compounds using reference databases. Suspect screening searches for expected compounds without reference standards, relying on predicted molecular formulas and fragmentation patterns. Non-target screening detects unknown compounds without prior assumptions, requiring advanced data processing and statistical analysis. Together, these approaches enable comprehensive profiling: non-target screening uncovers novel compounds, suspect screening enables tentative identification, and target screening ensures high-confidence confirmation and quantification [68]. The screening workflow for this study is depicted in Figure 6.

#### 3.5.1. Target Screening

A target list comprising ten significant phenolic acids commonly found in plant-based foods, eighteen flavonoids present in various plant parts such as seeds and roots, and two methoxyphenols was established. This target list is detailed in Table S1 of the Supplementary Materials, and the analytical standards are described in Section 3.2. The classification of these compounds was conducted using FoodDB [69]. For each target compound, extracted ion chromatograms (EICs) of the precursor ions were generated and assessed across the samples using the Analytics package within the SCIEX OS software. The screening of target compounds in the samples was based on predefined parameters, including mass accuracy of the precursor ion and the MS/MS fragments with a selection window of 5 ppm, retention time tolerance (tR < 0.2 min), a response peak area threshold of above 1000, and peak intensity of at least 1000. Mean and standard deviation were calculated with the IBM SPSS Statistics v.23 software (Armonk, NY, USA: IBM Corp).

#### 3.5.2. Suspect Screening

For suspect screening, a database was compiled from the existing literature containing phenolic compounds previously identified in grape and olive matrices. This database aimed to detect the presence of these compounds in the samples. The in-house suspect database comprised 74 compounds from grapes (stems, skins, and seeds) and 90 compounds from olives, olive trees, and olive oil. Tables S2 and S3 in the Supplementary Materials display the suspect lists, and compound classification was conducted using FoodDB [69].

Upon the detection of a peak in the matrix, the presence of the suspect compound was determined through the analysis of MS/MS fragments against those in mass spectral libraries. Additionally, in silico fragmentation tools such as MetFrag [33] and MassBank [34] were utilized. MetFrag was employed using the neutral exact mass with a mass error of 5 ppm and the appropriate ionization mode, while MassBank employed the compound name, exact mass (tolerance = 0.3), and molecular formula in the negative ionization mode. Similar to target analysis, mean and standard deviation were calculated with the IBM SPSS Statistics v.23 software (Armonk, NY, USA: IBM Corp).

#### 3.5.3. Non Target Screening

Non-target screening involves detecting peaks and identifying compounds without prior information or available standards. The non-target screening process utilized the Analytics SCIEX OS software. Within this workflow, the non-targeted screening algorithm was selected. Peaks were provisionally identified based on mass accuracy, with a maximum threshold set at 5 ppm, and a fragmentation mass error of 10 ppm. For library searches, the smart confirmation search algorithm was chosen, with results sorted by purity.

Given the substantial amount of data generated by non-target analysis, the utilization of chemometric tools (CIMCB package v. 2.1.2 (https://github.com/CIMCB/cimcb, accessed on 20 March 2025)) becomes essential for interpretation. A multivariate statistical analysis was performed using both supervised and unsupervised models, specifically principal component analysis (PCA) and partial least squares-discriminant analysis (PLS-DA). The primary objective of PCA is to decrease the dimensionality of a dataset with many interrelated variables, while preserving as much of the original variation in the data as possible [70]. Additionally, PLS-DA was used because of its ability to achieve dimensionality reduction while fully considering the class labels, and it is suitable for feature selection and classification [71]. Variable importance in projection (VIP) scores are employed to identify the most significant features for class discrimination, incorporating bootstrap iterations offers a statistically robust approach compared to relying on a single random split of the data. Bootstrap aggregation, by generating numerous replicates of the dataset via sampling with replacement, addresses inherent variability within the data. This iterative process yields an ensemble of VIP scores, from which the mean is calculated. This mean VIP score provides a more reliable estimate of feature importance compared to a single instance, as it attenuates the influence of any single random split and captures the variability in the VIP score distribution. Consequently, features with consistently high mean VIP scores across bootstrap iterations can be confidently identified as the most important for model performance and subsequent interpretations [72,73]. A VIP cut-off value of 1.00 was applied to identify the most influential phenolic compounds. Subsequently, the model’s validity was assessed using cross-validation parameters (R2Y: goodness of fit and Q2Y: goodness of prediction) and permutation plots. Loading plots were used to evaluate the impact of each mass feature on a principal component. Features with a high degree of influence were further investigated, with plausible formulas proposed using the Formula Finder tool in the SCIEX OS software. The SCIEX Natural Products High Resolution MS/MS Spectral Library then suggested viable candidates. Only matches with a Formula Finder or Library Match Score of 50.0 or higher were considered high-confidence proposed formulas or compounds [74].

## Figures and Tables

**Table 2:** Non-target screening results for grape seed flour (GSF) samples, including mass features with a variable importance in projection (VIP) score above 1.0, along with their library matches and plausible molecular formulas, tentative analytes, and Formula Finder scores.

[M − H]^−^/RT	VIP	Plausible Molecular Formula	Tentative Compound	Phytochemical Class	Formula Finder Score	MassBank ID/Reference
149.0088/0.99	1.908055	C_4_H_6_O_6_	Tartaric acid	Organic acids	92.8	KO001902
195.0504/0.98	4.165611	C_6_H_12_O_7_	Gluconic acid	Organic acids	88.6	KO000864
247.0249/5.13	1.542148	C_13_H_12_OS_2_			87.9	
255.2311/13.96	1.687796	C_16_H_32_O_2_			65.6	
265.1460/13.51	1.05701	C_12_H_26_O_4_S			79.3	
265.1475/14.05	1.072462				88.4	
279.2309/13.79	6.86827				92.5	
281.2465/14.03	7.233686				91.4	
282.2507/14.01	2.547436				89.4	
283.2635/14.34	1.250575				78.5	
289.0702/5.24	12.24895				82.6	
289.0705/4.50	14.487	C_15_H_14_O_6_	Catechin	Flavonoid	97.8	BS003014
291.0146/5.12	3.056952	C_13_H_8_O_8_	Brevifolincarboxylic acid	Polyphenol	92.5	[64]
293.1240/5.22	1.667305	C_12_H_22_O_8_			89.9	
293.1791/15.70	1.215117					
295.2263/13.06	5.327168				88.2	
297.2422/13.05	2.411256	C_18_H_34_O_3_			91.4	
300.9982/6.84	8.084582		Ellagic acid	Polyphenol	92.2	NGA02837
301.0345/8.09	1.688408	Quercetin (C_15_H_10_O_7_)	Quercetin	Flavonoid	93.5	PR100233
309.1727/14.34	1.722773					
311.2221/11.84	1.983825					
313.2368/12.20	8.325131	C_18_H_36_O_5_	Phloionolic acid	Triterpenoid	88.4	
314.2411/12.21	1.989586	C_12_H_29_N_9_O				
315.0875/7.06	1.83894	C_17_H_16_O_6_	Persicogenin	Flavonoid	95.6	[65]
315.2528/12.65	1.635675					
329.2327/10.38	1.132457					
341.1077/0.98	4.904493					
341.1086/1.80	1.266851	C_13_H_26_O_6_S_2_			82.2	
366.1190/5.82	1.098081	C_17_H_21_NO_8_			78.4	
380.9556/6.29	1.721243	C_15_H_10_O_6_S_3_			73.5	
387.1144/0.96	1.194701					
409.2340/14.65	1.206799	C_21_H_34_N_2_O_6_			78.8	
433.0408/6.51	2.234584	C_20_H_10_N_4_O_8_			75.5	
433.2336/14.26	2.630344					
439.0650/6.75	4.659214	C_22_H_16_O_10_			89.2	
439.0656/7.42	5.428307	C_22_H_16_O_10_	Amurensisin	Flavonoid	82.3	[66]
439.0841/0.99	1.663483	C_15_H_16_N_6_O_10_			78.5	
440.0712/7.56	1.214139	C_16_H_11_N_9_O_7_			77.9	
441.0808/5.73	8.944765	C_22_H_18_O_8_	Epicatechin gallate	Flavonoid	81.3	BS003900
442.0865/5.87	2.155587	C_16_H_12_N_9_O_7_			82.2	
453.0676/4.78	1.773836	C_20_H_14_N_4_O_9_			83.4	
455.3510/13.60	2.632012	C_30_H_48_O_3_	Oleanolic acid	Triterpenoid acid	91.2	TY000153
461.2655/15.86	2.204115	C_25_H_38_N_2_O_6_			78.4	
463.0515/5.80	1.35816	C_21_H_12_N_4_O_9_			90.4	
476.2760/13.28	1.116856	C_25_H_39_N_3_O_6_			95.6	
477.0664/6.62	1.626783	C_21_H_18_O_13_	Quercetin 3-*O*-glucuronide (Miquelianin)	Flavonoid glycoside	97.7	PR100978
479.0829/6.15	1.042092	C_21_H_20_O_13_	Myricetin-3-*O*-glucoside	Flavonoid glycoside	92.2	[67]
571.2874/15.62	2.853407	C_40_H_36_N_4_			83.5	
577.1328/4.71	5.115305	C_27_H_17_N_10_O_6_			87.4	
577.1332/4.14	5.979778	C_27_H_18_N_10_O_6_			86.5	
577.1347/5.87	1.222666	C_23_H_26_N_6_O_10_S			88.2	
578.1376/4.74	1.749601	C_24_H_21_N_9_O_9_			92.1	
578.1378/4.11	2.151917	C_24_H_21_N_9_O_9_			86.4	
595.2864/14.96	2.240486	C_41_H_40_O_4_			98.1	
729.1441/4.95	2.275241	C_31_H_42_N_2_O_8_S_5_			84.2	
865.1964/4.97	1.576048	C_47_H_46_O_8_S_4_			83.4	

## Data Availability

Data are available upon request.

## Supplementary Materials

The following supporting information can be downloaded at: https://www.mdpi.com/article/10.3390/molecules30071527/s1. Table S1. Target screening list of compounds; Table S2. Calibration values (peak area) for target screening compounds; Table S3. Target screening results for commercial grape seed flour (GSFC) and grape seed flour from Lemnos (GSFL); Table S4. Suspect screening list for grape seed flour; Table S5. Suspect screening results for commercial grape seed flour (GSFC) and grape seed flour from Lemnos (GSFL); Table S6. Target screening results for commercial olive stone flour (OSFC) and olive stone flour from Lemnos (OSFL); Table S7. Suspect screening list for olive stone flour; Table S8. Suspect screening results for commercial olive stone flour (OSFC) and olive stone flour from Lemnos (OSFL); Figure S1. Extracted ion chromatogram, MS, and MS/MS spectra of apigenin in GSFC; Figure S2. Extracted ion chromatogram, MS, and MS/MS spectra of caffeic acid in GSFL; Figure S3. Extracted ion chromatogram, MS, and MS/MS spectra of catechin in GSFL; Figure S4. Extracted ion chromatogram, MS, and MS/MS spectra of chrysin in GSFC; Figure S5. Extracted ion chromatogram, MS, and MS/MS spectra of coumaric acid in GSFL; Figure S6. Extracted ion chromatogram, MS, and MS/MS spectra of diosmin in GSFL; Figure S7. Extracted ion chromatogram, MS, and MS/MS spectra of epicatechin gallate in GSFL; Figure S8. Extracted ion chromatogram, MS, and MS/MS spectra of epigallocatechin in GSFC; Figure S9. Extracted ion chromatogram, MS, and MS/MS spectra of gallic acid in GSFC; Figure S10. Extracted ion chromatogram, MS, and MS/MS spectra of myricetin in GSFL; Figure S11. Extracted ion chromatogram, MS, and MS/MS spectra of protocatechuic acid in GSFC; Figure S12. Extracted ion chromatogram, MS, and MS/MS spectra of quercetin in GSFL; Figure S13. Extracted ion chromatogram, MS, and MS/MS spectra of quercitrin in GSFC; Figure S14. Extracted ion chromatogram, MS, and MS/MS spectra of rutin in GSFL; Figure S15. Extracted ion chromatogram, MS, and MS/MS spectra of apigenin in OSFL; Figure S16. Extracted ion chromatogram, MS, and MS/MS spectra of caffeic acid in OSFC; Figure S17. Extracted ion chromatogram, MS, and MS/MS spectra of chrysin in OSFC; Figure S18. Extracted ion chromatogram, MS, and MS/MS spectra of coumaric acid in OSFC; Figure S19. Extracted ion chromatogram, MS, and MS/MS spectra of diosmin in OSFL; Figure S20. Extracted ion chromatogram, MS, and MS/MS spectra of ferulic acid in OSFL; Figure S21. Extracted ion chromatogram, MS, and MS/MS spectra of gallic acid in OSFL; Figure S22. Extracted ion chromatogram, MS, and MS/MS spectra of luteolin in OSFL; Figure S23. Extracted ion chromatogram, MS, and MS/MS spectra of quercetin in OSFL; Figure S24. Extracted ion chromatogram, MS, and MS/MS spectra of quercitrin in OSFL; Figure S25. Extracted ion chromatogram, MS, and MS/MS spectra of rutin in OSFL; Figure S26. Extracted ion chromatogram, MS, and MS/MS spectra of sinapic acid in OSFC; Figure S27. Extracted ion chromatogram, MS, and MS/MS spectra of taxifolin in OSFL; Figure S28. Extracted ion chromatogram, MS, and MS/MS spectra of vanillic acid in OSFC; Figure S29. Extracted ion chromatogram, MS, and MS/MS spectra of vanillin in OSFL; Figure S30. Loadings plot for GSFC and GSFL corresponding to PCA; Figure S31. Loadings plot for OSFC and OSFL corresponding to PCA; Figure S32. Loadings plot for GSFC and GSFL corresponding to the PLS-DA model; Figure S33. Loadings plot for OSFC and OSFL corresponding to the PLS-DA model; Figure S34. VIP scores corresponding to the PLS-DA model for GSFC and GSFL; Figure S35. VIP scores corresponding to the PLS-DA model for OSFC and OSFL; Figure S36. Permutation test corresponding to the PLS-DA model for GSFC and GSFL; Figure S37. Permutation test corresponding to the PLS-DA model for OSFC and OSFL; Figure S38. ROC curve corresponding to the PLS-DA model for GSFC and GSFL (LV1, LV2: Explanatory variables); Figure S39. ROC curve corresponding to the PLS-DA model for OSFC and OSFL (LV1, LV2: Explanatory variables).
