# Supplementary material for: A Novel Liquid Chromatographic Time-of-Flight Tandem Mass Spectrometric Method for the Determination of Secondary Metabolites in Functional Flours Produced from Grape Seed and Olive Stone Waste
Source: Molecules. 2025 Mar 29;30(7):1527. doi: 10.3390/molecules30071527 (PMC11990682; doi:10.3390/molecules30071527)
Supplement: Supplementary file 1 [file molecules-30-01527-s001.zip › molecules-3495158-supplementary.pdf]

# Supplementary material

## A Novel Liquid Chromatographic Time-of-Flight Tandem Mass Spectrometric Method for the Determination of Secondary Metabolites in Functional Flours Produced from Grape Seed and Olive Stone Waste

Achilleas Panagiotis Zalidis [1], Natasa P. Kalogiouri [2,*], Ioannis Mourtzinos [3], Dimitris Sarris [1] and Konstantinos Gkatzionis [1,*]

1 Laboratory of Consumer and Sensory Perception of Food & Drinks, Department of Food Science and Nutrition, University of the Aegean, Metropolite Ioakeim 2, 81400 Myrina, Greece; achilles.zalidis@gmail.com (A.P.Z.); dsarris@aegean.gr (D.S.)

2 Laboratory of Analytical Chemistry, School of Chemistry, Aristotle University of Thessaloniki, 54124 Thessaloniki, Greece

3 Laboratory of Food Chemistry and Biochemistry, Department of Food Science and Technology, School of Agriculture, Aristotle University of Thessaloniki, 54124 Thessaloniki, Greece; mourtzinos@agro.auth.gr

* Correspondence: kalogiourin@chem.auth.gr (N.P.K.); kgkatzionis@aegean.gr (K.G.)

**Table S1. Target screening list of compounds**

| Compound | Molecular formula | Class |
|---|---|---|
| **Apigenin** | $C_{15}H_{10}O_5$ | Flavonoids |
| **Caffeic acid** | $C_9H_8O_4$ | Cinnamic acids and derivatives |
| **Catechin** | $C_{15}H_{14}O_6$ | Flavonoids |
| **Chrysin** | $C_{15}H_{10}O_4$ | Flavonoids |
| **Cinammic acid** | $C_{14}H_{10}N_2O_4S$ | Cinnamic acids and derivatives |
| **Coumaric acid** | $C_9H_8O_3$ | Cinnamic acids and derivatives |
| **Diosmin** | $C_{28}H_{32}O_{15}$ | Flavonoids |
| **Epicatechin** | $C_{15}H_{14}O_6$ | Flavonoids |
| **Epigallocatechin** | $C_{15}H_{14}O_7$ | Flavonoids |
| **Ferulic acid** | $C_{10}H_{10}O_4$ | Hydroxycinnamic acid |
| **Gallic acid** | $C_7H_6O_5$ | Benzene and substituted derivatives |
| **Epicatechin gallate** | $C_{22}H_{18}O_{10}$ | Flavonoids |
| **Hesperidin** | $C_{28}H_{34}O_{15}$ | Flavonoids |
| **Kaempferol** | $C_{15}H_{10}O_6$ | Flavonoids |
| **Luteolin** | $C_{15}H_{10}O_6$ | Flavonoids |
| **Myricetin** | $C_{15}H_{10}O_8$ | Flavonoids |
| **Myricitrin** | $C_{21}H_{20}O_{12}$ | Flavonoids |
| **Naringin** | $C_{27}H_{32}O_{14}$ | Flavonoids |
| **Quercetin** | $C_{15}H_{10}O_7$ | Flavonoids |
| **Quercitrin** | $C_{21}H_{20}O_{11}$ | Flavonoids |
| **Rosmarinic acid** | $C_{18}H_{16}O_8$ | Cinnamic acids and derivatives |
| **Protocatechuic acid** | $C_7H_6O_4$ | hydroxybenzoic acid derivatives |
| **Rutin (Quercetin rutinoside)** | $C_{27}H_{30}O_{16}$ | Flavonoids |
| **Sinapic acid** | $C_{11}H_{12}O_5$ | Hydroxycinnamic acids |
| **Syringaldehyde** | $C_9H_{10}O_4$ | Phenols |
| **Syringic acid** | $C_9H_{10}O_5$ | Benzene and substituted derivatives |
| **Taxifolin** | $C_{15}H_{12}O_7$ | Flavonoids |
| **Vanillic acid** | $C_8H_8O_4$ | Benzene and substituted derivatives |
| **Vanillin** | $C_8H_8O_3$ | Phenols |

## Table S2. Calibration values (peak area) for target screening compounds

| Compound | y= ax + b | $R^2$ |
|---|---|---|
| Apigenin | y = 57,365,423x + 2,154,266 | 0.9847 |
| Caffeic acid | y = 4,638,094x + 851,136 | 0.9934 |
| Catechin | y = 4,637,676x + 1,055,638 | 0.9922 |
| Cinammic acid | y = 564867x + 36629 | 0.9909 |
| Coumaric acid | y = 2,311,151x + 793,574 | 0.9905 |
| Diosmin | y = 1,512,811x + 147,425 | 0.9816 |
| Epicatechin | y = 5,547,103x + 965,164 | 0.9880 |
| Epigallocatechin | y = 5,529,078x + 921,431 | 0.9960 |
| Ferulic acid | y = 1,529,062x + 259,933 | 0.9917 |
| Gallic acid | y = 554,459x + 173,049 | 0.9903 |
| Epicatechin gallate | y = 55827x + 19936 | 0.9919 |
| Hesperidin | y = 5,647,435x + 1,166,728 | 0.9921 |
| Kaempferol | y = 39,424,615x + 845,756 | 0.9932 |
| Luteolin | y = 35,306,262x + 2,775,288 | 0.9883 |
| Myricetin | y = 18,249,843x + 934,951 | 0.9945 |
| Myricitrin | y = 8,351,664x + 1,403,352 | 0.9939 |
| Naringin | y = 30,390,744x + 370,121 | 0.9961 |
| Quercetin | y = 9,878,454x + 577,540 | 0.9924 |
| Quercitrin | y = 1,383,477x + 368,884 | 0.9915 |
| Rosmarinic acid | y = 8,224,822x + 442,237 | 0.9950 |
| Protocatechuic acid | y = 2,205,956x + 255,348 | 0.9961 |
| Rutin | y = 576,033x + 188,555 | 0.9926 |
| Sinapic acid | y = 908533x + 279739 | 0.9910 |
| Syringaldehyde | y = 632779x + 15367 | 0.9846 |
| Syringic acid | y = 504988x + 68967 | 0.9971 |
| Taxifolin | y = 57,365,423x + 2,154,266 | 0.9847 |
| Vanillic acid | y = 4,638,094x + 851,136 | 0.9934 |
| Vanillin | y = 4,637,676x + 1,055,638 | 0.9922 |

**Table S3. Target screening results for commercial grape seed flour (GSFC) and grape seed flour from Lemnos (GSFL)**

| Compound | Molecular Formula | $[M-H]^-$ standard | $[M-H]^-$ experimental GSFC | $[M-H]^-$ experimental GSFL | Rt (min) | ΔRt | Fragments m/z | Elemental Formula | GSFC ±SD mg/kg | GS FL ±SD mg/kg |
|---|---|---|---|---|---|---|---|---|---|---|
| **Apigenin** | $C_{15}H_{10}O_5$ | 269.0448 | 269.0460 | 269.0460 | 9.04 | 0 | 63.0238<br>65.0027<br>107.0133<br>117.0335<br>149.0237<br>159.0448<br>225.0553 | $[C_5H_4]-H^-$<br>$[C_4H_3O-H]-H^-$<br>$[C_6H_4O_2]-H^-$<br>$[C_8H_6O]-H^-$<br>$[C_8H_5O_3]^-$<br>$[C_{10}H_7O_2]^-$<br>$[C_{14}H_9O_3]^-$ | **0.019 ±0.006** | **0.152 ±0.004** |
| **Caffeic acid** | $C_9H_8O_4$ | 179.0348 | 179.0356 | 179.0352 | 5.12 | 0.1 | 65.0404<br>79.0552<br>134.0375<br>135.0448 | $[C_4H_2O]-H^-$<br>$[C_6H_5+2H]^-$<br>$[C_8H_7O_2]-H^-$<br>$[C_8H_7O_2]^-$ | **0.111 ±0.02** | **1.08 ±0.036** |
| **Catechin** | $C_{15}H_{14}O_6$ | 289.0718 | 289.0713 | 289.0715 | 4.51 | +0.03 | 97.0293<br>109.0294<br>123.0449<br>125.0245<br>151.0401<br>187.0405<br>203.0704<br>221.0814<br>245.0804 | $[C_5H_5O_2]^-$<br>$[C_6H_5O_2]^-$<br>$[C_7H_6O_2+H]^-$<br>$[C_6H_4O_3+H]^-$<br>$[C_8H_8O_3]-H^-$<br>$[C_{11}H_9O_3-H]-H^-$<br>$[C_{12}H_{10}O_3+H]^-$<br>$[C_{12}H_{12}O_4+H]^-$<br>$[C_{14}H_{11}O_4+2H]^-$ | **48.1 ±1.281** | **441.8 ±7.344** |
| **Coumaric acid** | $C_9H_8O_3$ | 163.0400 | 163.0402 | 163.0402 | 5.97 | 0 | 65.0399<br>91.0556<br>93.0346<br>117.0342<br>119.0501 | $[C_5H_4+H]^-$<br>$[C_6H_5O]^-$<br>$[C_8H_7O-H]-H^-$<br>$[C_8H_7O]^-$ | **0.360 ±0.019** | **1.37 ±0.053** |
| **Chrysin** | $C_{15}H_{10}O_4$ | 253.0510 | 253.0507 | 253.0507 | 10.54 | -0.01 | 63.0238<br>65.0034<br>107.0145<br>143.0503<br>145.0294<br>209.0614 | $[C_5H_5-H]-H^-$<br>$[C_6H_4O_2]-H^-$<br>$[C_{10}H_7O]^-$<br>$[C_{14}H_9O_2]^-$ | | |

| | | | | | | | | | | |
|---|---|---|---|---|---|---|---|---|---|---|
| | | | | | | | 253.0513 | | | |
| **Epicatechin** | $C_{15}H_{14}O$_ | 289.0718 | 289.0715 | 289.0713 | 5.24 | -0.03 | 83.0135<br>95.0498<br>97.0290<br>123.0441<br>151.0393<br>203.0702<br>221.0812 | $[C_4H_4O_2]-H^-$ $[C_6H_7O]^-$<br>$[C_5H_5O_2]^-$<br>$[C_7H_6O_2+H]^-$<br>$[C_8H_8O_3]-H^-$<br>$[C_{12}H_{10}O_3+H]^-$<br>$[C_{12}H_{12}O_4+H]^-$ | **40.6<br>±1.2** | **192<br>±1.604** |
| **Epigallocatechin** | $C_{15}H_{14}O_7$ | 305.0665 | 305.0673 | 305.0672 | 4.43 | +0.08 | 83.0136<br>109.0294<br>125.0244<br>159.0456<br>161.0249<br>261.0782 | $[C_4H_4O_2]-H^-$<br>$[C_6H_5O_2]^-$<br>$[C_6H_4O_3+H]^-$<br>$[C_{10}H_9O_2-H]-H^-$<br>$[C_9H_8O_3-2H]-H^-$<br>$[C_{14}H_{12}O_5+H]^-$ | **1.08<br>±0.069** | **13.2<br>±0.255** |
| **Ferulic acid** | $C_{10}H_{10}O_4$ | 193.0505 | 193.0509 | 193.0507 | 6.25 | -0.01 | 106.0414<br>132.0210<br>133.0301<br>134.0375<br>178.0276 | $[C_7H_6O]^-$<br>$[C_8H_6O_2-H]-H^-$<br>$[C_8H_6O_2]-H^-$<br>$[C_8H_6O_2]^-$<br>$[C_9H_7O_4]-H^-$ | **0.650<br>±0.041** | **1.25<br>±0.104** |
| **Gallic acid** | $C_7H_6O_5$ | 169.0141 | 169.0145 | ND | 1.04 | +0.09 | 69.0344<br>123.0088<br>124.0168<br>125.0243 | $[C_4H_3O+2H]^-$<br>$[C_6H_5O_3-H]-H^-$<br>$[C_6H_5O_3]-H^-$<br>$[C_6H_5O_3]^-$ | **17.7<br>±1.711** | **3.33<br>±0.897** |
| **Epicatechin gallate** | $C_{22}H_{18}O_{10}$ | 441.0831 | 441.0824 | 441.0817 | 5.74 | -0.01 | 97.0294<br>109.0291<br>124.0163<br>203.0711<br>245.0811<br>303.0517 | $[C_5H_5O_2]^-$<br>$[C_6H_5O_2]^-$<br>$[C_6H_5O_3]-H^-$<br>$[C_{12}H_{10}O_3+H]^-$<br>$[C_{14}H_{11}O_4+2H]^-$<br>$[C_{15}H_{12}O_7]-H^-$ | **2926<br>±0.456** | **15442<br>±0.992** |
| **Kaempferol** | $C_{15}H_{10}O_6$ | 285.0398 | 285.0402 | 285.0402 | 8.92 | +0.01 | 65.0030<br>93.0343<br>117.0344<br>151.0037<br>211.0406<br>229.0509 | $[C_4H_3O-H]-H^-$<br>$[C_6H_5O]^-$<br>$[C_8H_5O]^-$<br>$[C_7H_4O_4]-H^-$<br>$[C_{13}H_8O_3]-H^-$<br>$[C_{13}H_9O_4]^-$ | **3.22<br>±0.313** | **2.20<br>±0.06** |
| **Luteolin** | $C_{15}H_{10}O_6$ | 285.0401 | 285.0406 | 285.0408 | 8.36 | +0.01 | 65.0036<br>107.0138<br>132.0221 | $[C_4H_3O-H]-H^-$<br>$[C_6H_5O_2-H]-H^-$<br>$[C_8H_6O_2-H]-H^-$ | **0.463<br>±0.194** | **0.559<br>±0.016** |

| | | | | | | | | | | |
|---|---|---|---|---|---|---|---|---|---|---|
| | | | | | | | 151.0038<br>199.0402<br>217.0515<br>241.0504 | $[C_7H_4O_4]-H^-$<br>$[C_{12}H_6O_3+H]^-$<br>$[C_{12}H_9O_4]^-$<br>$[C_{14}H_9O_4]^-$ | | |
| **Myricetin** | $C_{15}H_{10}O_8$ | 317.0300 | 317.0311 | 317.0301 | 7.14 | +0.01 | 83.0137<br>109.0295<br>137.0242<br>151.0032<br>193.0146<br>271.0252 | $[C_4H_4O_2]-H^-$<br>$[C_6H_4O_2+H]^-$<br>$[C_7H_4O_3+H]^-$<br>$[C_7H_4O_4]-H^-$<br>$[C_9H_5O_5]^-$<br>$[C_{14}H_9O_6-H]-H^-$ | **0.045<br>±0.005** | **3.81<br>±0.081** |
| **Myricitrin** | $C_{21}H_{20}O_{12}$ | 463.0880 | 463.0885 | 463.0881 | 6.48 | -0.14 | 121.0301<br>151.0035<br>175.0043<br>300.0276<br>301.0343 | $[C_7H_3O_2+2H]^-$<br>$[C_7H_4O_4]-H^-$<br>$[C_9H_4O_4]-H^-$<br>$[C_{15}H_9O_7]-H^-$<br>$[C_{15}H_9O_7]^-$ | **4.85<br>±0.102** | **4.51<br>±0.065** |
| **Quercetin** | $C_{15}H_{10}O_7$ | 301.0350 | 301.0352 | 301.0351 | 8.08 | +0.01 | 65.0029<br>83.0137<br>107.0135<br>121.0292<br>151.0034<br>178.9982<br>186.0319 | $[C_4H_3O-H]-H^-$<br>$[C_4H_4O_2]-H^-$<br>$[C_6H_5O_2-H]-H^-$<br>$[C_7H_5O_2]^-$<br>$[C_7H_4O_4]-H^-$<br>$[C_8H_5O_5-H]-H^-$<br>$[C_{11}H_7O_3]-H^-$ | **8.68<br>±0.6414** | **12.6<br>±0.2499** |
| **Quercitrin** | $C_{21}H_{20}O_{11}$ | 447.0930 | 447.0929 | 447.0935 | 7.19 | +0.04 | 151.0040<br>255.0301<br>284.0326<br>285.0409<br>300.0282<br>301.0367<br>327.0529 | $[C_7H_4O_4]-H^-$<br>$[C_{14}H_9O_5-H]-H^-$<br>$[C_{15}H_9O_6]-H^-$<br>$[C_{15}H_9O_6]^-$<br>$[C_{15}H_9O_7]-H^-$<br>$[C_{15}H_9O_7]^-$<br>$[C_{17}H_{11}O_7]^-$ | **5.72<br>±1.097** | **3.88<br>±0.191** |
| **Procatechuic acid** | $C_7H_6O_4$ | 153.0192 | 153.0195 | 153.0195 | 3.26 | +0.01 | 65.0032<br>81.0343<br>91.0187<br>108.0210<br>109.0287 | $[C_4H_2O]-H^-$<br>$[C_5H_4O+H]^-$<br>$[C_6H_4O]-H^-$<br>$[C_6H_5O_2]-H^-$<br>$[C_6H_5O_2]^-$ | **6.64<br>±0.558** | **9.95<br>±0.851** |
| **Rutin** | $C_{27}H_{30}O_{16}$ | 609.1456 | 609.1470 | 609.1458 | 6.67 | +0.01 | 151.0041<br>255.0300 | $[C_7H_4O_4]-H^-$<br>$[C_{14}H_9O_5-H]-H^-$ | **1.52<br>±0.722** | **5.67<br>±0.164** |

| | | | | | | | | | | |
|---|---|---|---|---|---|---|---|---|---|---|
| | | | | | | | 271.0250<br>300.0266<br>301.0350 | $[C_{14}H_9O_6-H]-H^-$<br>$[C_{15}H_9O_7]-H^-$<br>$[C_{15}H_9O_7]^-$ | | |
| **Taxifolin** | $C_{15}H_{12}O_7$ | 303.0508 | 303.0517 | 303.0515 | 6.09 | -0.01 | 57.0339<br>83.0142<br>121.0294<br>123.0450<br>125.0244<br>175.0409<br>217.0504<br>285.0417 | $[C_3H_3O+2H]^-$<br>$[C_4H_4O_2]-H^-$<br>$[C_7H_6O_2]-H^-$<br>$[C_7H_6O_2+H]^-$<br>$[C_6H_4O_3+H]^-$<br>$[C_{10}H_7O_3]^-$<br>$[C_{12}H_8O_4+H]^-$<br>$[C_{15}H_{11}O_6-H]-H^-$ | **0.696 ±0.032** | **1.32 ±0.04** |

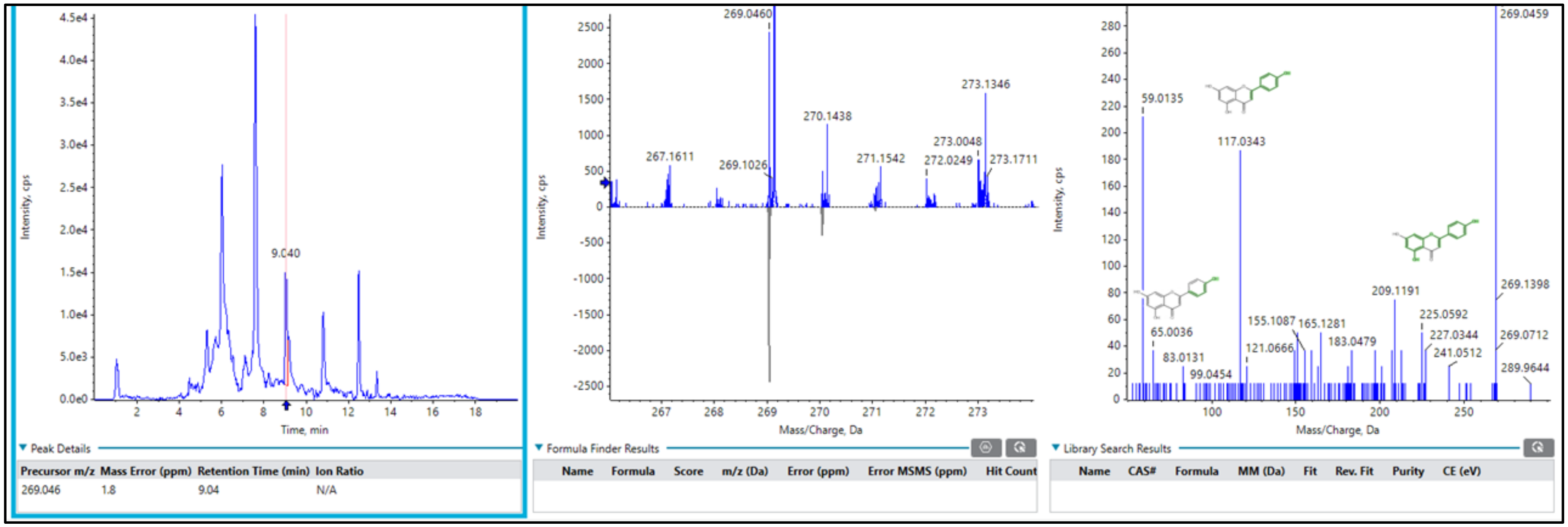


**Figure S1. Extracted ion chromatogram, MS, and MS/MS spectra of apigenin in GSFC.**

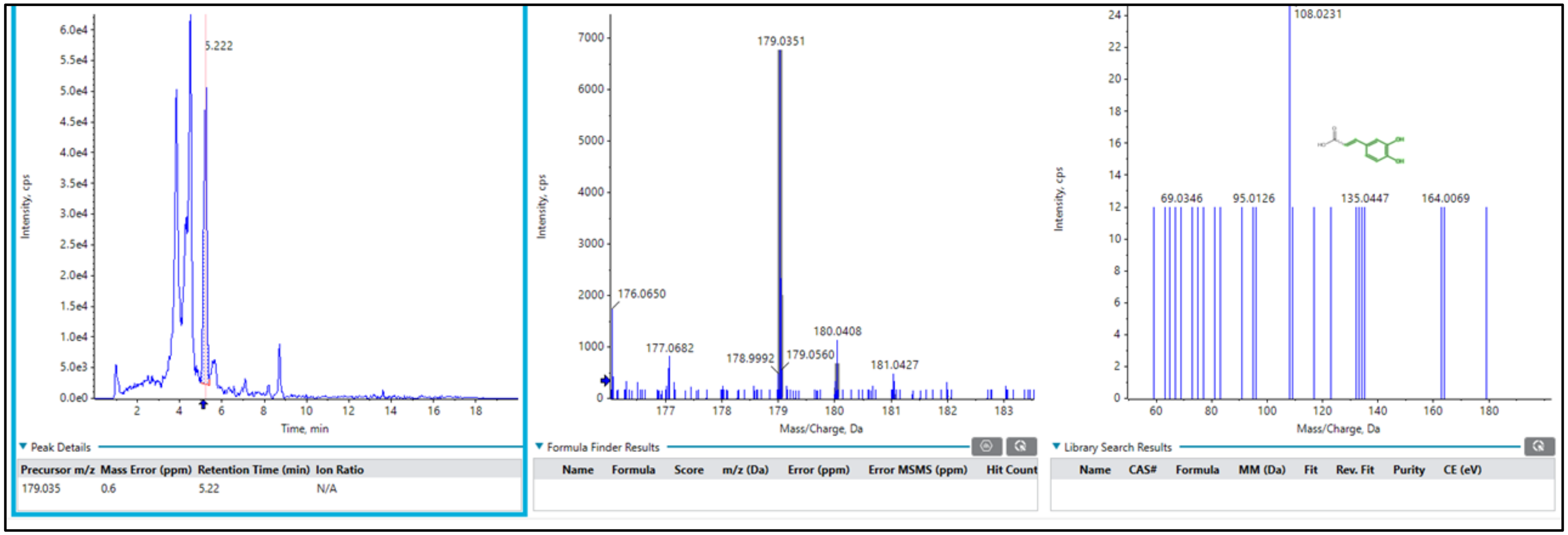


**Figure S2. Extracted ion chromatogram, MS, and MS/MS spectra of caffeic in GSFL.**

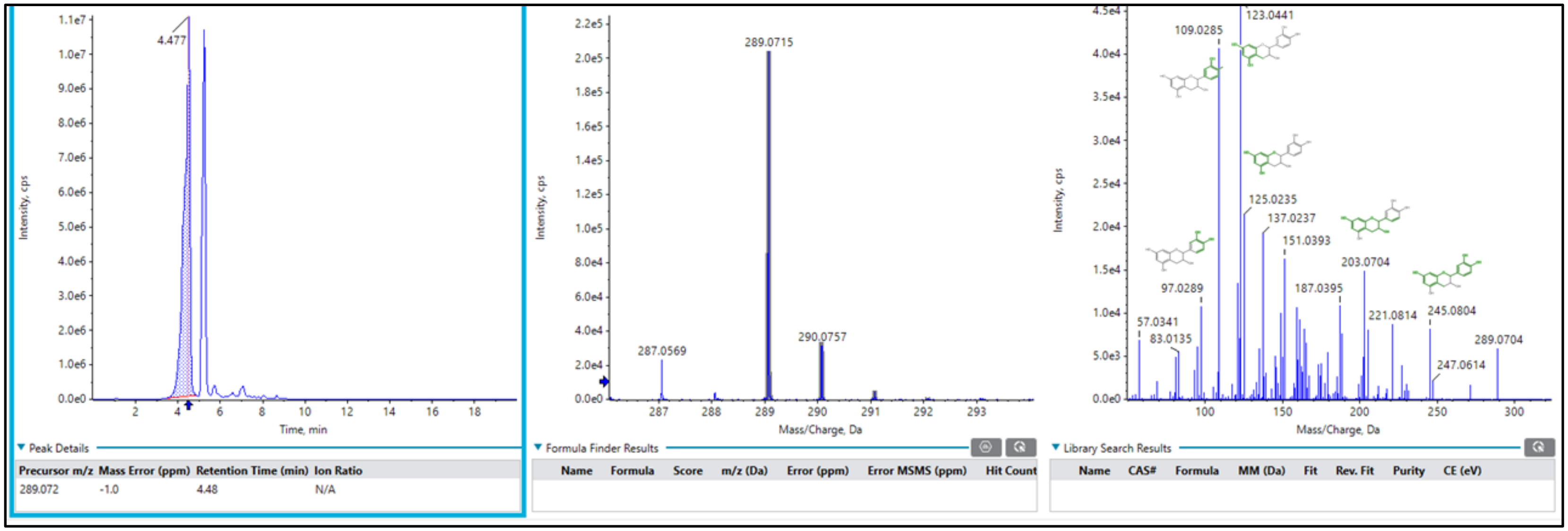


**Figure S3. Extracted ion chromatogram, MS, and MS/MS spectra of catechin in GSFL.**

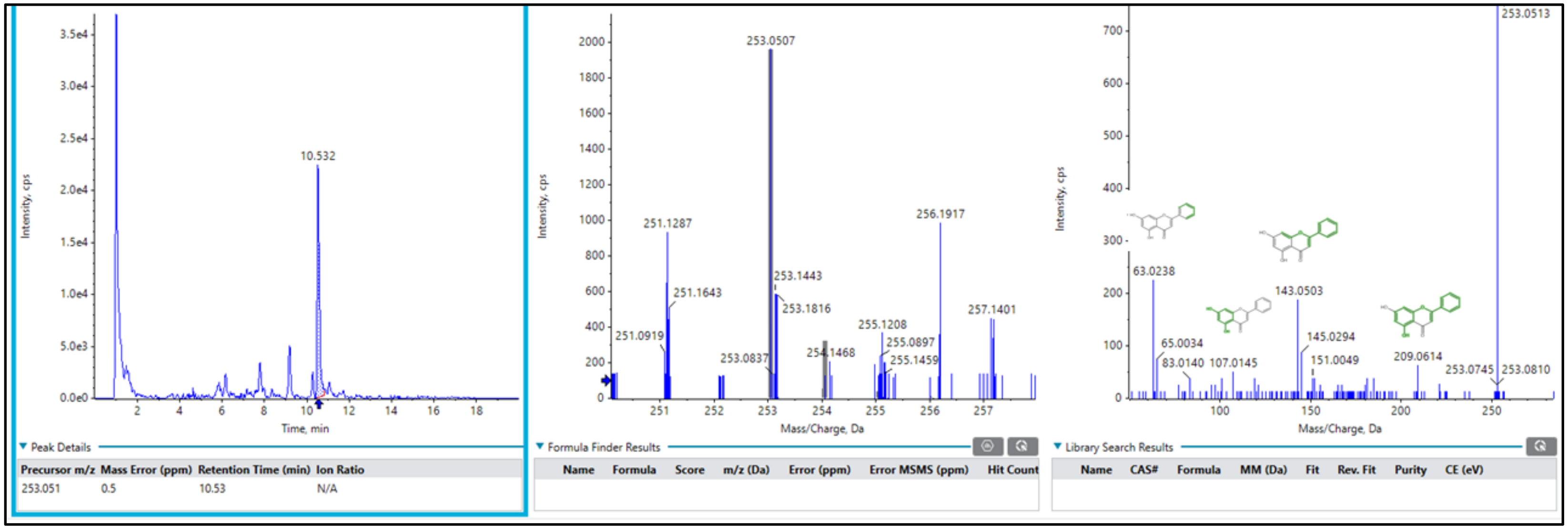


**Figure S4. Extracted ion chromatogram, MS, and MS/MS spectra of chrysin in GSFC.**

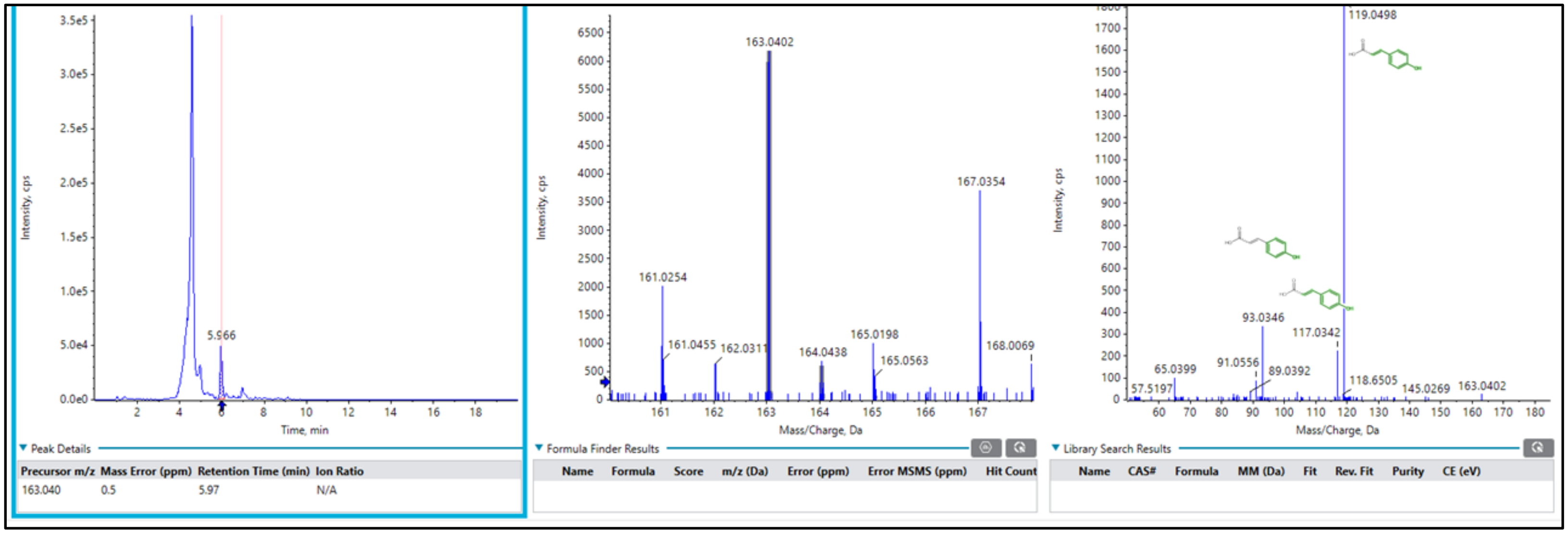


**Figure S5. Extracted ion chromatogram, MS, and MS/MS spectra of coumaric acid in GSFL.**

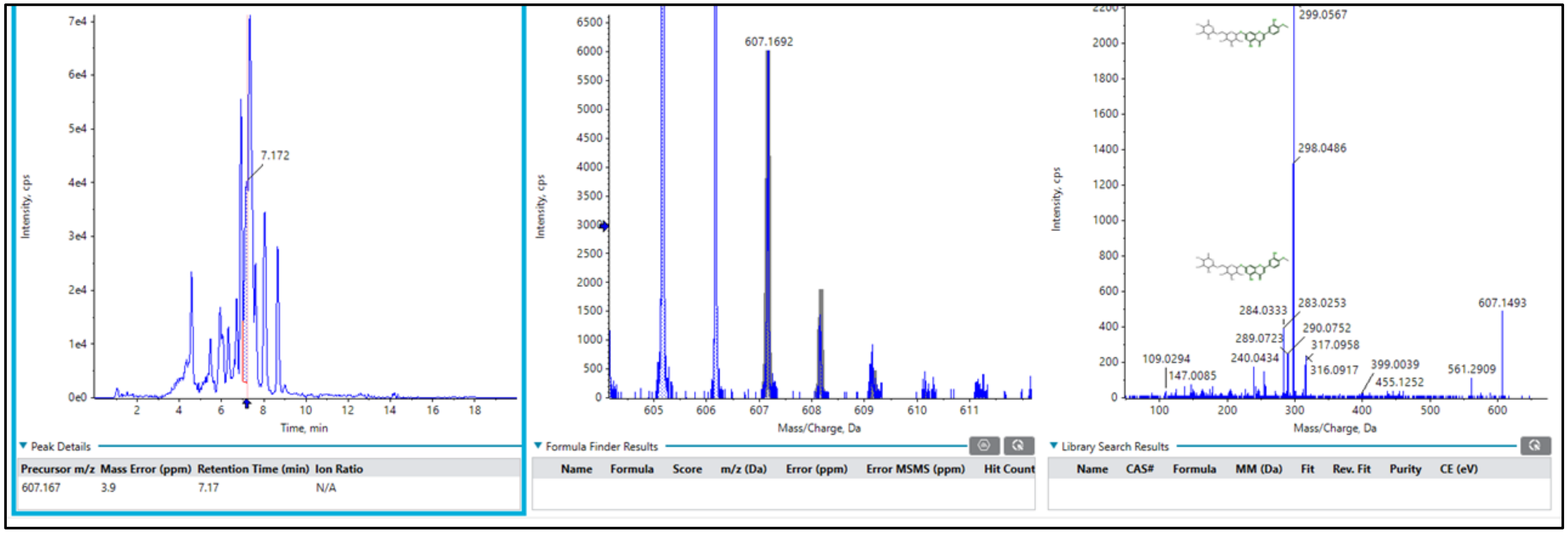


**Figure S6. Extracted ion chromatogram, MS, and MS/MS spectra of diosmin in GSFL.**

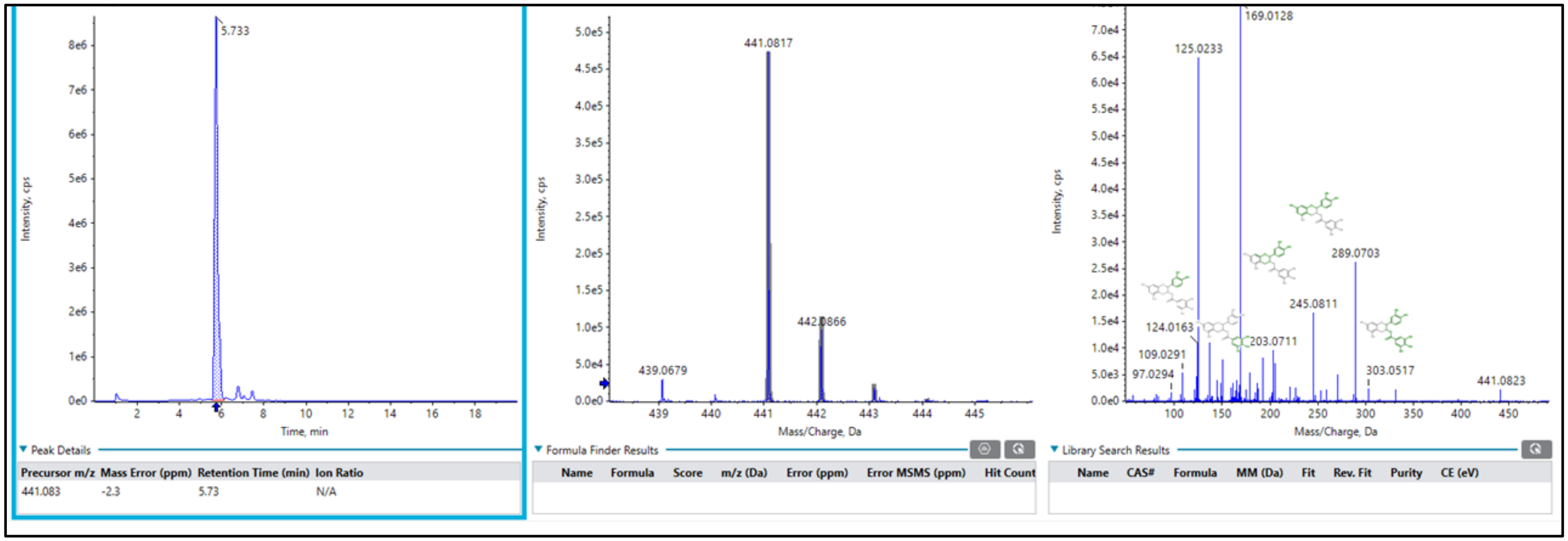


**Figure S7. Extracted ion chromatogram, MS, and MS/MS spectra of epicatechin gallate in GSFL.**

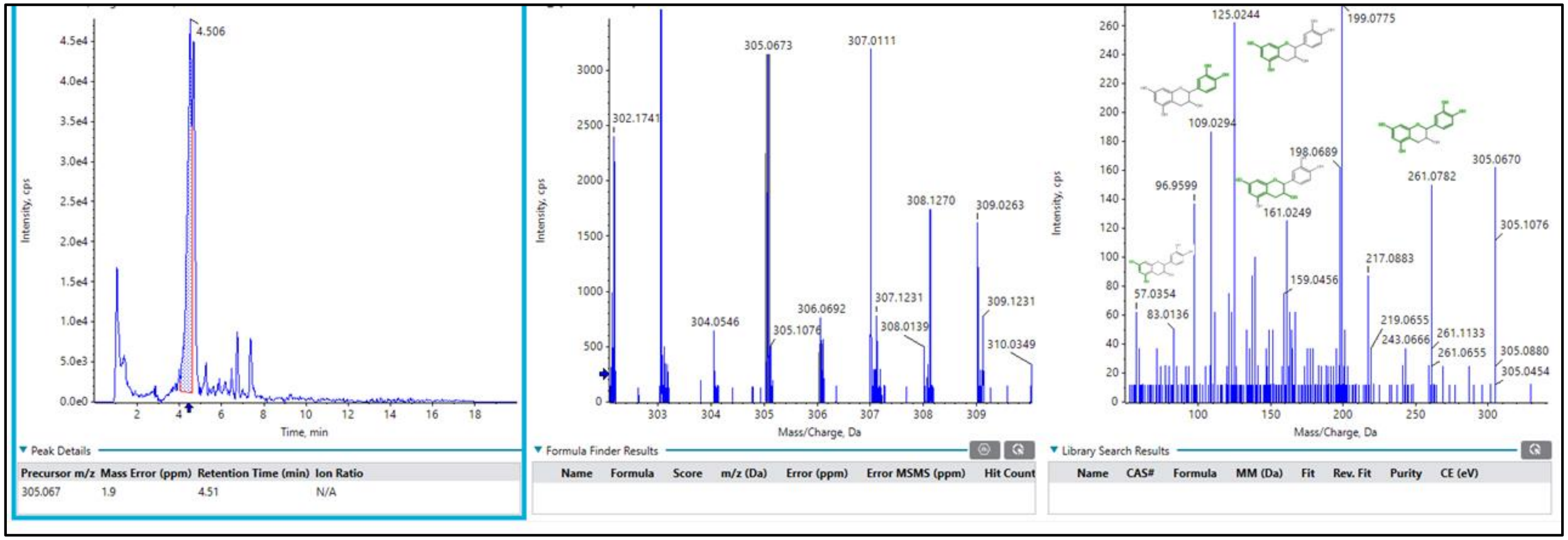


**Figure S8. Extracted ion chromatogram, MS, and MS/MS spectra of epigallocatechin in GSFC.**

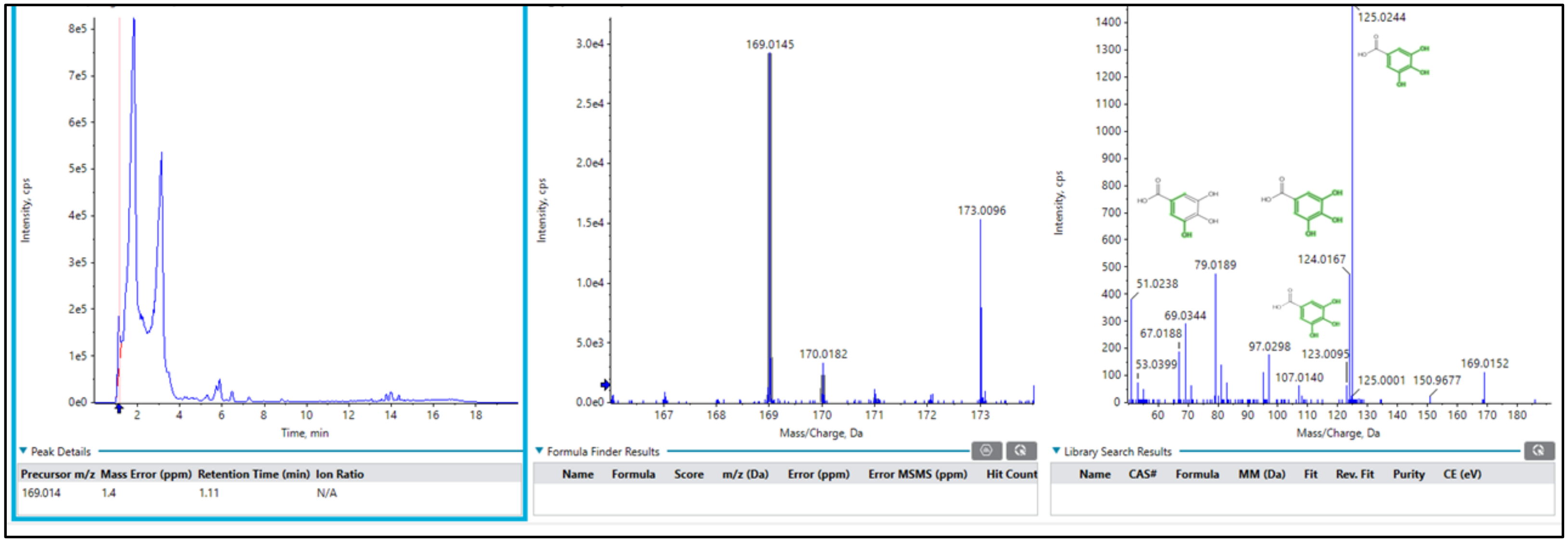


**Figure S9. Extracted ion chromatogram, MS, and MS/MS spectra of gallic acid in GSFC.**

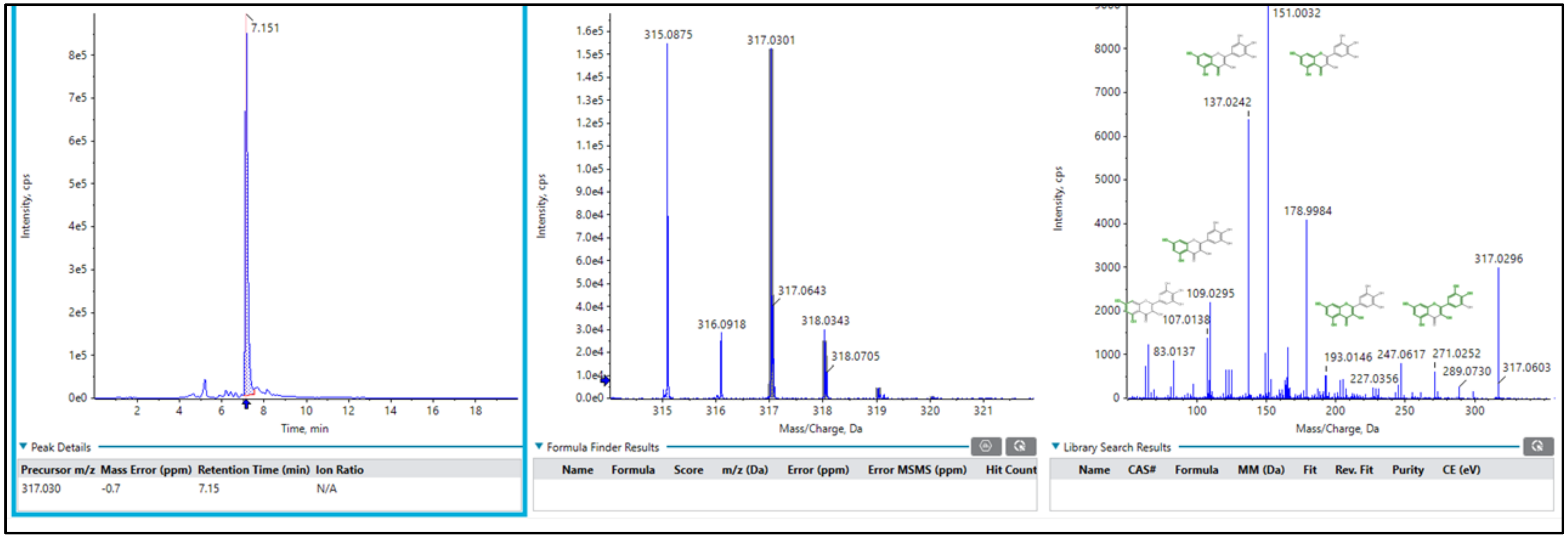


**Figure S10. Extracted ion chromatogram, MS, and MS/MS spectra of myricetin in GFL.**

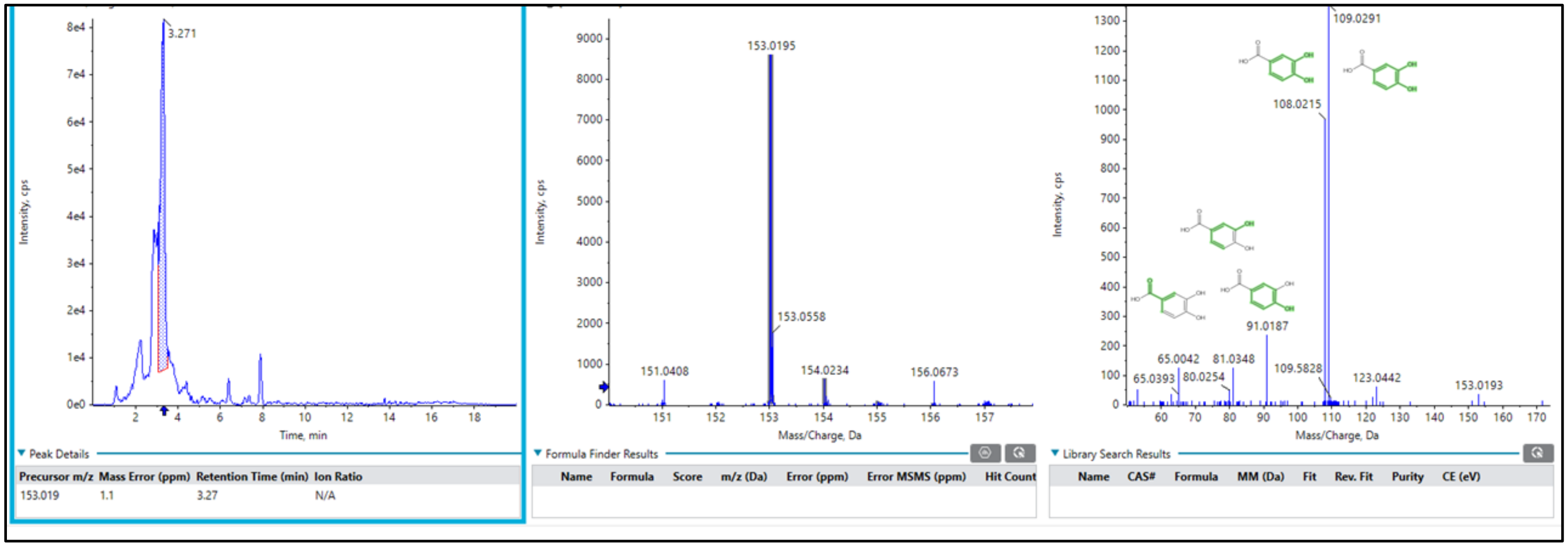


**Figure S11. Extracted ion chromatogram, MS, and MS/MS spectra of procatechuic acid in GSFC.**

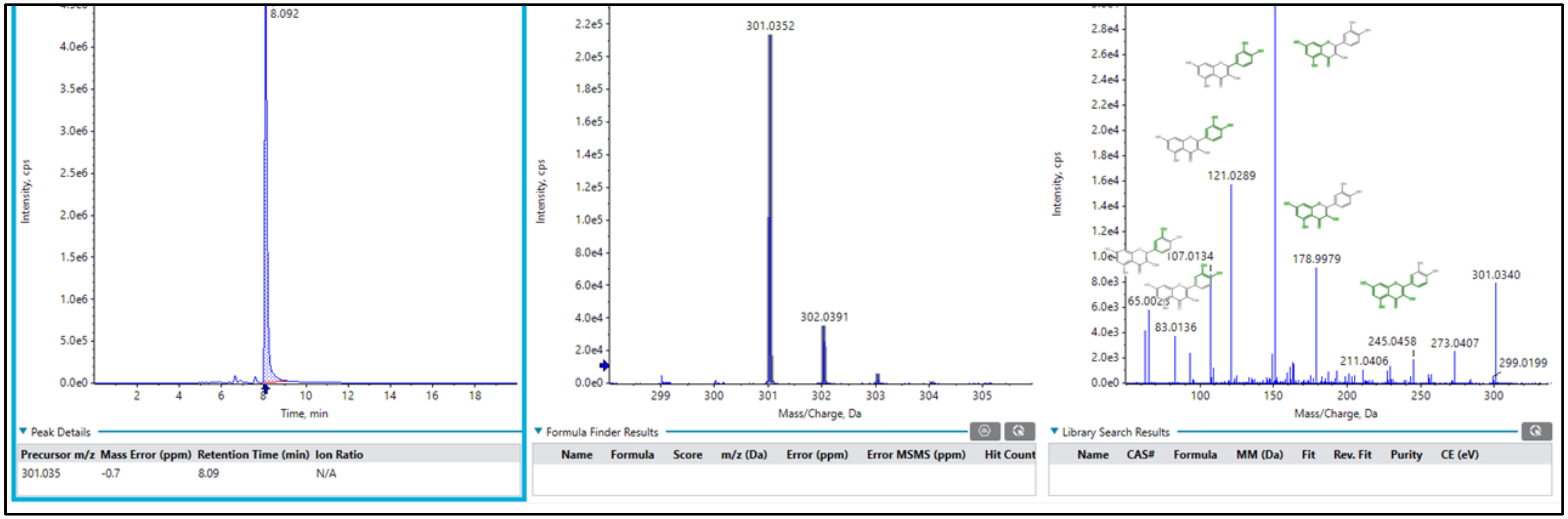


**Figure S12. Extracted ion chromatogram, MS, and MS/MS spectra of quercetin in GSFL.**

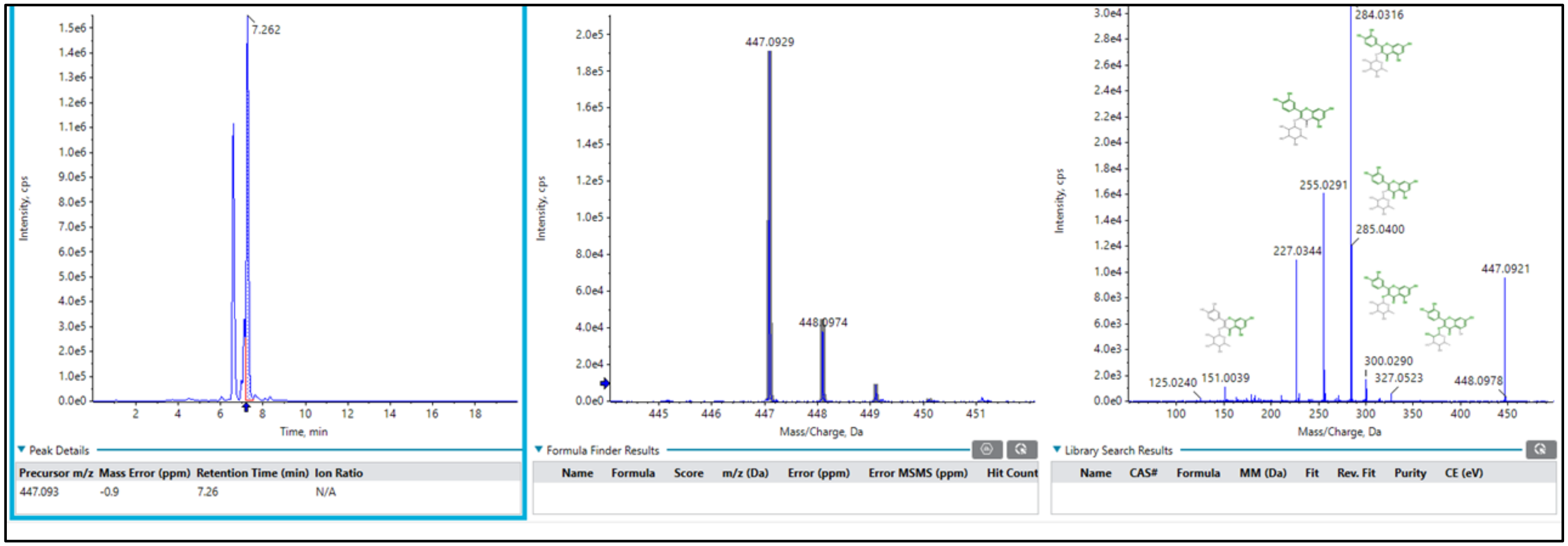


**Figure S13. Extracted ion chromatogram, MS, and MS/MS spectra of quercitrin in GSFC.**

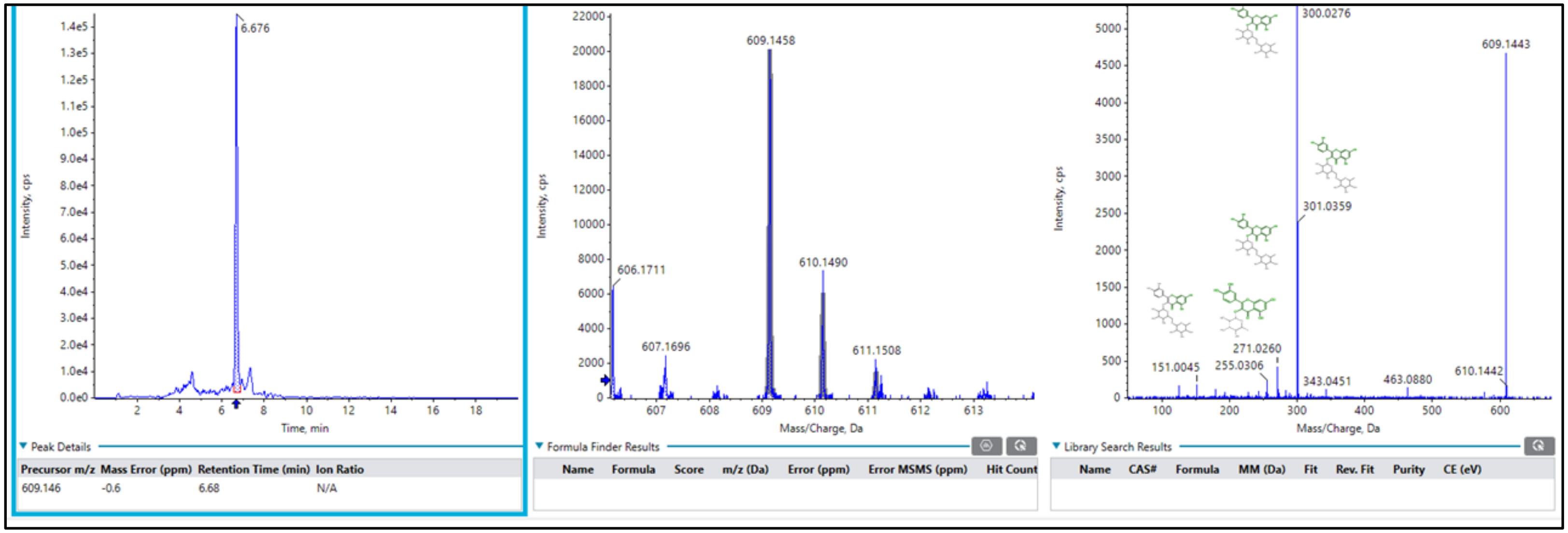


**Figure S14. Extracted ion chromatogram, MS, and MS/MS spectra of rutin in GSFL.**

**Table S4. Suspect screening list for grape seed flour**

| Compound | Molecular formula | Class |
|---|---|---|
| **1-Caffeoyl-β-D-glucose** | $C_{15}H_{18}O_9$ | Steroids and steroid derivatives |
| **1-O-(4-Coumaroyl)-glucose** | $C_{15}H_{18}O_8$ | Cinnamic acids and derivatives |
| **3-Caffeoylshikimic acid** | $C_{16}H_{16}O_8$ | Cinnamic acids and derivatives |
| **Aesculetin** | $C_9H_6O_4$ | Coumarins and derivatives |
| **Aesculin** | $C_{15}H_{16}O_9$ | Coumarins and derivatives |
| **benzoic acid** | $C_7H_6O_2$ | Benzene and substituted derivatives |
| **Caftaric acid** | $C_{13}H_{12}O_9$ | Cinnamic acids and derivatives |
| **Chicoric acid** | $C_{22}H_{18}O_{12}$ | Carboxylic acids and derivatives |
| **Chlorogenic acid** | $C_{16}H_{18}O_9$ | Organooxygen compounds |
| **Cinnamic acid** | $C_9H_8O_2$ | Cinnamic acids and derivatives |
| **Coutaric acid** | $C_{13}H_{12}O_8$ | Cinnamic acids and derivatives |
| **Cyanidin-3-O-glucoside** | $C_{21}H_{21}O_{11}$ | Flavonoids |
| **Delphinidin 3-O-(6''-acetyl-glucoside)** | $C_{23}H_{23}O_{13}$ | Flavonoids |
| **Dihydrofisetin glucoside** | | Flavonoids |
| **Dihydrokaempferol-3-O-rhamnoside** | $C_{21}H_{22}O_{10}$ | Flavonoids |
| **Ellagic acid** | $C_{14}H_6O_8$ | Tannin |
| **Ellagic acid hexoside 1** | $C_{20}H_{16}O_{13}$ | Tannin |
| **Epicatechin gallate** | $C_{22}H_{18}O_{10}$ | Flavonoids |
| **epicatechin-3-O-gallate** | $C_{22}H_{18}O_{10}$ | Flavonoids |
| **Epicatechingallate trimer** | $C_{22}H_{18}O_{10}$ | Flavonoids |
| **Epigallocatechin gallate** | $C_{22}H_{18}O_{11}$ | Flavonoids |
| **Eriodictyol-7-O-glucoside** | $C_{21}H_{22}O_{11}$ | Benzene and substituted derivatives |
| **fertaric acid** | $C_{14}H_{14}O_9$ | Cinnamic acids and derivatives |
| **Fraxin** | $C_{16}H_{18}O_{10}$ | Coumarins and derivatives |
| **Gentistic acid** | $C_7H_6O_4$ | Benzene and substituted derivatives |
| **Hesperetin** | $C_{16}H_{14}O_6$ | Flavonoids |
| **isorhamnetin 3-O-glucoside** | $C_{22}H_{22}O_{12}$ | Flavonoids |
| **kaempferol 3-O-glucoside** | $C_{21}H_{20}O_{11}$ | Flavonoids |
| **Laricitrin 3-O-glucoside** | $C_{22}H_{22}O_{13}$ | Flavonoids |
| **Luteolin-7-O-glucoside** | $C_{21}H_{20}O_{11}$ | Flavonoids |
| **Malvidin 3-(6-acetylglucoside)** | $C_{25}H_{27}O_{13}$ | Flavonoids |
| **Malvidin-3-glucoside** | $C_{23}H_{25}ClO_{12}$ | Flavonoids |
| **Malvidin-3-O-(6-p-coumaroyl)glucoside** | $C_{32}H_{31}O_{14}$ | Flavonoids |
| **Myricetin 3-glucoside** | $C_{21}H_{20}O_{13}$ | Flavonoids |

| | | |
|---|---|---|
| **myricetin-3-O-glucuronide** | $C_{21}H_{18}O_{14}$ | Flavonoids |
| **Petunidin** | $C_{16}H_{13}O_7$ | Flavonoids |
| **Petunidin 3-(6''-acetylglucoside)** | $C_{24}H_{25}O_{13}$ | Flavonoids |
| **p-hydroxybenzoic acid** | $C_7H_6O_3$ | Benzene and substituted derivatives |
| **p-Hydroxybenzoyl glucoside** | $C_{40}H_{45}O_{22}$ | Benzene and substituted derivatives |
| **Procyanidin A1** | $C_{30}H_{24}O_{12}$ | Flavonoids |
| **Procyanidin B1** | $C_{30}H_{26}O_{12}$ | Flavonoids |
| **Procyanidin B2** | $C_{30}H_{26}O_{12}$ | Flavonoids |
| **Procyanidin C** | $C_{45}H_{38}O_{18}$ | Flavonoids |
| **quercetin 3-O-galactoside** | $C_{21}H_{20}O_{12}$ | Flavonoids |
| **quercetin 3-O-glucoside** | $C_{21}H_{20}O_{12}$ | Flavonoids |
| **quercetin 3-O-glucuronide** | $C_{21}H_{18}O_{13}$ | Flavonoids |
| **quercetin 3-O-rhamnoside** | $C_{21}H_{20}O_{11}$ | Flavonoids |
| **Quercetin-3-(3-O-arabinosyl)glucoside** | $C_{26}H_{28}O_{16}$ | Flavonoids |
| **Quercetin-3-O-arabinose** | $C_{20}H_{18}O_{11}$ | Flavonoids |
| **quercetin-3-O-pentosyl-pentoside** | $C_{25}H_{26}O_{15}$ | Flavonoids |
| **Quercetin-3-O-rutinoside** | $C_{27}H_{30}O_{16}$ | Flavonoids |
| **Quercetin-3-rutinoside** | $C_{27}H_{30}O_{16}$ | Flavonoids |
| **Quinic acid** | $C_7H_{12}O_6$ | Organooxygen compounds |
| **isorhamnetin-3-O-rutinoside** | $C_{28}H_{32}O_{16}$ | Flavonoids |
| **Taxifolin-3-O-glucoside** | $C_{21}H_{22}O_{12}$ | Flavonoids |
| **Taxifolin-3-O-rhamnoside** | $C_{21}H_{22}O_{11}$ | Flavonoids |
| **Taxifolin-O-pentoside** | $C_{20}H_{20}O_{11}$ | Flavonoids |
| **trans-Piceatannol** | $C_{14}H_{12}O_4$ | Stilbenes |
| **trans-Piceid** | $C_{20}H_{22}O_8$ | Stilbenes |
| **trans-polydatin** | $C_{20}H_{22}O_8$ | Stilbenes |
| **trans-resveratrol** | $C_{14}H_{12}O_3$ | Stilbenes |
| **Trifolin** | $C_{21}H_{20}O_{11}$ | Flavonoids |
| **Umbelliferone** | $C_9H_6O_3$ | Coumarins and derivatives |
| **Vitisin A** | $C_{56}H_{42}O_{12}$ | Flavonoids |

**Table S5. Suspect screening results for commercial grape seed flour (GSFC) and grape seed flour from Lemnos (GSFL)**

| Compound | Molecular Formula | $[M-H]^-$ experimental GSFC | $[M-H]^-$ experimental GSFL | Rt (min) | Fragments m/z | Elemental Formula | GSFC mg/kg | GS FL mg/kg | Mass Bank ID |
|---|---|---|---|---|---|---|---|---|---|
| **3-Caffeoylshikimic acid** | $C_{16}H_{16}O_8$ | ND | 335.0799 | 5.92 | 112.0057<br>116.0510<br>130.155<br>157.0775<br>255.0784<br>299.0856 | | **ND** | **0.504** | |
| **Caftaric acid** | $C_{13}H_{12}O_9$ | ND | 311.0413 | 3.86 | 87.0089<br>134.0375<br>135.0454<br>149.0097<br>179.0348 | $[C_3H_4O_3]$-$H^-$<br>$[C_8H_7O_2]$-$H^-$<br>$[C_8H_7O_2]^-$<br>$[C_4H_5O_6]^-$<br>$[C_9H_7O_4]^-$ | **ND** | **10.7** | |
| **Chicoric acid** | $C_{22}H_{18}O_{12}$ | ND | 473.0726 | 3.81 | 137.0239<br>125.0241<br>429.0835 | $[C_6H_4O_3+H]^-$<br>$[C_7H_5O_3]^-$<br>$[C_{21}H_{17}O_{10}]^-$ | **ND** | **5.80** | |
| **Coutaric acid** | $C_{13}H_{12}O_8$ | 295.0464 | 295.0462 | 4.56 | 59.0138<br>87.0088<br>119.0498<br>163.0393 | $[C_2H_2O_2+H]^-$<br>$[C_3H_4O_3]$-$H^-$<br>$[C_8H_7O]^-$<br>$[C_9H_7O_3]^-$ | **try** | **87.7** | |
| **Cyanidin-3-O-glucoside** | $C_{21}H_{21}O_{11}$ | 448.0983 | 448.0978 | 6.58 | 125.0240<br>284.0336<br>285.0388 | $[C_6H_4O_3+H]^-$<br>$[C_{15}H_{10}O_6$-$H]$-$H^-$<br>$[C_{15}H_{10}O_6]$-$H^-$ | **2.99** | **2.67** | PR305979 |
| **Dihydrokaempferol-3-O-rhamnoside** | $C_{21}H_{22}O_{10}$ | 433.1153 | 433.1149 | 6.21 | 107.0143<br>119.0501<br>151.0038 | $[C_6H_4O_2]$-$H^-$<br>$[C_8H_5O+2H]^-$<br>$[C_7H_4O_4]$-$H^-$ | **0.581** | **0.365** | |
| **Ellagic acid** | $C_{14}H_6O_8$ | 300.9989 | 300.9990 | 6.86 | 201.0193<br>229.0141<br>283.9969 | $[C_{11}H_4O_4+H]^-$<br>$[C_{12}H_6O_5]$-$H^-$<br>$[C_{14}H_5O_7]$-$H^-$ | **1280.7** | **184.1** | |
| **Ellagic acid hexoside 1** | $C_{20}H_{16}O_{13}$ | 463.0518 | 463.0536 | 5.78 | 271.9974<br>300.9979<br>299.9899 | $[C_{13}H_5O_7]$-$H^-$<br>$[C_{14}H_5O_8]$-$H^-$<br>$[C_{14}H_5O_8]^-$ | **213.7** | **13.41** | |
| **epicatechin-3-O-gallate / trimer** | $C_{22}H_{18}O_{10}$ | 441.0824 | 441.0819 | 5.73 | 97.0291<br>109.0292<br>124.0161<br>125.0233<br>169.0127 | $[C_5H_5O_2]^-$<br>$[C_6H_5O_2]^-$<br>$[C_6H_5O_3]$-$H^-$<br>$[C_6H_5O_3]^-$<br>$[C_7H_5O_5]^-$ | **30.41** | **153.9** | |
| **Epigallocatechin gallate** | $C_{22}H_{18}O_{11}$ | 457.0781 | 457.0775 | 4.65 | 95.0142<br>125.0247<br>123.0089<br>166.9990<br>203.0722<br>289.0721 | $[C_5H_4O_2]$-$H^-$<br>$[C_6H_5O_3$-$H]$-$H^-$<br>$[C_6H_5O_3]^-$<br>$[C_8H_5O_4]^-$<br>$[C_7H_5O_5$-$H]$-$H^-$<br>$[C_{15}H_{13}O_6]^-$ | **0.348** | **1.76** | BS003900 |

| | | | | | | | | | |
|---|---|---|---|---|---|---|---|---|---|
| **Eriodictyol-7-O-glucoside** | $C_{21}H_{22}O_{11}$ | 449.1089 | 449.1085 | 5.30 | 125.0239<br>178.9986<br>243.0665<br>259.0602<br>269.0448 | $[C_6H_3O_3+2H]^-$<br>$[C_8H_4O_5]-H^-$<br>$[C_{14}H_{11}O_4]^-$<br>$[C_{14}H_{11}O_5]^-$<br>$[C_{15}H_{11}O_5-H]-H^-$ | **127.3** | **200.3** | PR306200 |
| **Fertaric acid** | $C_{14}H_{14}O_9$ | 325.0572 | 325.0571 | 4.91 | 59.0136<br>87.0082<br>119.0501<br>134.0372<br>149.0610<br>193.0506 | $[C_2H_2O_2+H]^-$<br>$[C_3H_4O_3]-H^-$<br>$[C_8H_6O+H]^-$<br>$[C_8H_6O_2]^-$<br>$[C_9H_9O_2]^-$<br>$[C_{10}H_9O_4]^-$ | **17.3** | **20.7** | |
| **Fraxin** | $C_{16}H_{18}O_{10}$ | ND | 369.0833 | 5.09 | 135.0075<br>163.0040<br>190.9994<br>192.0070<br>206.0232<br>345.0837 | $[C_7H_5O_3-H]-H^-$<br>$[C_8H_5O_4-H]-H^-$<br>$[C_9H_4O_5]-H^-$<br>$[C_9H_4O_5]^-$<br>$[C_{10}H_7O_5]-H^-$<br>$[C_{14}H_{16}O_{10}+H]^-$ | **ND** | **29.9** | |
| **Gallocatechin** | $C_{15}H_{14}O_7$ | ND | 305.0670 | 4.68 | 57.0343<br>109.0294<br>125.0240<br>161.0245<br>195.0297 | $[C_3H_5O]^-$<br>$[C_6H_4O_2+H]^-$<br>$[C_6H_5O_3]^-$<br>$[C_9H_8O_3-2H]-H^-$<br>$[C_9H_8O_5]-H^-$ | **ND** | **14.2** | BS003902 |
| **isorhamnetin 3-O-glucoside** | $C_{22}H_{22}O_{12}$ | 477.1045 | 477.1039 | 7.31 | 125.0239<br>151.0042<br>242.0222<br>243.0309<br>314.0438<br>357.0615 | $[C_6H_4O_3+H]^-$<br>$[C_7H_4O_4]-H^-$<br>$[C_{13}H_8O_5-H]-H^-$<br>$[C_{13}H_8O_5]-H^-$<br>$[C_{16}H_{11}O_7]-H^-$<br>$[C_{18}H_{14}O_8]-H^-$ | **0.568** | **1.16** | PR306618 |
| **isorhamnetin-3-O-rutinoside** | $C_{28}H_{32}O_{16}$ | ND | 623.1630 | 7.34 | 299.0206<br>314.0437<br>315.0520 | $[C_{15}H_8O_7]-H^-$<br>$[C_{16}H_{11}O_7]-H^-$<br>$[C_{16}H_{11}O_7]^-$ | **ND** | **0.057** | PR305460 |
| **kaempferol 3-O-glucoside** | $C_{21}H_{20}O_{11}$ | 447.0933 | 447.0930 | 7.24 | 178.9998<br>227.0349<br>284.0324<br>285.0405 | $[C_8H_4O_5]-H^-$<br>$[C_{13}H_9O_4-H]-H^-$<br>$[C_{15}H_9O_6]-H^-$<br>$[C_{15}H_9O_6]^-$ | **1.84** | **1.75** | BS003478 |
| **Laricitrin 3-O-glucoside** | $C_{22}H_{22}O_{13}$ | ND | 493.0995 | 6.75 | 178.9985<br>271.0259<br>287.0198<br>315.0148 | $[C_8H_4O_5]-H^-$<br>$[C_{14}H_8O_6]-H^-$<br>$[C_{14}H_8O_7]-H^-$<br>$[C_{15}H_8O_8]-H^-$ | **ND** | **0.461** | |
| **Luteolin-7-O-glucoside** | $C_{21}H_{20}O_{11}$ | 447.0931 | 447.0929 | 6.59 | 199.0407<br>284.0322<br>285.0396 | $[C_{12}H_6O_3+H]^-$<br>$[C_{15}H_9O_6]-H^-$<br>$[C_{15}H_9O_6]^-$ | **2.19** | **1.94** | PR305631 |
| **Myricetin 3-glucoside** | $C_{21}H_{20}O_{13}$ | 479.0843 | 479.0831 | 6.14 | 151.0037<br>270.0166<br>287.0196<br>271.0241<br>316.0206 | $[C_7H_4O_4]-H^-$<br>$[C_{14}H_9O_6-2H]-H^-$<br>$[C_{14}H_9O_6-H]-H^-$<br>$[C_{14}H_9O_7-H]-H^-$<br>$[C_{15}H_9O_8]-H^-$ | **0.218** | **5.19** | |
| **myricetin-3-O-glucuronide** | $C_{21}H_{18}O_{14}$ | 493.0636 | 493.0629 | 6.04 | 125.0249<br>137.0243<br>151.0040<br>178.9989 | $[C_6H_5O_3]^-$<br>$[C_7H_4O_3+H]^-$<br>$[C_7H_4O_4]-H^-$<br>$[C_8H_5O_5-H]-H^-$ | **0.066** | **0.797** | |

| | | | | | | | | | |
|---|---|---|---|---|---|---|---|---|---|
| **p-hydroxybenzoic acid** | $C_7H_6O_3$ | 137.0246 | 137.0245 | 4.24 | 65.0386<br>81.0347<br>91.0191<br>92.0265<br>108.0213 | $[C_5H_4+H]^-$<br>$[C_5H_5O]^-$<br>$[C_6H_5O-H]-H^-$<br>$[C_6H_5O]-H^-$<br>$[C_6H_5O_2]-H^-$ | **56.1** | **77.4** | |
| **Procyanidin A1** | $C_{30}H_{24}O_{12}$ | 575.1195 | 575.1196 | 6.57 | 125.0246<br>137.0247<br>243.0309<br>271.0255<br>287.0574<br>394.0703 | $[C_6H_4O_3+H]^-$<br>$[C_7H_6O_3]-H^-$<br>$[C_{13}H_9O_5-H]-H^-$<br>$[C_{14}H_9O_6-H]-H^-$<br>$[C_{15}H_{12}O_6]-H^-$<br>$[C_{21}H_{14}O_8]^-$ | **2.37** | **11.0** | |
| **Procyanidin B1/B2** | $C_{30}H_{26}O_{12}$ | 577.1340 | 577.1336 | 4.08 | 125.0230<br>161.0234<br>289.0696<br>407.0746<br>425.0855 | $[C_6H_4O_3+H]^-$<br>$[C_9H_8O_3-2H]-H^-$<br>$[C_{15}H_{13}O_6]^-$<br>$[C_{22}H_{17}O_8-H]-H^-$<br>$[C_{22}H_{18}O_9]-H^-$ | **156.6** | **259.0** | PR308824 |
| **quercetin 3-O-galactoside/ quercetin 3-O-glucoside** | $C_{21}H_{20}O_{12}$ | 463.0883 | 463.0879 | 6.71 | 151.0037<br>245.0459<br>271.0241<br>300.0262 | $[C_7H_4O_4]-H^-$<br>$[C_{13}H_9O_5]^-$<br>$[C_{14}H_9O_6-H]-H^-$<br>$[C_{15}H_9O_7]-H^-$ | **2.52** | **4.79** | PR309229 |
| **quercetin 3-O-glucuronide** | $C_{21}H_{18}O_{13}$ | 477.0670 | 477.0668 | 6.64 | 121.0294<br>163.0036<br>151.0027<br>178.9978 | $[C_7H_5O_2]^-$<br>$[C_7H_4O_4]-H^-$<br>$[C_8H_5O_4-H]-H^-$<br>$[C_8H_4O_5]-H^-$ | **6.71** | **10.7** | PR100978 |
| **quercetin 3-O-rhamnoside** | $C_{21}H_{20}O_{11}$ | 447.0931 | 447.0931 | 6.57 | 284.0318<br>285.0392 | $[C_{15}H_9O_6]-H^-$<br>$[C_{15}H_9O_6]^-$ | **2.55** | **2.33** | PR305653 |
| **Quercetin-3-O-rutinoside** | $C_{27}H_{30}O_{16}$ | 609.1466 | 609.1457 | 6.66 | 151.0042<br>271.0260<br>300.0285<br>301.0363 | $[C_7H_4O_4]-H^-$<br>$[C_{14}H_9O_6-H]-H^-$<br>$[C_{15}H_9O_7]-H^-$<br>$[C_{15}H_9O_7]^-$ | **0.181** | **0.339** | PR306381 |
| **Taxifolin-3-O-glucoside** | $C_{21}H_{22}O_{12}$ | 465.1047 | 465.1041 | 4.63 | 125.0242<br>177.0198<br>217.0508<br>275.0565 | $[C_6H_4O_3+H]^-$<br>$[C_9H_6O_4]-H^-$<br>$[C_{12}H_8O_4+H]^-$<br>$[C_{14}H_{11}O_6]^-$ | **0.563** | **0.700** | |
| **Taxifolin-3-O-rhamnoside** | $C_{21}H_{22}O_{11}$ | 449.1093 | 449.1087 | 5.28 | 125.0238<br>178.9983<br>269.0443 | $[C_6H_4O_3+H]^-$<br>$[C_8H_5O_5-H]-H^-$<br>$[C_{15}H_{10}O_5]-H^-$ | **3.85** | **5.89** | |
| **trans-Piceatannol** | $C_{14}H_{12}O_4$ | ND | 243.0667 | 8.75 | 93.0348<br>117.0333<br>172.0516<br>171.0454 | $[C_6H_4O+H]^-$<br>$[C_8H_6O]-H^-$<br>$[C_{11}H_9O_2-H]-H^-$<br>$[C_{11}H_9O_2]-H^-$ | **ND** | **0.489** | |
| **trans-Piceid/ trans-polydatin** | $C_{20}H_{22}O_8$ | 389.1251 | 389.1248 | 7.07 | 143.0502<br>185.0611<br>227.0708 | $[C_7H_{11}N_3OS]^-$ | **0.729** | **3.62** | PR308288 |
| **trans-resveratrol** | $C_{14}H_{12}O_3$ | ND | 227.0717 | 7.08 | 143.0508<br>183.0821<br>185.0689<br>227.0720 | $[C_{10}H_8O]-H^-$<br>$[C_{13}H_{10}O+H]^-$ | **ND** | **1.01** | |
| **Trifolin** | $C_{21}H_{20}O_{11}$ | 447.0935 | 447.0929 | 6.59 | 199.0403<br>284.0318<br>285.0392 | $[C_{12}H_6O_3+H]^-$<br>$[C_{15}H_9O_6]-H^-$<br>$[C_{15}H_9O_6]^-$ | **13.7** | **12.1** | |

**Table S6. Target screening results for commercial olive stone flour (OSFC) and olive stone flour from Lemnos (OSFL)**

| Compound | Molecular Formula | $[M-H]^-$ standard | $[M-H]^-$ experimental OSFC | $[M-H]^-$ experimental OSFL | Rt (min) | ΔRt | Fragments m/z | Elemental Formula | OSFC ±SD mg/kg | OSFL ±SD mg/kg |
|---|---|---|---|---|---|---|---|---|---|---|
| **Apigenin** | $C_{15}H_{10}O_5$ | 269.0448 | 269.0460 | 269.0452 | 9.04 | 0 | 63.0238<br>65.0027<br>107.0133<br>117.0335<br>149.0237<br>159.0448<br>225.0553 | $[C_5H_4]-H^-$<br>$[C_4H_3O-H]-H^-$<br>$[C_6H_4O_2]-H^-$<br>$[C_8H_6O]-H^-$<br>$[C_8H_5O_3]^-$<br>$[C_{10}H_7O_2]^-$<br>$[C_{14}H_9O_3]^-$ | **0.024 ±0.001** | **5.05 ±0.120** |
| **Caffeic acid** | $C_9H_8O_4$ | 179.0348 | 179.0353 | 179.0351 | 5.12 | -0.01 | 65.0404<br>79.0552<br>134.0375<br>135.0448 | $[C_4H_2O]-H^-$<br>$[C_6H_5+2H]^-$<br>$[C_8H_7O_2]-H^-$<br>$[C_8H_7O_2]^-$ | **0.111 ±0.05** | **1.08 ±0.036** |
| **Coumaric acid** | $C_9H_8O_3$ | 163.0400 | 163.0403 | 163.0399 | 5.97 | -0.02 | 65.0394<br>93.0351<br>117.0349<br>119.0501 | $[C_5H_4+H]^-$<br>$[C_6H_5O]^-$<br>$[C_8H_7O-H]-H^-$<br>$[C_8H_7O]^-$ | **1.75 ±0.09** | **1.33 ±0.04** |
| **Chrysin** | $C_{15}H_{10}O_4$ | 253.0510 | 253.0510 | 253.0506 | 10.54 | +0.01 | 63.0237<br>57.0326<br>77.0389<br>107.0144<br>143.0497<br>145.0293<br>181.0624<br>191.1444<br>209.1551<br>253.0512 | $[C_5H_5-H]-H^-$<br>$[C_6H_4O_2]-H^-$<br>$[C_{10}H_7O]^-$<br>$[C_{14}H_9O_2]^-$ | | |
| **Diosmin** | $C_{28}H_{32}O_{15}$ | 607.1669 | ND | 607.1664 | 7.17 | +0.05 | 151.0397<br>199.0616<br>283.0266<br>284.0331 | $[C_8H_7O_3]^-$<br>$[C_9H_{12}O_5]-H^-$<br>$[C_{15}H_8O_6]-H^-$<br>$[C_{15}H_8O_6]^-$ | **ND** | **6.13 ±0.153** |

| | | | | | | | | | | |
|---|---|---|---|---|---|---|---|---|---|---|
| | | | | | | | 443.0990 | $[C_{22}H_{21}O_{10}-H]-H^-$ | | |
| **Ferulic acid** | $C_{10}H_{10}O_4$ | 193.0505 | 193.0513 | 193.0508 | 6.25 | -0.03 | 106.0414<br>132.0210<br>133.0301<br>134.0375<br>178.0276 | $[C_7H_6O]^-$<br>$[C_8H_6O_2-H]-H^-$<br>$[C_8H_6O_2]-H^-$<br>$[C_8H_6O_2]^-$<br>$[C_9H_7O_4]-H^-$ | **2.36 ±0.145** | **5.93 ±0.178** |
| **Gallic acid** | $C_7H_6O_5$ | 169.0141 | ND | 169.0144 | 1.04 | +0.07 | 69.0344<br>123.0088<br>124.0168<br>125.0243 | $[C_4H_3O+2H]^-$<br>$[C_6H_5O_3-H]-H^-$<br>$[C_6H_5O_3]-H^-$<br>$[C_6H_5O_3]^-$ | **0.294 ±0.118** | **13.7 ±1.169** |
| **Luteolin** | $C_{15}H_{10}O_6$ | 285.0401 | 285.0411 | 285.0395 | 8.36 | -0.01 | 65.0036<br>107.0138<br>132.0221<br>151.0038<br>199.0402<br>217.0515<br>241.0504 | $[C_4H_3O-H]-H^-$<br>$[C_6H_5O_2-H]-H^-$<br>$[C_8H_6O_2-H]-H^-$<br>$[C_7H_4O_4]-H^-$<br>$[C_{12}H_6O_3+H]^-$<br>$[C_{12}H_9O_4]^-$<br>$[C_{14}H_9O_4]^-$ | **1.02 ±0.036** | **28.3 ±0.957** |
| **Quercetin** | $C_{15}H_{10}O_7$ | 301.0350 | 301.0370 | 301.0354 | 8.08 | +0.01 | 65.0029<br>83.0137<br>107.0135<br>121.0292<br>151.0034<br>178.9982<br>186.0319 | $[C_4H_3O-H]-H^-$<br>$[C_4H_4O_2]-H^-$<br>$[C_6H_5O_2-H]-H^-$<br>$[C_7H_5O_2]^-$<br>$[C_7H_4O_4]-H^-$<br>$[C_8H_5O_5-H]-H^-$<br>$[C_{11}H_7O_3]-H^-$ | **0.015 ±0.001** | **0.587 ±0.042** |
| **Quercitrin** | $C_{21}H_{20}O_{11}$ | 447.0930 | 447.0938 | 447.0929 | 7.19 | +0.01 | 151.0040<br>255.0301<br>284.0326<br>285.0409<br>300.0282<br>301.0367<br>327.0529 | $[C_7H_4O_4]-H^-$<br>$[C_{14}H_9O_5-H]-H^-$<br>$[C_{15}H_9O_6]-H^-$<br>$[C_{15}H_9O_6]^-$<br>$[C_{15}H_9O_7]-H^-$<br>$[C_{15}H_9O_7]^-$<br>$[C_{17}H_{11}O_7]^-$ | **0.062 ±0.014** | **8.22 ±0.305** |
| **Procatechuic acid** | $C_7H_6O_4$ | 153.0192 | 153.0195 | 153.0193 | 3.26 | +0.05 | 65.0032<br>81.0343<br>91.0187 | $[C_4H_2O]-H^-$<br>$[C_5H_4O+H]^-$<br>$[C_6H_4O]-H^-$ | **3.96 ±0.606** | **95.7 ±0.984** |

| | | | | | | | | | | |
|---|---|---|---|---|---|---|---|---|---|---|
| | | | | | | | 108.0210<br>109.0287 | $[C_6H_5O_2]-H^-$<br>$[C_6H_5O_2]^-$ | | |
| **Rutin (Quercetin rutinoside)** | $C_{27}H_{30}O_{16}$ | 609.1456 | 609.1466 | 609.1459 | 6.67 | +0.01 | 151.0041<br>255.0300<br>271.0250<br>300.0266<br>301.0350 | $[C_7H_4O_4]-H^-$<br>$[C_{14}H_9O_5-H]-H^-$<br>$[C_{14}H_9O_6-H]-H^-$<br>$[C_{15}H_9O_7]-H^-$<br>$[C_{15}H_9O_7]^-$ | **1.38 ±0.1275** | **10.3 ±0.597** |
| **Sinapic acid** | $C_{11}H_{12}O_5$ | 223.0611 | 223.0616 | 223.0612 | 6.26 | | 67.0188<br>68.9981<br>69.0345<br>93.0351<br>121.0307<br>127.0412<br>149.0253<br>181.0492<br>193.0159 | $[C_4H_4O]-H^-$<br>$[C_3H_3O_2-H]-H^-$<br>$[C_4H_4O+H]^-$<br>$[C_6H_6O]-H^-$<br>$[C_7H_6O_2]-H^-$<br>$[C_6H_8O_3]-H^-$<br>$[C_8H_7O_3-H]-H^-$<br>$[C_9H_6O_5]-H^-$ | **7.53 ±0.201** | **1.18 ±0.096** |
| **Taxifolin** | $C_{15}H_{12}O_7$ | 303.0508 | ND | 303.0512 | 6.09 | +0.01 | 57.0339<br>83.0142<br>121.0294<br>123.0450<br>125.0244<br>175.0409<br>217.0504<br>285.0417 | $[C_3H_3O+2H]^-$<br>$[C_4H_4O_2]-H^-$<br>$[C_7H_6O_2]-H^-$<br>$[C_7H_6O_2+H]^-$<br>$[C_6H_4O_3+H]^-$<br>$[C_{10}H_7O_3]^-$<br>$[C_{12}H_8O_4+H]^-$<br>$[C_{15}H_{11}O_6-H]-H^-$ | **ND** | **0.336 ±0.071** |
| **Vanillic acid** | $C_8H_8O_4$ | 167.0351 | 167.0351 | 167.0353 | 5.13 | -0.04 | 65.0034<br>91.0189<br>108.0217<br>123.0094<br>124.0167<br>152.0117 | $[C_4H_2O]-H^-$<br>$[C_6H_4O]-H^-$<br>$[C_6H_4O_2]^-$<br>$[C_6H_5O_3-H]-H^-$<br>$[C_6H_5O_3]-H^-$<br>$[C_7H_5O_4]-H^-$ | **8.82 ±0.498** | **6.00 ±0.209** |
| **Vanillin** | $C_8H_8O_3$ | 151.0400 | 151.0402 | 151.0401 | 5.64 | +0.03 | 51.0236<br>65.0031<br>92.0269<br>93.0344<br>108.0214 | $[C_4H_3]^-$<br>$[C_4H_3O-H]-H^-$<br>$[C_6H_4O]^-$<br>$[C_6H_4O+H]^-$ | **7.55 ±0.927** | **89.8 ±1.074** |

| | | | | | | | | | | |
|---|---|---|---|---|---|---|---|---|---|---|
| | | | | | | | 122.0370<br>136.0164<br>151.0414 | $[C_6H_4O_2]^-$<br>$[C_7H_7O_2]$-$H^-$<br>$[C_7H_5O_3]$-$H^-$ | | |

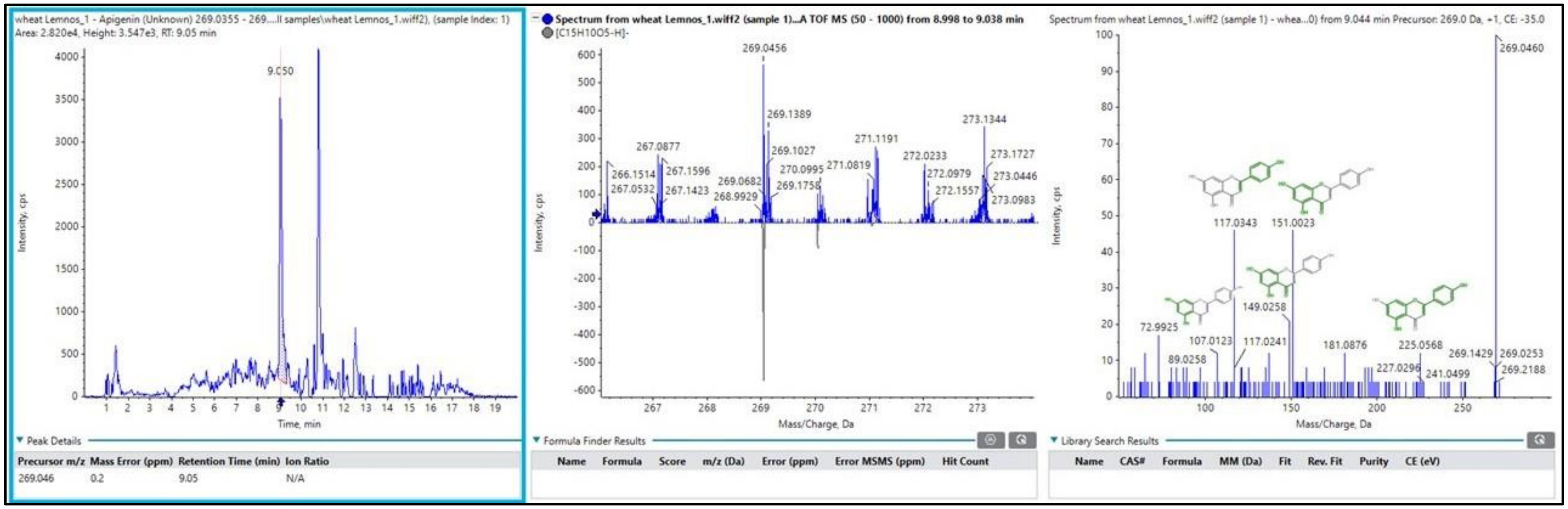


**Figure S15. Extracted ion chromatogram, MS, and MS/MS spectra of apigenin in OSFL.**

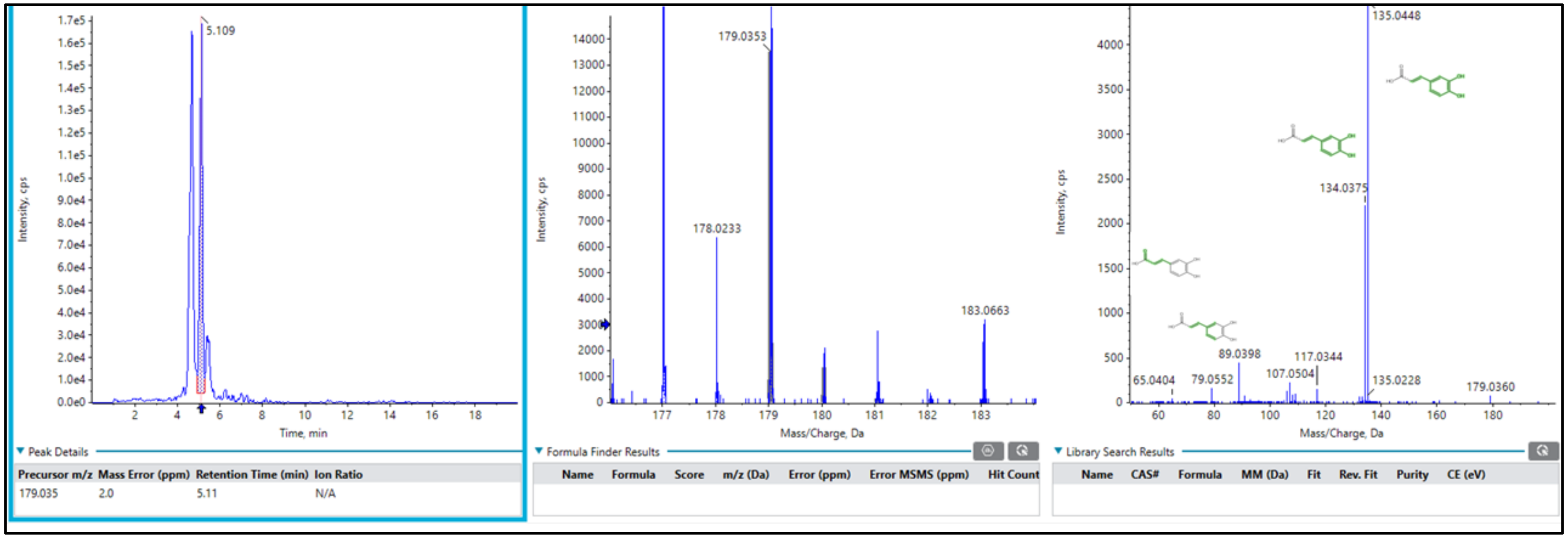


**Figure S16. Extracted ion chromatogram, MS, and MS/MS spectra of caffeic acid in OSFC.**

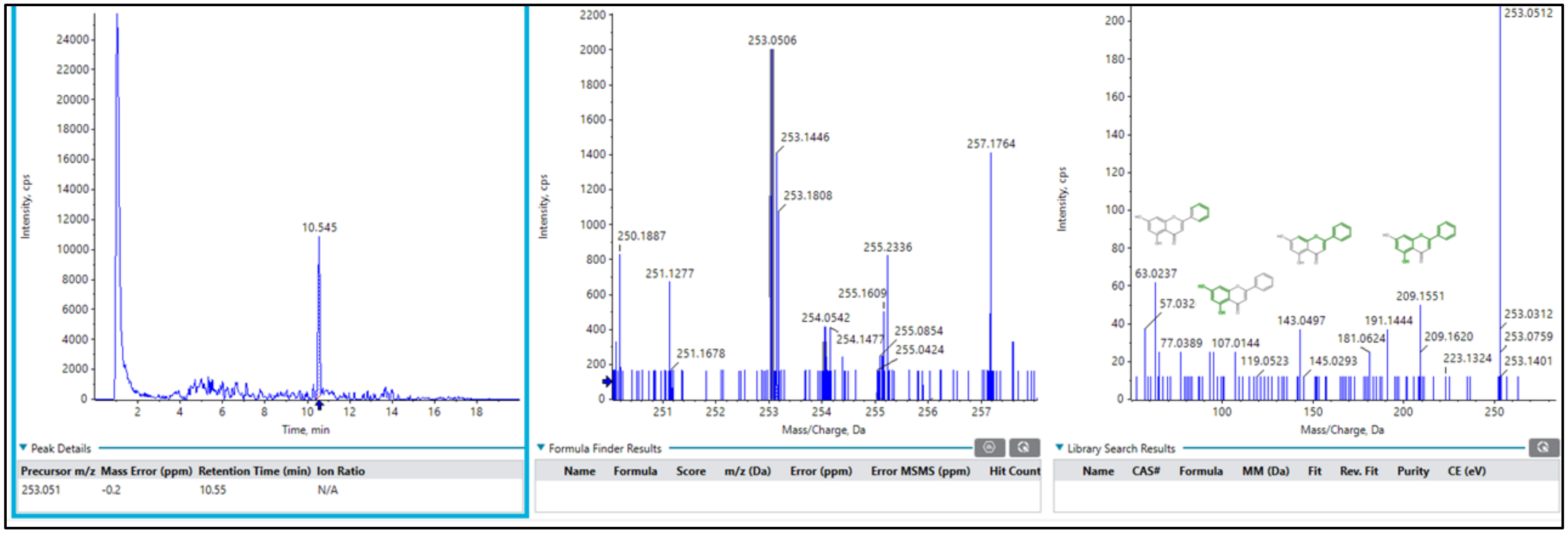


**Figure S17. Extracted ion chromatogram, MS, and MS/MS spectra of chrysin in OSFC.**

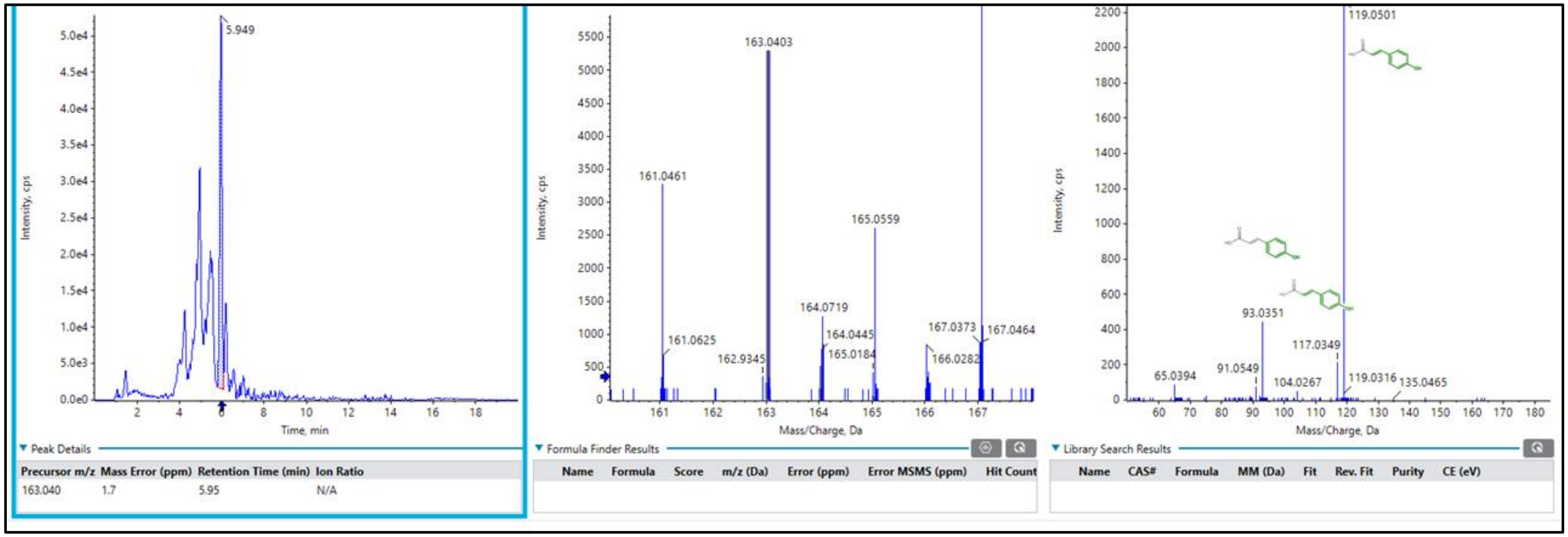


**Figure S18. Extracted ion chromatogram, MS, and MS/MS spectra of coumaric acid in OSFC.**

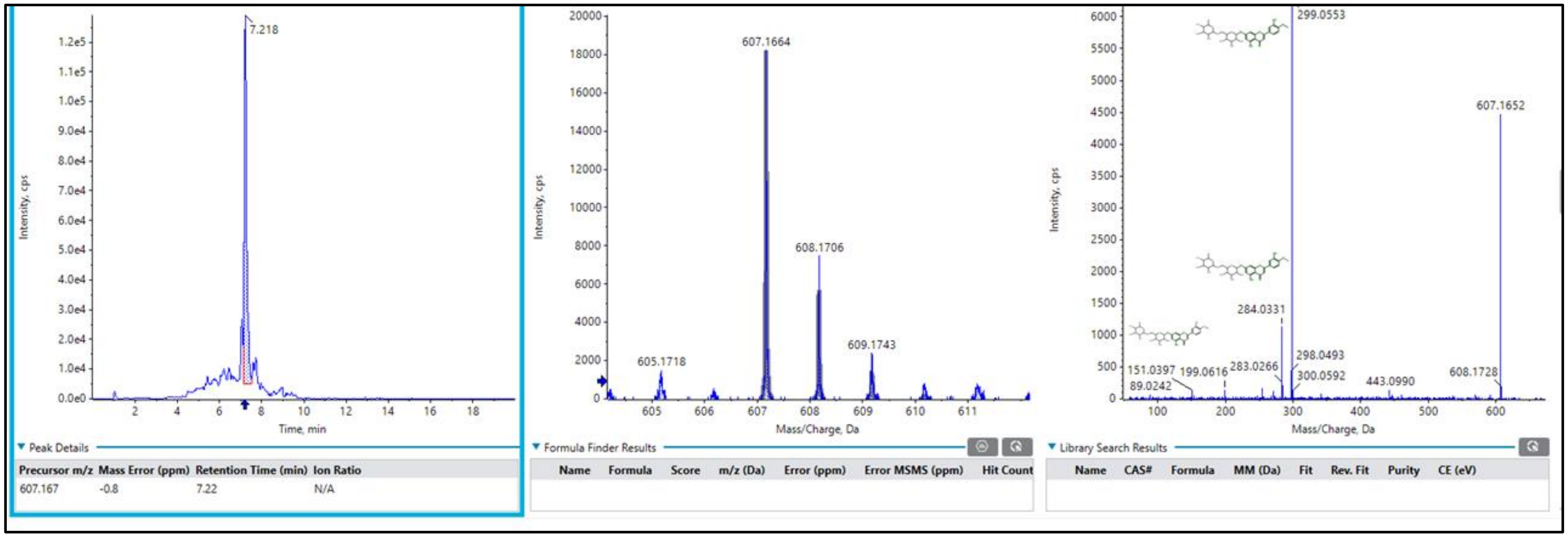


**Figure S19. Extracted ion chromatogram, MS, and MS/MS spectra of diosmin in OSFL.**

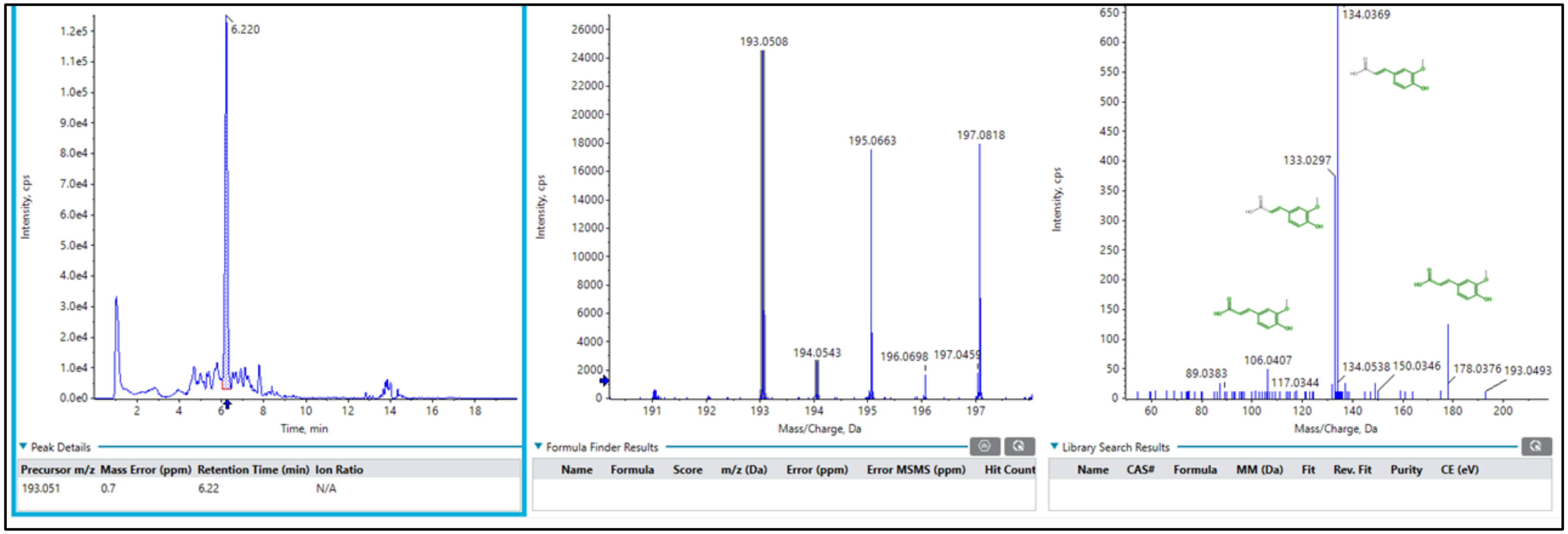


**Figure S20. Extracted ion chromatogram, MS, and MS/MS spectra of ferulic acid in OSFL.**

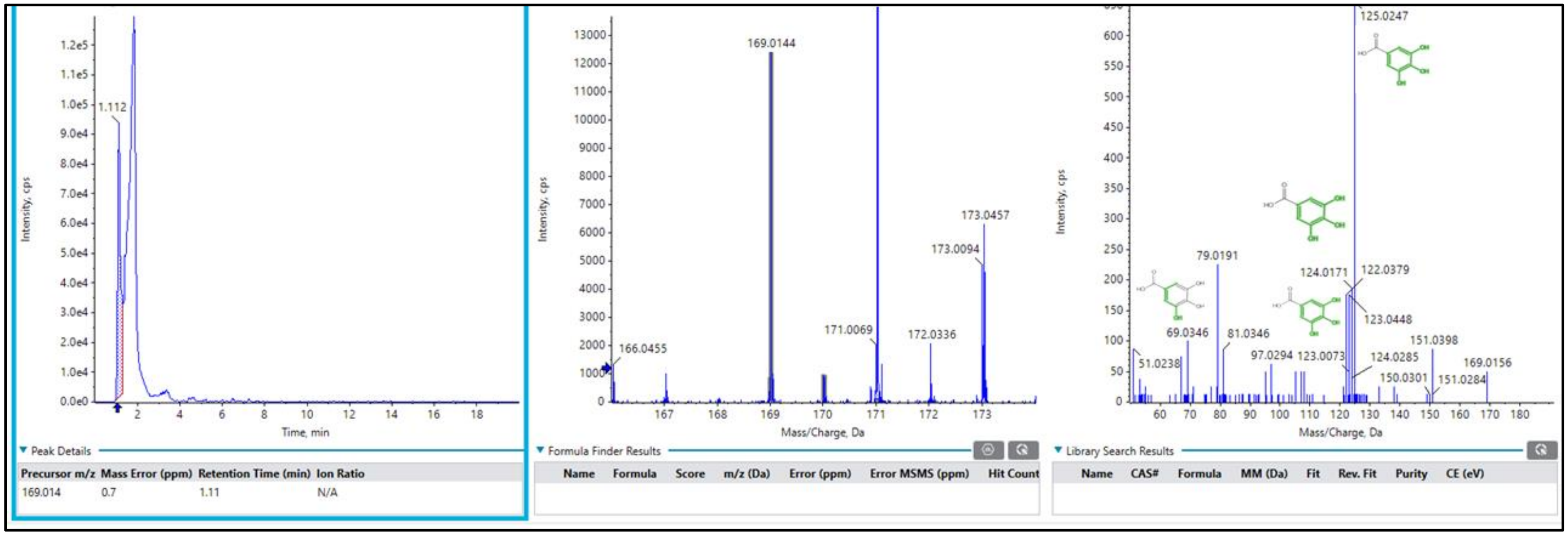


**Figure S21. Extracted ion chromatogram, MS, and MS/MS spectra of gallic acid in OSFL.**

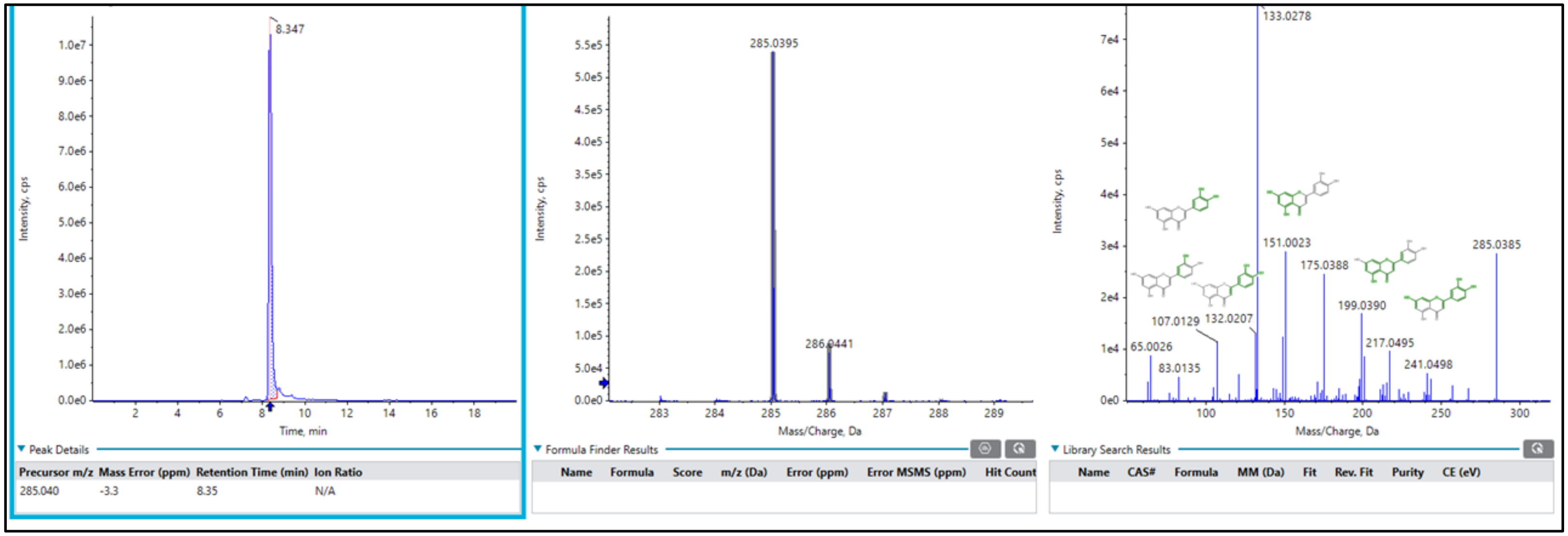


**Figure S22. Extracted ion chromatogram, MS, and MS/MS spectra of luteolin in OSFL.**

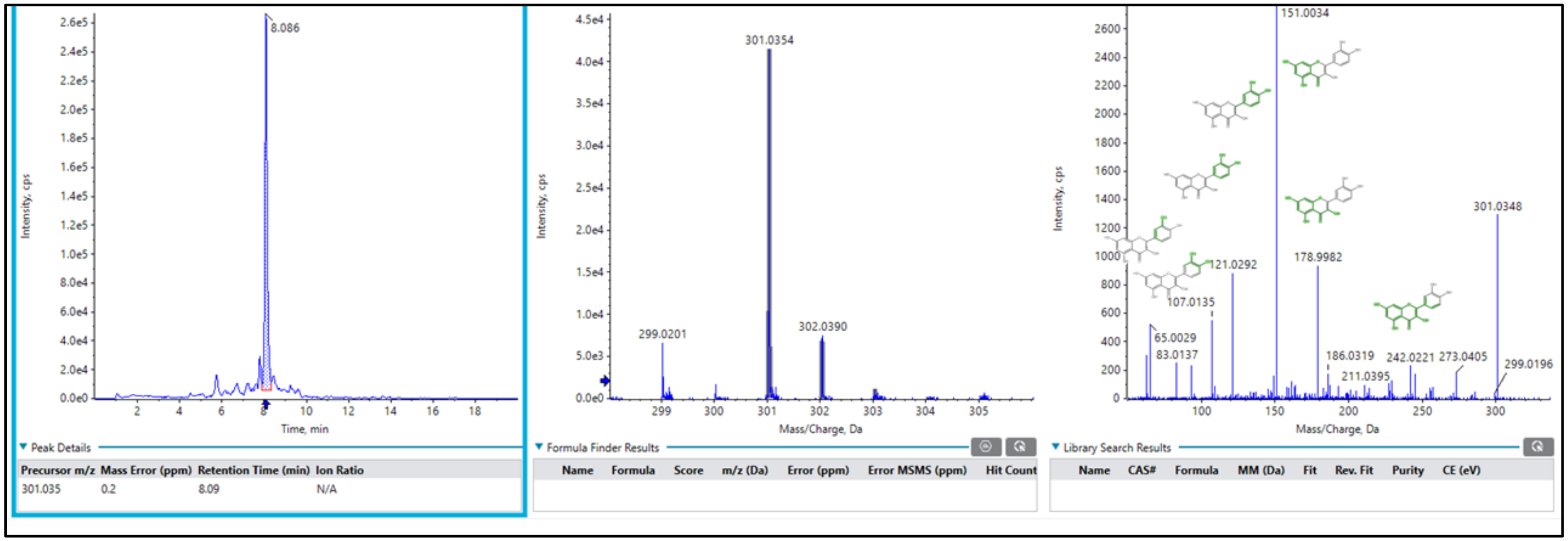


**Figure S23. Extracted ion chromatogram, MS, and MS/MS spectra of quercetin in OSFL.**

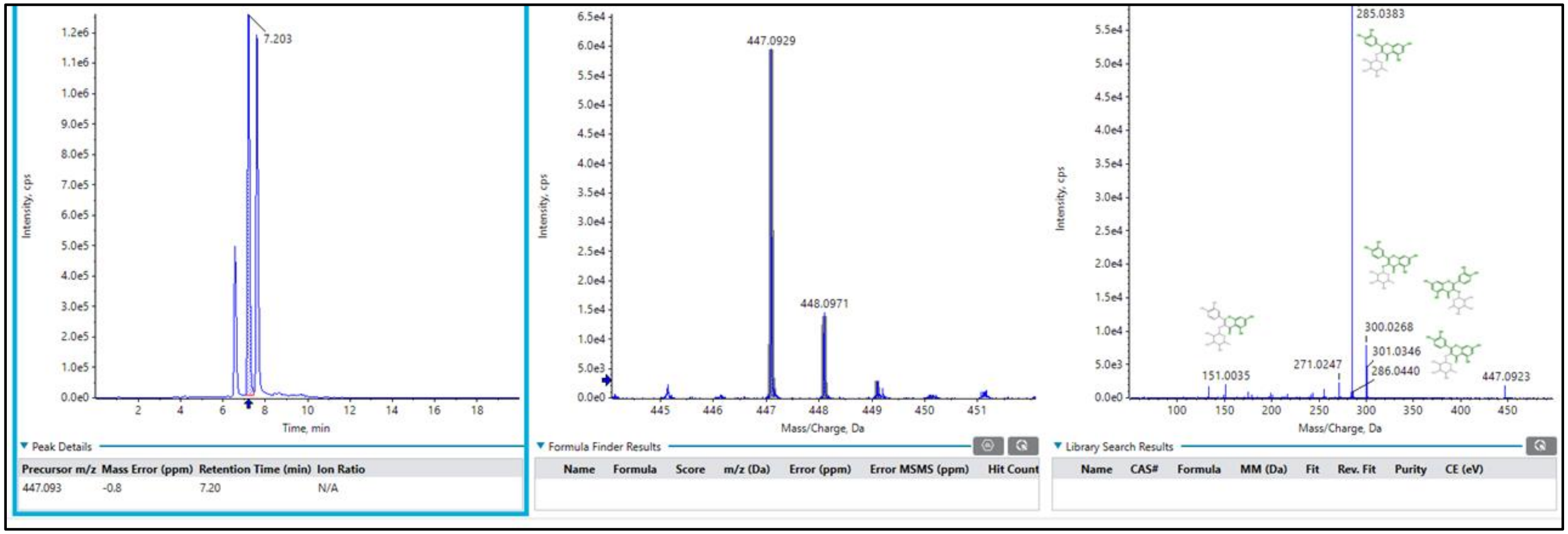


**Figure S24. Extracted ion chromatogram, MS, and MS/MS spectra of quercitrin in OSFL.**

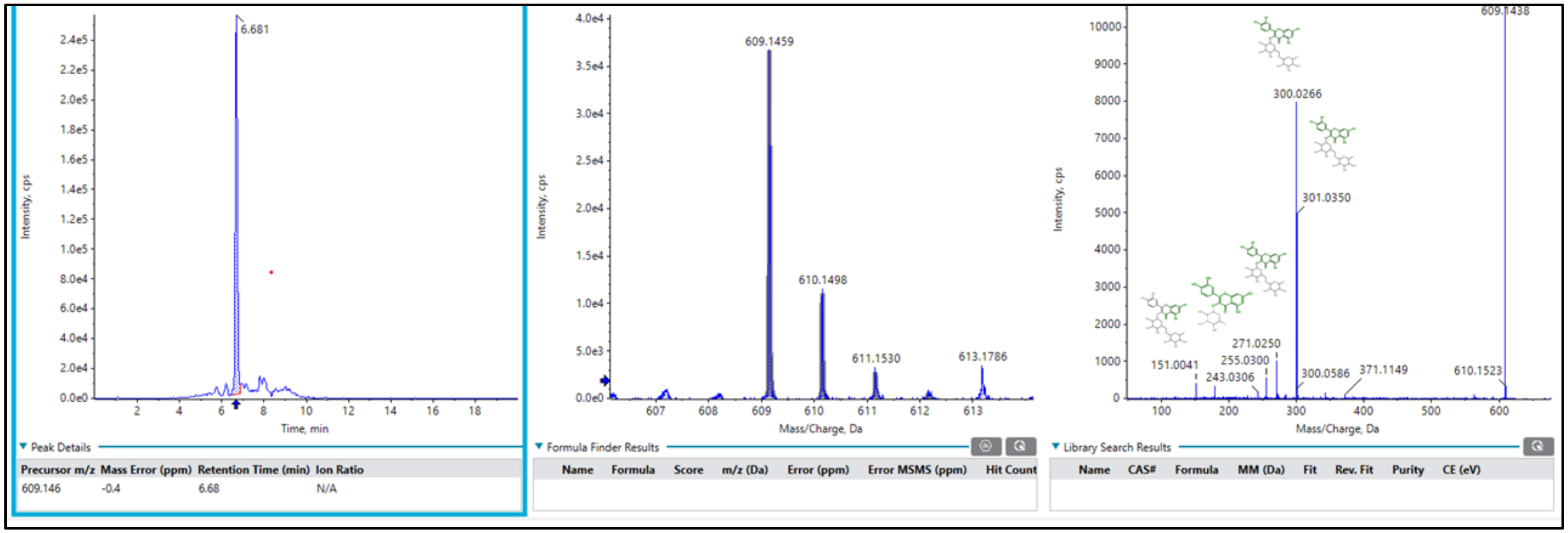


**Figure S25. Extracted ion chromatogram, MS, and MS/MS spectra of rutin in OSFL.**

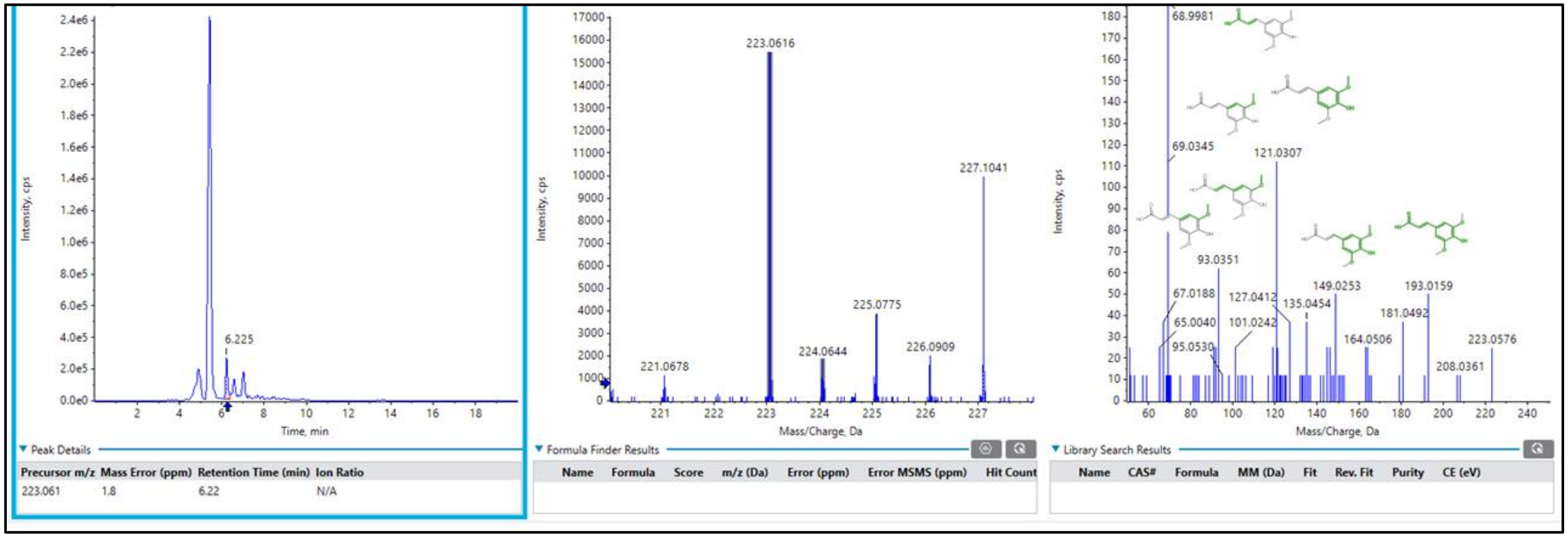


**Figure S26. Extracted ion chromatogram, MS, and MS/MS spectra of sinapic acid in OSFC.**

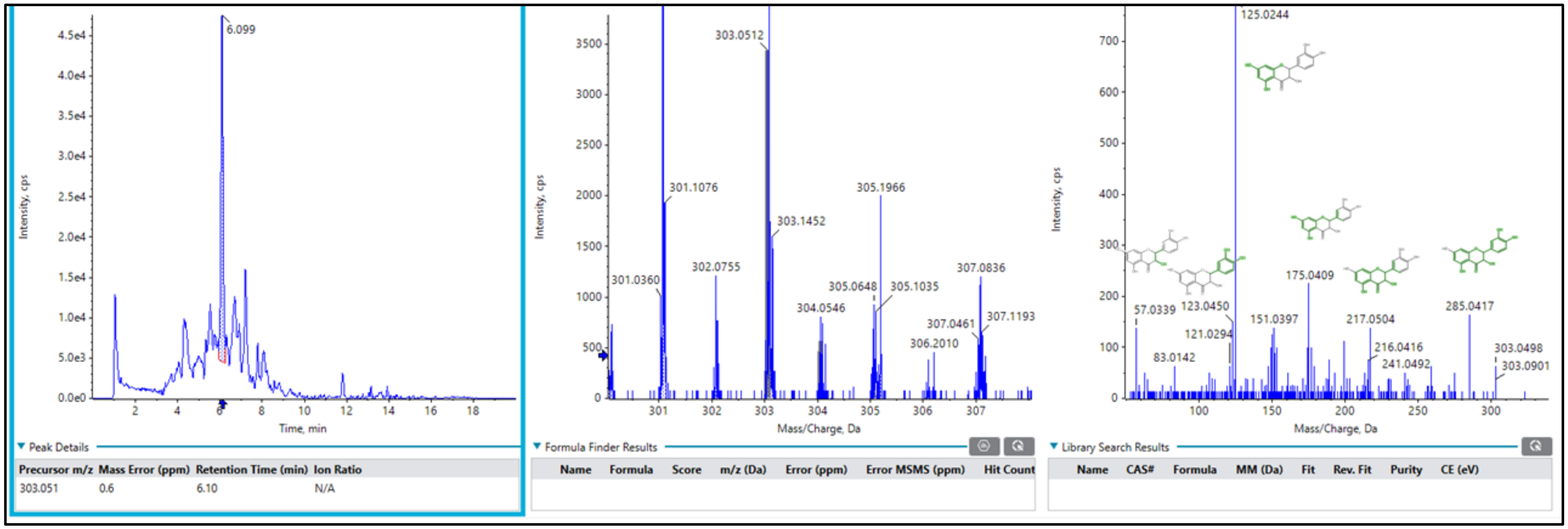


**Figure S27. Extracted ion chromatogram, MS, and MS/MS spectra of taxifolin in OSFL.**

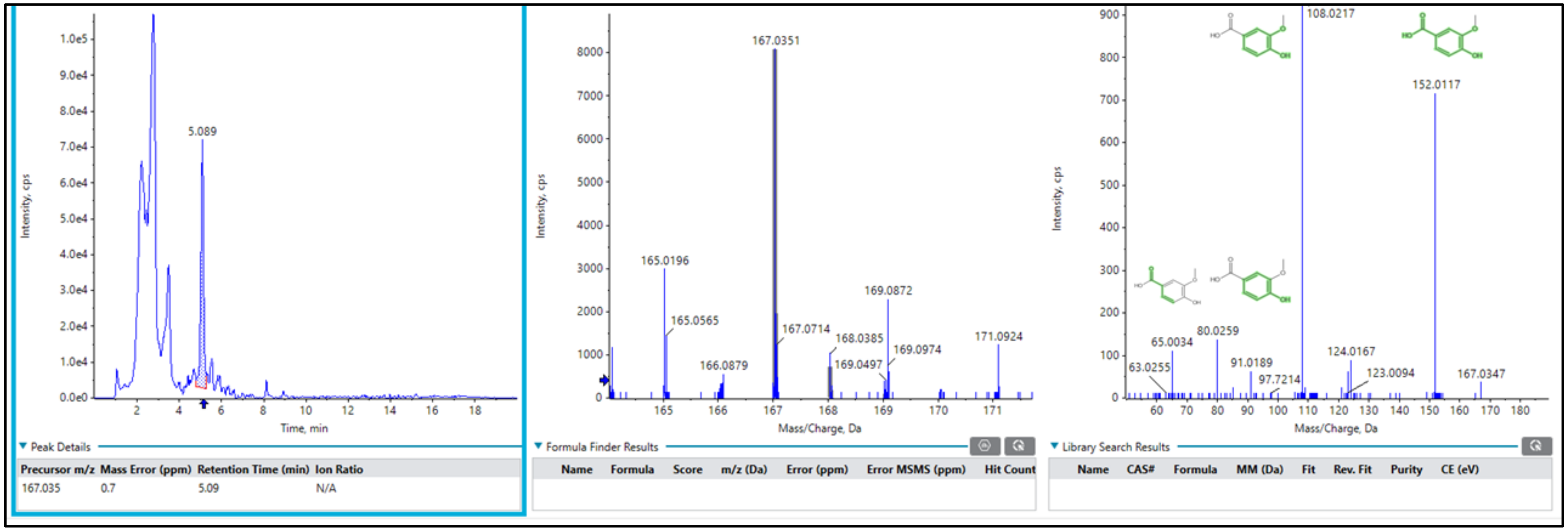


**Figure S28. Extracted ion chromatogram, MS, and MS/MS spectra of vanillic acid in OSFC.**

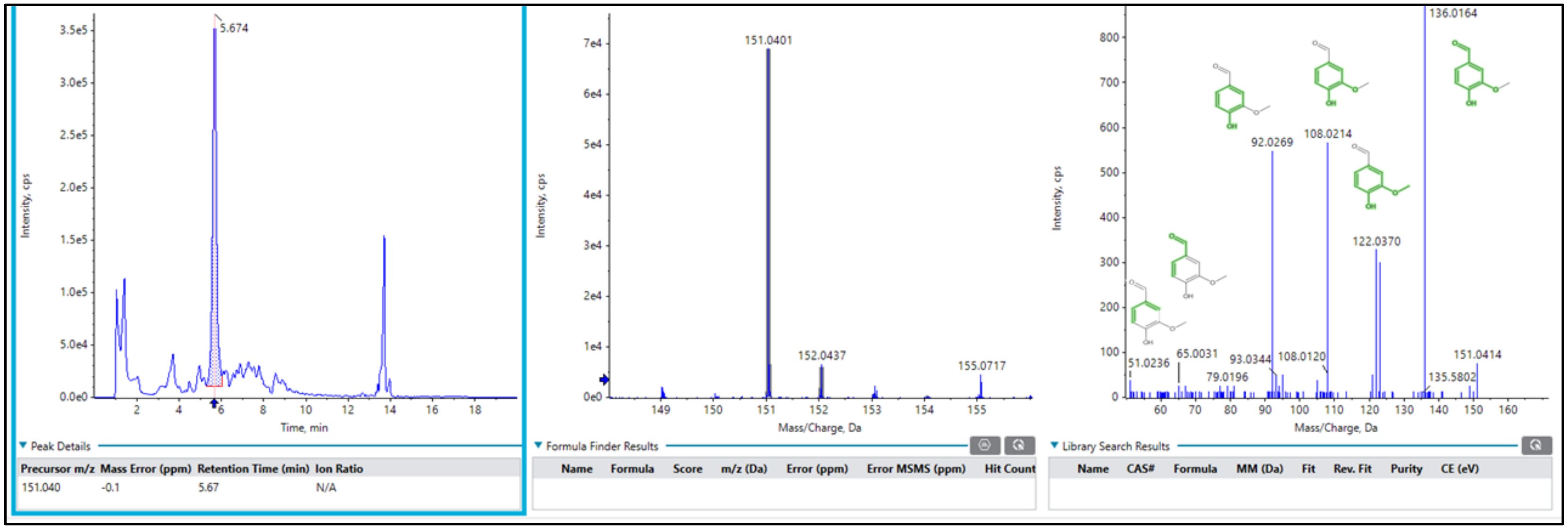


**Figure S29. Extracted ion chromatogram, MS, and MS/MS spectra of vanillin in OSFL.**

**Table S7. Suspect screening list for olive stone flour**

| Compound | Molecular formula | Class |
|---|---|---|
| **1-(3'-methoxy-4'-hydroxy)-phenyl-6,7-dihydroxyisochroman** | $C_{16}H_{16}O_5$ | Hydroxy-isochromans |
| **10-Hydroxy decarboxymethyl oleuropein aglycone** | $C_{17}H_{20}O_7$ | Prenol lipids |
| **10-Hydroxy oleuropein aglycone** | $C_{19}H_{22}O_9$ | Prenol lipids |
| **10-Hydroxy-10-Methyl oleuropein aglycone** | $C_{20}H_{24}O_9$ | Prenol lipids |
| **10-Hydroxydecarboxymethyl oleuropein aglycone** | C17H20O7 | Prenol lipids |
| **1-Acetoxypinoresinol** | C22H24O8 | Furanoid lignans |
| **1-Hydroxypinoresinol** | C20H22O7 | Furanoid lignans |
| **1-phenyl-6,7-dihydroxy-isochroman** | $C_{15}H_{14}O_3$ | Benzopyrans |
| **2,5-Dihydroxybenzoic acid (Gentisic acid)** | $C_7H_6O_4$ | Benzene and substituted derivatives |
| **4-Hydroxybenzoic acid** | $C_7H_6O_3$ | Benzene and substituted derivatives |
| **4-Hydroxyphenylacetic acid** | $C_8H_8O_3$ | Phenols |
| **Abscisic acid** | $C_{15}H_{20}O_4$ | Prenol lipids |
| **Acetoxypinoresinol** | $C_{22}H_{24}O_8$ | Furanoid lignans |
| **Aesculin** | $C_{15}H_{15}O_9$ | Coumarins and derivatives |
| **Apigenin-7-O-glucoside** | $C_{21}H_{20}O_{10}$ | Flavonoids |
| **Azelaic acid** | $C_9H_{16}O_9$ | Fatty Acyls |
| **Berchemol** | $C_{20}H_{24}O_7$ | Lignans |
| **Calceolarioside** | $C_{23}H_{26}O_{11}$ | Cinnamic acids and derivatives |
| **Chrysoeriol (Luteolin 3'-methyl ether)** | $C_{16}H_{12}O_6$ | Flavonoids |
| **Cichoriin** | $C_{15}H_{16}O_{10}$ | Coumarins and derivatives |
| **Decarboxymethyl ligstroside aglycone (Oleocanthal)** | $C_{17}H_{20}O_5$ | Phenols |
| **Decarboxymethyl oleuropein aglycone (Oleacein)** | $C_{17}H_{20}O_6$ | Phenols |
| **Demethyl oleuropein** | $C_{24}H_{30}O_{13}$ | Prenol lipids |
| **Diosmetin** | $C_{16}H_{12}O_6$ | Flavonoids |
| **Elenolic acid** | $C_{11}H_{14}O_6$ | Secoiridoids |
| **Elenolic acid 2-O-glucoside (Oleoside 11-methyl ester)** | $C_{17}H_{24}O_{11}$ | Secoiridoids |
| **Elenolic acid methyl ester** | $C_{12}H_{16}O_6$ | Secoiridoids |
| **Esculetin** | $C_9H_6O_4$ | Coumarins |
| **Fraxamoside** | $C_{25}H_{30}O_{13}$ | Secoiridoids |
| **Fustin** | $C_{15}H_{12}O_6$ | Flavonoids |
| **Gallocatechin** | $C_{15}H_{14}O_7$ | Flavonoids |
| **Hellicoside** | $C_{29}H_{36}O_{17}$ | hydroxycinnamic acid |
| **Homovanillic acid** | $C_9H_{10}O_4$ | Phenols |

| | | |
|---|---|---|
| **Homovanillyl alcohol** | $C_9H_{12}O_3$ | Phenols |
| **Hydroxylated form of elenolic acid** | $C_{11}H_{14}O_7$ | Secoiridoids |
| **Hydroxypinoresinol** | $C_{20}H_{22}O_7$ | Phenols |
| **Hydroxytyrosol** | $C_8H_{10}O_3$ | Phenols |
| **Hydroxytyrosol acetate** | $C_{10}H_{12}O_4$ | Phenols |
| **Hydroxytyrosol glucoside** | $C_{14}H_{20}O_8$ | Phenols |
| **Licodione** | $C_{15}H_{12}O_5$ | Phenol |
| **Ligstroside** | $C_{25}H_{32}O_{12}$ | Prenol lipids |
| **Ligstroside aglycone** | $C_{19}H_{22}O_7$ | Prenol lipids |
| **Ligstroside aglycone dialdehydic form** | C19H22O7 | Prenol lipids |
| **Ligstroside aglycone monoaldehydic form** | C19H22O7 | Prenol lipids |
| **Luteolin-7,4'-O-diglucoside** | $C_{27}H_{30}O_{16}$ | Flavonoids |
| **Luteolin-7-O-glucoside** | $C_{21}H_{20}O_{11}$ | Flavonoids |
| **Maslinic acid** | $C_{30}H_{48}O_4$ | Prenol lipids |
| **Methyl oleuropein aglycone** | C20H24O8 | Prenol lipids |
| **Nuzhenide** | $C_{31}H_{42}O_{17}$ | Secoiridoids |
| **Oleanolic acid** | $C_{30}H_{48}O_3$ | Triterpenic acids |
| **Oleokoronal** | C19H22O7 | Prenol lipids |
| **Oleomissional** | C19H22O8 | Prenol lipids |
| **Oleoside** | $C_{16}H_{22}O_{11}$ | Prenol lipids |
| **Oleuropein** | $C_{25}H_{32}O_{13}$ | Prenol lipids |
| **Oleuropein aglycone** | C19H22O8 | Prenol lipids |
| **Oleuropein aglycone dialdehydic form** | C19H22O8 | Prenol lipids |
| **Oleuropein aglycone monoaldehydic form** | C19H22O8 | Prenol lipids |
| **Olivil** | $C_{20}H_{24}O_7$ | Furanoid lignans |
| **Pinoresinol** | $C_{20}H_{22}O_6$ | Lignans |
| **Quercetin-3-O-glucoside** | $C_{21}H_{20}O_{12}$ | Flavonoids |
| **Quercetin-3-O-rutinoside (Rutin)** | $C_{27}H_{30}O_{16}$ | Flavonoids |
| **Scopoletin** | $C_{10}H_8O_4$ | Coumarins |
| **Secologanoside** | $C_{16}H_{22}O_{11}$ | Secoiridoids |
| **Suspensaside** | $C_{29}H_{36}O_{16}$ | Phenols |
| **Syringaresinol** | $C_{22}H_{26}O_8$ | Lignans |
| **Tyrosol** | $C_8H_{10}O_2$ | Phenols |
| **Tyrosol glucoside (Salidroside)** | $C_{14}H_{20}O_7$ | Phenols |
| **Verbascoside** | $C_{29}H_{36}O_{15}$ | Cinnamic acids and derivatives |
| **Vicenin-2** | $C_{27}H_{30}O_{15}$ | Flavonoids |

**Table S8. Suspect screening results for commercial olive stone flour (OSFC) and olive stone flour from Lemnos (OSFL)**

| Compound | Molecular Formula | [M-H]- experimental OSFC | [M-H]- experimental OSFL | Rt (min) | Fragments m/z | Elemental Formula | OSFC mg/kg | OSFL mg/kg | Mass Bank ID |
|---|---|---|---|---|---|---|---|---|---|
| **10-Hydroxy decarboxymethyl oleuropein aglycone** | $C_{17}H_{20}O_7$ | ND | 335.1145 | 7.14 | 59.0136<br>59.0136<br>95.0496<br>111.0814<br>155.0715<br>199.0612<br>335.1152<br>335.1726 | | **ND** | **13.9** | |
| **10-Hydroxy oleuropein aglycone** | $C_{19}H_{22}O_9$ | 335.2158 | 319.1958 | 8.63 | 123.0442<br>109.0656<br>137.061<br>181.0514 | $[C_7H_8O+H]^-$<br>$[C_7H_8O_2]\text{-}H^-$<br>$[C_8H_8O_2+H]^-$<br>$[C_9H_8O_4+H]^-$ | **12.2** | **3.70** | |
| **10-Hydroxy-10-Methyl oleuropein aglycone** | $C_{20}H_{24}O_9$ | 407.1347 | 407.4589 | 4.25 | 59.0131<br>71.0132<br>101.0237<br>151.0756<br>313.1283<br>357.1183<br>377.1446 | | **4.81** | **4.79** | |
| **1-phenyl-6,7-dihydroxy-isochroman** | $C_{15}H_{14}O_3$ | 241.1256 | ND | 6.34 | 67.0187<br>68.9980<br>69.0345<br>95.0506<br>101.0248<br>121.0297<br>123.0456<br>153.0560 | | **2.63** | **ND** | |
| **2′-Hydroxygenistein 7-O-glucoside** | $C_{21}H_{21}O_{11}$ | 448.1061 | 448.2548 | 6.59 | 65.0026<br>107.013<br>132.0205<br>133.0279 | $[C_4H_3O\text{-}H]\text{-}H^-$<br>$[C_6H_5O_2\text{-}H]\text{-}H^-$<br>$[C_8H_6O_2\text{-}H]\text{-}H^-$<br>$[C_8H_6O_2]\text{-}H^-$ | **7.56** | **8.08** | |
| **2'-hydroxygenistein** | $C_{15}H_{10}O_6$ | 286.0489 | 286.1473 | 8.36 | 65.0032<br>107.0137<br>151.0042<br>175.0406 | $[C_4H_3O\text{-}H]\text{-}H^-$<br>$[C_6H_5O_2\text{-}H]\text{-}H^-$<br>$[C_8H_6O_2\text{-}H]\text{-}H^-$<br>$[C_{10}H_7O_3]^-$ | **0.540** | **14.83** | PN000005 |
| **4-Hydroxybenzoic acid** | $C_7H_6O_3$ | 138.0318 | 138.1256 | 4.27 | 67.0184<br>65.0403<br>92.0268<br>108.0217 | $[C_4H_2O]\text{-}H^-$<br>$[C_5H_4+H]^-$<br>$[C_6H_5O]\text{-}H^-$<br>$[C_6H_5O_2]\text{-}H^-$ | **58.2** | **18.7** | PR100596 |
| **4-Hydroxyphenylacetic acid** | $C_8H_8O_3$ | ND | 152.0147 | 1.38 | 77.04<br>93.0354<br>108.0222<br>121.0298 | $[C_6H_4+H]^-$<br>$[C_6H_5O]^-$<br>$[C_6H_6O_2\text{-}H]\text{-}H^-$<br>$[C_7H_7O_2\text{-}H]\text{-}H^-$ | **ND** | **20.5245** | KO000968 |
| **Apigenin 4′, 7-O-diglucoside** | $C_{27}H_{31}O_{15}$ | 594.1738 | 594.1578 | 6.67 | 95.0501<br>127.04<br>139.0037<br>241.0722 | $[C_6H_6O+H]^-$<br>$[C_6H_6O_3+H]^-$<br>$[C_6H_6O_4\text{-}2H]\text{-}H^-$<br>$[C_{11}H_{12}O_6+H]^-$ | **0.0652** | **0.0417** | |

| | | | | | | | | | |
|---|---|---|---|---|---|---|---|---|---|
| | | | | | 352.1048 | | | | |
| **Apigenin 7-neohesperidoside** | $C_{27}H_{30}O_{14}$ | 577.2541 | 577.1248 | 7.13 | 269.0444<br>268.0379<br>577.1544 | $[C_{15}H_9O_5]$-$H^-$<br>$[C_{15}H_9O_5]^-$ | **0.0405** | **0.3005** | PR305873 |
| **Apigenin-7-O-glucoside** | $C_{21}H_{20}O_{10}$ | 432.1487 | 432.1698 | 6.52 | 59.0139<br>69.0349<br>283.0601<br>311.0573 | $[C_2H_4O_2]$-$H^-$<br>$[C_4H_4O+H]^-$<br>$[C_{16}H_{10}O_5+H]^-$<br>$[C_{17}H_{12}O_6]$-$H^-$ | **0.0143** | **0.0054** | PR309437 |
| **apigenin-7-O-neohesperidoside** | $C_{27}H_{30}O_{14}$ | 577.2658 | 577.2458 | 7.13 | 269.0463<br>268.0386<br>330.1329<br>473.2130 | $[C_{15}H_9O_5]$-$H^-$<br>$[C_{15}H_9O_5]^-$ | **0.0405** | **0.3005** | PR305847 |
| **Apigenin-7-O-β-glucopyranoside** | $C_{21}H_{20}O_{10}$ | ND | 432.1586 | 6.52 | 59.0139<br>69.0349<br>121.0662<br>165.0564<br>283.0601<br>311.0573<br>341.0656<br>431.1389 | | ND | **0.0054** | |
| **benzoic acid** | $C_7H_6O_2$ | 122.0548 | 122.0698 | 5.12 | 65.0396<br>91.0192<br>92.0264<br>93.0344<br>120.0221<br>121.0293 | $[C_5H_5]^-$<br>$[C_6H_4O]$-$H^-$<br>$[C_6H_4O]^-$<br>$[C_6H_4O+H]^-$ | **55.4955** | **22.9413** | KO000319 |
| **Berchemol** | $C_{20}H_{24}O_7$ | 376.1545 | 376.1647 | 4.07 | 59.0134<br>71.0134<br>89.0239<br>101.0242 | $[C_2H_4O_2]$-$H^-$<br>$[C_3H_5O_2$-$H]$-$H^-$<br>$[C_3H_5O_3]^-$<br>$[C_4H_7O_3$-$H]$-$H^-$ | **36.8128** | **21.4659** | |
| **Calceolarioside** | $C_{23}H_{26}O_{11}$ | 478.4256 | ND | 6.5 | 132.0215<br>133.0298<br>153.0562<br>179.0359<br>221.0470 | $[C_8H_7O_2$-$2H]$-$H^-$<br>$[C_8H_7O_2$-$H]$-$H^-$<br>$[C_8H_8O_3+H]^-$<br>$[C_9H_9O_4$-$H]$-$H^-$<br>$[C_{11}H_{12}O_5$-$2H]$-$H^-$ | **21.2616** | **ND** | |
| **Chlorogenic acid** | $C_{16}H_{18}O_9$ | 354.0912 | ND | 4.79 | 85.0297<br>93.0354<br>135.0451<br>191.0281 | $[C_4H_5O_2]^-$<br>$[C_6H_4O+H]^-$<br>$[C_8H_7O_2]^-$<br>$[C_7H_{11}O_6]^-$ | **4.5702** | **ND** | FIO00627 |
| **Chrysoeriol O-glucoside** | $C_{22}H_{23}O_{11}$ | ND | 462.1458 | 9.35 | 59.0137<br>85.0114<br>153.0563 | $[C_2H_4O_2]$-$H^-$<br>$[C_8H_7O_3+2H]^-$ | **0.6081** | **ND** | |
| **Decarboxymethyl ligstroside aglycone (Oleocanthal)** | $C_{17}H_{20}O_5$ | 303.1475 | ND | 6.22 | 124.0241<br>137.0808<br>147.0653<br>183.0662 | $[C_7H_9O_2]$-$H^-$<br>$[C_8H_9O_2]^-$<br>$[C_9H_8O_2]$-$H^-$<br>$[C_9H_{11}O_4]^-$ | **1.4561** | **ND** | |
| **Decarboxymethyl oleuropein aglycone (Oleacein)** | $C_{17}H_{20}O_6$ | 319.1754 | ND | 7.65 | 59.0131<br>97.0658<br>139.0766<br>183.0671 | $[C_2H_2O_2+H]^-$<br>$[C_6H_8O+H]^-$<br>$[C_8H_{11}O_2]^-$<br>$[C_9H_{11}O_4]^-$ | **5.8655** | **ND** | |
| **Elenolic acid** | $C_{11}H_{14}O_6$ | 242.2325 | 242.2579 | 6.31 | 68.9980<br>69.0345<br>95.0506<br>101.0248 | $[C_3H_3O_2$-$H]$-$H^-$<br>$[C_4H_6O]$-$H^-$<br>$[C_6H_9O$-$H]$-$H^-$ | **31.3671** | **6.6338** | |

| | | | | | | | | | |
|---|---|---|---|---|---|---|---|---|---|
| | | | | | 121.0297 | $[C_4H_4O_3+H]^-$<br>$[C_7H_8O_2-2H]-H^-$ | | | |
| **Elenolic acid 2-O-glucoside (Oleoside 11-methyl ester)** | $C_{17}H_{24}O_{11}$ | 404.1358 | 404.148<br>7 | 5.44 | 68.9980<br>95.0506<br>101.0248<br>123.0456<br>153.0560 | $[C_3H_3O_2-H]-H^-$<br>$[C_4H_5O]^-$<br>$[C_6H_7O]^-$<br>$[C_7H_9O_2-H]-H^-$<br>$[C_8H_9O_3]^-$ | **2489.168<br>1** | **149.0134** | |
| **Eriodictyol** | $C_{15}H_{12}O_6$ | 288.0147 | 288.030<br>1 | 5.6 | 153.0019<br>154.0097<br>184.0202<br>199.0440<br>197.0096 | | **7.18** | **2.22** | PR30615<br>2 |
| **Esculetin** | $C_9H_6O_4$ | 178.0412 | 178.035<br>4 | 4.98 | 67.0189<br>81.0345<br>77.0395<br>89.0396<br>121.0302 | $[C_4H_3O]^-$<br>$[C_6H_4+H]^-$<br>$[C_5H_4O+H]^-$<br>$[C_7H_4O+H]^-$<br>$[C_7H_5O_2]^-$ | **74.9** | **10.3** | BML016<br>74 |
| **Fraxamoside** | $C_{25}H_{30}O_{13}$ | 538.5142 | 538.521<br>3 | 7.52 | 59.0136<br>123.045<br>151.0396<br>223.0616 | $[C_2H_3O_2]^-$<br>$[C_7H_6O_2+H]^-$<br>$[C_8H_8O_3]-H^-$<br>$[C_{11}H_{12}O_5]-H^-$ | **9.17** | **21.2** | |
| **Gallocatechin** | $C_{15}H_{14}O_7$ | 306.0785 | 306.045<br>2 | 5.25 | 57.0343<br>109.0294<br>125.0240<br>161.0245<br>195.0297 | $[C_3H_5O]^-$<br>$[C_6H_4O_2+H]^-$<br>$[C_6H_5O_3]^-$<br>$[C_9H_8O_3-2H]-H^-$<br>$[C_9H_8O_5]-H^-$ | **44.0** | **18.0** | BS00390<br>5 |
| **Genistein** | $C_{15}H_{10}O_5$ | 270.0658 | 270.068<br>8 | 9.04 | 65.0027<br>63.0236<br>107.0132<br>117.0335<br>149.0238 | $[C_5H_4]-H^-$<br>$[C_4H_3O-H]-H^-$<br>$[C_6H_4O_2]-H^-$<br>$[C_8H_6O]-H^-$<br>$[C_8H_5O_3]^-$ | **0.021** | **4.27** | PR30551<br>6 |
| **gentistic acid** | $C_7H_6O_4$ | | 238.054<br>8 | 3.26 | 67.0186<br>93.0345<br>95.0499<br>108.0216 | $[C_4H_3O]^-$<br>$[C_6H_6O]-H^-$<br>$[C_6H_6O+H]^-$<br>$[C_6H_6O_2-H]-H^-$ | **7.58** | **288.9** | KO00057<br>5 |
| **Hellicoside** | $C_{29}H_{36}O_{17}$ | 656.1985 | ND | 10.17 | 101.0247<br>223.0620<br>269.1029<br>623.2062 | $[C_4H_5O_3]^-$<br>$[C_{11}H_{13}O_5-H]-H^-$ | **1.95** | **ND** | |
| **Homovanillic acid** | $C_9H_{10}O_4$ | 182.0658 | 182.062<br>2 | 4.88 | 59.0135<br>93.0343<br>109.0293<br>121.0293<br>134.0373 | $[C_2H_3O_2]^-$<br>$[C_6H_4O+H]^-$<br>$[C_6H_4O_2+H]^-$<br>$[C_7H_7O_2-H]-H^-$<br>$[C_8H_6O_2]^-$ | **55.8** | **9.73** | KO00107<br>3 |
| **Homovanillyl alcohol** | $C_9H_{12}O_3$ | 168.0689 | ND | 4.57 | 55.0180<br>67.1888<br>123.0804<br>167.0703 | $[C_3H_3O]^-$<br>$[C_8H_{10}O+H]^-$ | **22.5** | **ND** | |
| **Hydroxypinoresinol** | $C_{20}H_{22}O_7$ | 373.9893 | 373.985<br>2 | 6.88 | 67.0194<br>108.0216<br>136.0164<br>162.0326<br>163.0398 | | **4.31** | **31.0** | |

| | | | | | | | | | |
|---|---|---|---|---|---|---|---|---|---|
| | | | | | 164.0479<br>254.0578<br>269.0824<br>313.1083 | | | | |
| **Hydroxytyrosol** | $C_8H_{10}O_3$ | 154.0789 | 154.0766 | 3.57 | 65.003<br>80.0269<br>81.0342<br>91.0188<br>108.0214 | $[C_4H_3O-H]-H^-$<br>$[C_5H_5O]-H^-$<br>$[C_5H_7O-H]-H^-$<br>$[C_6H_4O]-H^-$<br>$[C_6H_5O_2]-H^-$ | **4.31** | **10.5** | |
| **Hydroxytyrosol acetate** | $C_{10}H_{12}O_4$ | 195.0663 | 195.0675 | 6.43 | 59.0136<br>68.9973<br>77.0389<br>93.0343<br>122.0367<br>149.0244<br>150.032<br>165.0561 | $[C_2H_3O_2]^-$<br>$[C_3H_4O_2-2H]-H^-$<br>$[C_6H_7-H]-H^-$<br>$[C_6H_4O+H]^-$<br>$[C_7H_7O_2]-H^-$<br>$[C_8H_8O_3-2H]-H^-$ | **7.30** | **13.9** | |
| **Hydroxytyrosol glucoside** | $C_{14}H_{20}O_8$ | 316.1158 | 316.1187 | 3.77 | 59.0135<br>135.0442<br>123.0452<br>153.0559 | $[C_2H_4O_2]-H^-$<br>$[C_7H_7O_2]^-$<br>$[C_8H_9O_2-H]-H^-$<br>$[C_8H_9O_3]^-$ | **344.6** | **11.0** | |
| **kaempferol 3-O-glucoside** | $C_{21}H_{20}O_{11}$ | 447.0933 | 447.0930 | 6.59 | 178.9998<br>227.0349<br>284.0324<br>285.0405 | $[C_8H_4O_5]-H^-$<br>$[C_{13}H_9O_4-H]-H^-$<br>$[C_{15}H_9O_6]-H^-$<br>$[C_{15}H_9O_6]^-$ | **0.451** | **0.573** | BS003478 |
| **kaempferol-7-O-neohesperidoside** | $C_{27}H_{30}O_{15}$ | 594.1678 | 594.1655 | 6.67 | 284.033<br>285.0403<br>447.0955 | $[C_{15}H_9O_6]-H^-$<br>$[C_{15}H_9O_6]^-$<br>$[C_{21}H_{19}O_{11}]^-$ | **0.313** | **0.187** | PR305462 |
| **kaempferol-7-O-sopheroside** | $C_{27}H_{30}O_{16}$ | ND | 609.1847 | 6.67 | 125.0246<br>164.0117<br>286.0491<br>301.0707 | $[C_6H_3O_3+2H]^-$<br>$[C_8H_4O_4]^-$<br>$[C_{15}H_9O_6+H]^-$<br>$[C_{16}H_{13}O_6]^-$ | **ND** | **0.256** | |
| **Ligstroside** | $C_{25}H_{32}O_{12}$ | 524.1852 | 524.1876 | 7.98 | 68.9979<br>101.0237<br>127.0397<br>139.0036 | $[C_3H_4O_2-2H]-H^-$<br>$[C_4H_4O_3+H]^-$<br>$[C_6H_7O_3]^-$<br>$[C_6H_6O_4-2H]-H^-$ | **666.3** | **236.6** | |
| **Ligstroside aglycone** | $C_{19}H_{22}O_7$ | 362.4211 | 362.4558 | 7.98 | 68.998<br>101.024<br>111.0086<br>171.0302<br>181.0504 | $[C_3H_4O_2-2H]-H^-$<br>$[C_4H_4O_3+H]^-$<br>$[C_5H_5O_3-H]-H^-$<br>$[C_7H_7O_5]^-$ | **34.3** | **17.4** | |
| **Luteolin-7,4’-O-diglucoside** | $C_{27}H_{30}O_{16}$ | ND | 610.5844 | 6.67 | 178.9987<br>255.0306<br>271.0247<br>300.0267 | $[C_8H_4O_5]-H^-$<br>$[C_{14}H_8O_5]-H^-$<br>$[C_{14}H_8O_6]-H^-$<br>$[C_{15}H_{10}O_7-H]-H^-$ | **ND** | **0.286** | |
| **Luteolin-7-O-glucoside** | $C_{21}H_{20}O_{11}$ | 448.1006 | 448.1014 | 6.59 | 151.0038<br>227.0357<br>284.032<br>285.0394 | $[C_7H_3O_4]^-$<br>$[C_{13}H_8O_4]-H^-$<br>$[C_{15}H_9O_6]-H^-$<br>$[C_{15}H_9O_6]^-$ | **0.503** | **0.640** | PR305606 |
| **luteolin-7-O-neohesperidoside** | $C_{27}H_{30}O_{15}$ | 594.1647 | 594.1655 | 6.67 | 269.0469<br>284.033<br>285.0403<br>447.0955 | $[C_{15}H_9O_5]^-$<br>$[C_{15}H_9O_6]-H^-$<br>$[C_{15}H_9O_6]^-$<br>$[C_{21}H_{19}O_{11}]^-$ | **0.349** | **0.209** | |

| | | | | | | | | | |
|---|---|---|---|---|---|---|---|---|---|
| **Maslinic acid** | $C_{30}H_{48}O_4$ | 472.3511 | 472.3601 | 13.17 | 58.06<br>377.2864<br>393.3153<br>423.325 | $[C_{28}H_{43}O-H]-H^-$<br>$[C_{29}H_{45}O_2-H]-H^-$ | **33.1** | **1233.4** | PR307726 |
| **Naringin** | $C_{27}H_{32}O_{14}$ | 580.0965 | ND | 8.33 | 139.0043<br>121.0297<br>175.0403<br>235.0616<br>295.0835 | $[C_7H_6O_2]-H^-$<br>$[C_{10}H_8O_3]-H^-$<br>$[C_{14}H_{15}O_7]^-$<br>$[C_{16}H_{18}O_8]-H^-$<br>$[C_{16}H_{17}O_9+2H]^-$ | **5.41** | **ND** | |
| **Nuzhenide** | $C_{31}H_{42}O_{17}$ | 686.2125 | 686.2187 | 6.75 | 89.0235<br>101.0235<br>223.0603<br>299.1126<br>421.1487<br>453.1392 | $[C_3H_6O_3]-H^-$<br>$[C_4H_4O_3+H]^-$<br>$[C_{11}H_{12}O_5]-H^-$<br>$[C_{14}H_{19}O_7]^-$<br>$[C_{21}H_{25}O_9]^-$<br>$[C_{21}H_{26}O_{11}]-H^-$ | **1026.3** | **27.9** | |
| **Oleanolic acid** | $C_{30}H_{48}O_3$ | 456.3601 | 456.3689 | 13.59 | 93.0346<br>173.0464<br>191.0563<br>281.2493<br>397.2256<br>456.3590<br>455.3539<br>455.3029 | | **5.53** | **1353.4** | FIO01059 |
| **Oleoside** | $C_{16}H_{22}O_{11}$ | 390.1548 | 390.1478 | 5.27 | 59.0131<br>69.0337<br>89.0238<br>165.0554<br>183.0660 | $[C_2H_3O_2]^-$<br>$[C_4H_5O]^-$<br>$[C_3H_6O_3]-H^-$<br>$[C_9H_{10}O_3]-H^-$<br>$[C_9H_{10}O_4+H]^-$ | **846.9** | **391.0** | |
| **Oleuropein** | $C_{25}H_{32}O_{13}$ | 540.1789 | 540.1720 | 7.37 | 89.0237<br>139.0034<br>149.0239<br>223.0609<br>307.0815<br>327.0884 | $[C_3H_6O_3]-H^-$<br>$[C_6H_6O_4-2H]-H^-$<br>$[C_8H_8O_3-2H]-H^-$<br>$[C_{11}H_{12}O_5]-H^-$<br>$[C_{15}H_{16}O_7]-H^-$<br>$[C_{18}H_{18}O_6-2H]-H^-$ | **954.0** | **183.9** | |
| **Oleuropein aglycone** | $C_{19}H_{22}O_8$ | 378.1589 | ND | 9.25 | 139.0043<br>137.0614<br>153.0558<br>181.0517 | $[C_8H_9O_2]^-$<br>$[C_6H_6O_4-2H]-H^-$<br>$[C_8H_9O_3]^-$<br>$[C_9H_9O_4]^-$ | **8.42** | **ND** | |
| **Olivil** | $C_{20}H_{24}O_7$ | 376.4012 | 376.4089 | 4.07 | 59.0134<br>71.0136<br>101.0239<br>151.0766 | $[C_2H_3O_2]^-$<br>$[C_3H_5O_2-H]-H^-$<br>$[C_4H_7O_3-H]-H^-$<br>$[C_9H_9O_2+2H]^-$ | **36.8** | **21.4** | |
| **Pinoresinol** | $C_{20}H_{22}O_6$ | 358.1399 | 358.1387 | 4.29 | 71.0143<br>133.0663<br>121.0658<br>177.0556<br>357.1195 | $[C_3H_4O_2]-H^-$<br>$[C_8H_7O+2H]^-$<br>$[C_9H_{10}O]-H^-$<br>$[C_{10}H_9O_3]^-$ | **5.91** | **3.51** | PR308002 |
| **quercetin 3-O-galactoside** | $C_{21}H_{20}O_{12}$ | ND | 463.0879 | 6.71 | 151.0037<br>245.0459<br>271.0241 | $[C_7H_4O_4]-H^-$<br>$[C_{13}H_9O_5]^-$<br>$[C_{14}H_9O_6-H]-H^-$ | **ND** | **0.080** | PR309229 |

| | | | | | | | | | |
|---|---|---|---|---|---|---|---|---|---|
| | | | | | 300.0262 | $[C_{15}H_9O_7]$-$H^-$ | | | |
| **quercetin 3-O-rhamnoside** | $C_{21}H_{20}O_{11}$ | 448.1235 | 448.1201 | 6.59 | 151.0038<br>227.0357<br>284.032<br>285.0394 | $[C_7H_4O_4]$-$H^-$<br>$[C_{13}H_8O_4]$-$H^-$<br>$[C_{15}H_9O_6]$-$H^-$<br>$[C_{15}H_9O_6]^-$ | **0.585** | **0.743** | PR305653 |
| **Secologanoside** | $C_{16}H_{22}O_{11}$ | 390.1036 | 390.1874 | 5.27 | 59.0132<br>69.0339<br>89.0239<br>183.0657<br>209.046<br>345.1184 | $[C_2H_3O_2]^-$<br>$[C_4H_5O]^-$<br>$[C_3H_6O_3]$-$H^-$<br>$[C_9H_{10}O_4+H]^-$<br>$[C_{10}H_{11}O_5$-$H]$-$H^-$<br>$[C_{15}H_{21}O_9]^-$ | **846.9** | **391.0** | PS045412 |
| **Suspensaside** | $C_{29}H_{36}O_{16}$ | 640.2087 | 640.2064 | 5.58 | 113.0245<br>151.04<br>161.024<br>179.035<br>459.1519 | $[C_5H_5O_3]^-$<br>$[C_8H_9O_3$-$H]$-$H^-$<br>$[C_9H_7O_3$-$H]$-$H^-$<br>$[C_9H_7O_4]^-$<br>$[C_{20}H_{29}O_{12}$-$H]$-$H^-$ | **104.7** | **18.9** | |
| **Syringaresinol** | $C_{22}H_{26}O_8$ | 417.1554 | 417.1589 | 6.66 | 137.0247<br>166.0274<br>181.051<br>387.1274 | $[C_7H_6O_3]$-$H^-$<br>$[C_8H_7O_4]$-$H^-$<br>$[C_9H_{10}O_4]$-$H^-$<br>$[C_{20}H_{20}O_8]$-$H^-$ | **3.48** | **2.17** | |
| **trans-polydatin** | $C_{20}H_{22}O_8$ | 390.1355 | 390.1389 | 5.3 | 59.0132<br>69.0339<br>183.0657<br>209.046 | $[C_2H_4O_2]$-$H^-$<br>$[C_4H_4O+H]^-$<br>$[C_9H_9O_4+2H]^-$<br>$[C_{10}H_{11}O_5$-$H]$-$H^-$ | **81.6** | **90.5** | PR308317 |
| **Tyrosol** | $C_8H_{10}O_2$ | 138.0789 | 138.0778 | 4.83 | 65.0408<br>93.0342<br>108.0237<br>137.0599 | $[C_5H_5]^-$<br>$[C_6H_5O]^-$ | **20.3** | **103.8** | |
| **Tyrosol glucoside (Salidroside)** | $C_{14}H_{20}O_7$ | 300.1288 | 300.1223 | 4.47 | 59.0133<br>71.0136<br>89.0239<br>85.0294<br>113.0242 | $[C_2H_4O_2]$-$H^-$<br>$[C_3H_5O_2$-$H]$-$H^-$<br>$[C_4H_7O_2$-$H]$-$H^-$<br>$[C_3H_6O_3]$-$H^-$<br>$[C_5H_7O_3$-$H]$-$H^-$ | **444.1** | **5.64** | |
| **Verbascoside** | $C_{29}H_{36}O_{15}$ | 624.2898 | 624.2796 | 6.33 | 133.0296<br>161.0239<br>461.1672 | $[C_8H_7O_2$-$H]$-$H^-$<br>$[C_9H_7O_3$-$H]$-$H^-$<br>$[C_{20}H_{29}O_{12}]^-$ | **1031.2** | **14.2** | |
| **Vicenin-2** | $C_{27}H_{30}O_{15}$ | 593.1509 | 593.1512 | 5.56 | 323.0567<br>353.0658<br>383.0767<br>473.1083<br>503.1198 | $[C_{18}H_{12}O_6]$-$H^-$<br>$[C_{19}H_{14}O_7]$-$H^-$<br>$[C_{20}H_{16}O_8]$-$H^-$<br>$[C_{23}H_{22}O_{11}]$-$H^-$<br>$[C_{24}H_{24}O_{12}]$-$H^-$ | **2.18** | **1.31** | PR309303 |
| **Wighteone** | $C_{20}H_{18}O_5$ | 338.0988 | 338.0987 | 6.05 | 59.0134<br>122.0371<br>123.0452<br>151.04<br>178.865<br>233.0937<br>322.086<br>337.1014 | $[C_7H_5O_2+H]^-$<br>$[C_7H_5O_2+2H]^-$<br>$[C_8H_5O_3+2H]^-$<br>$[C_{19}H_{15}O_5]$-$H^-$ | **0.216** | **0.274** | |

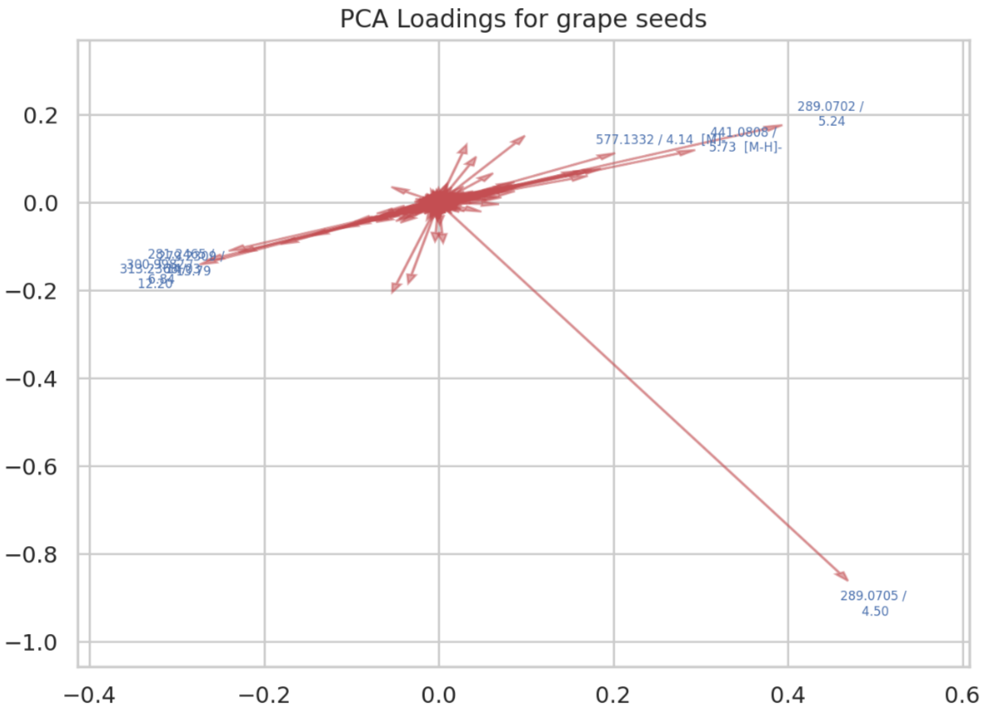


**Figure S30. Loadings plot for GSFC and GSFL corresponding to PCA**

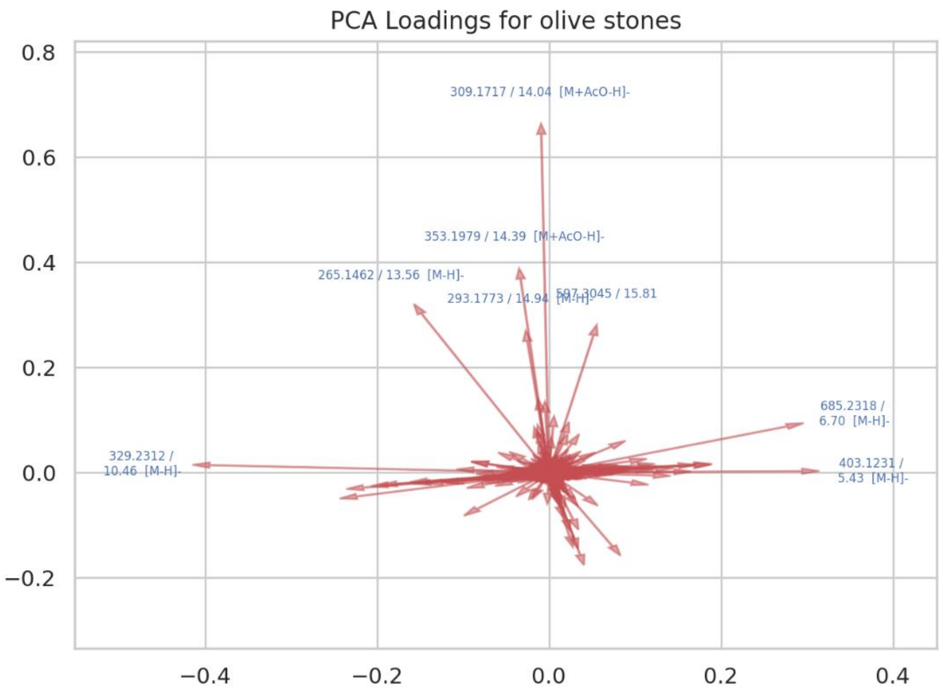


**Figure S31. Loadings plot for OSFC and OSFL corresponding to PCA**

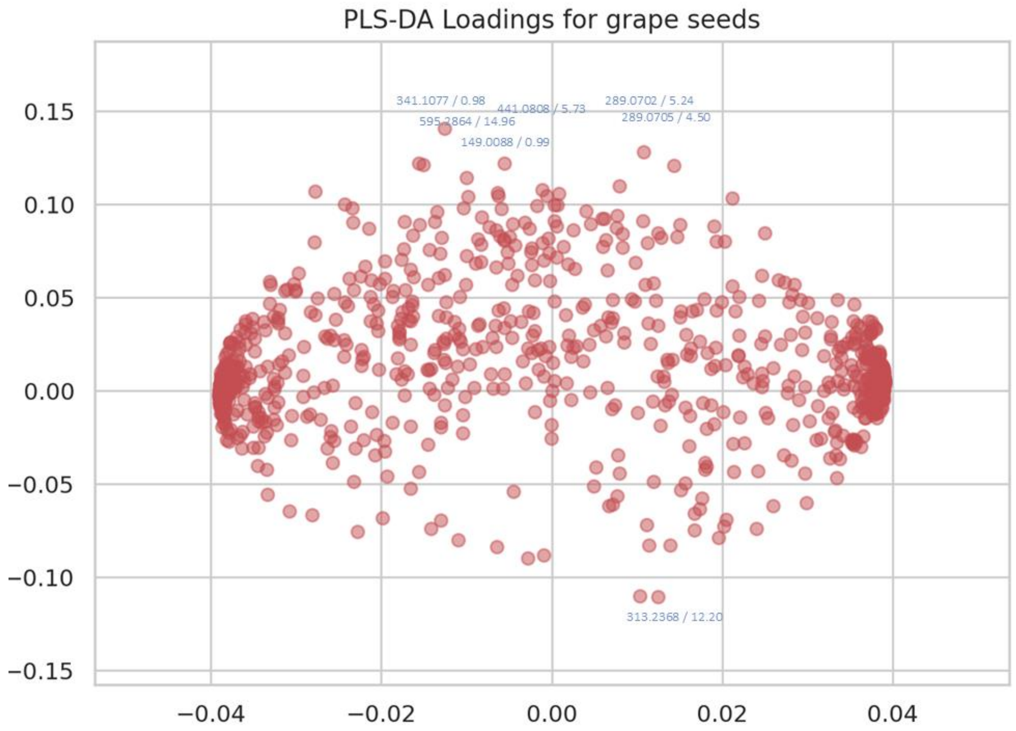


**Figure S32. Loadings plot for GSFC and GSFL corresponding to the PLS-DA model**

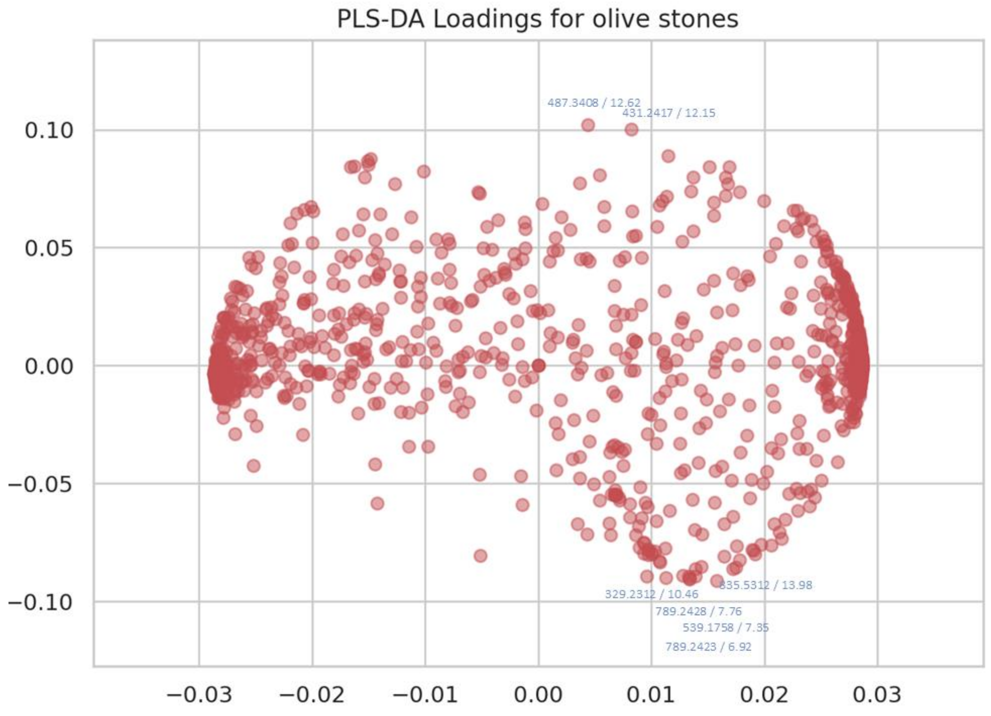


**Figure S33. Loadings plot for OSFC and OSFL corresponding to the PLS-DA model**

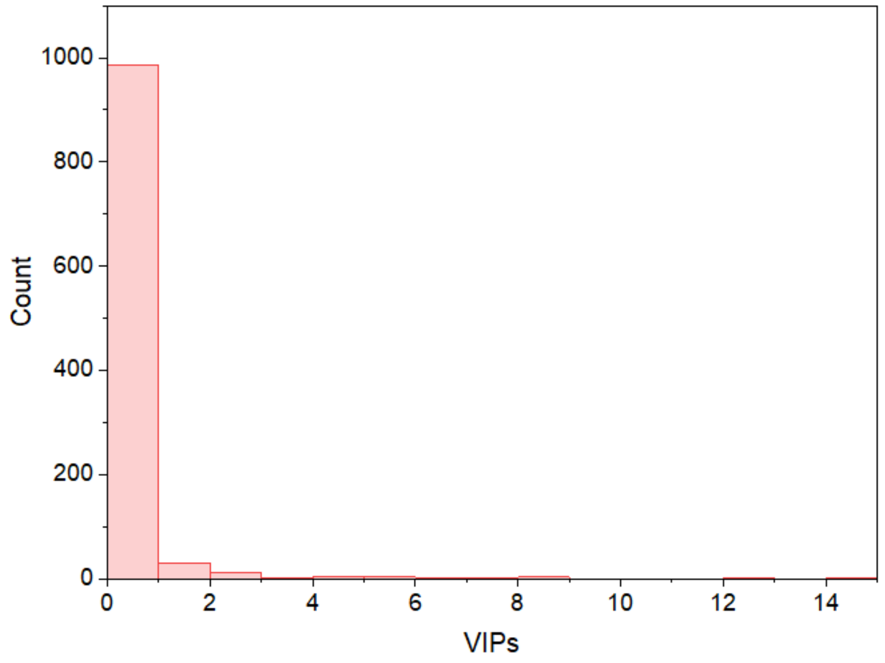


**Figure S34. VIP scores corresponding to the PLS-DA model for GSFC and GSF**

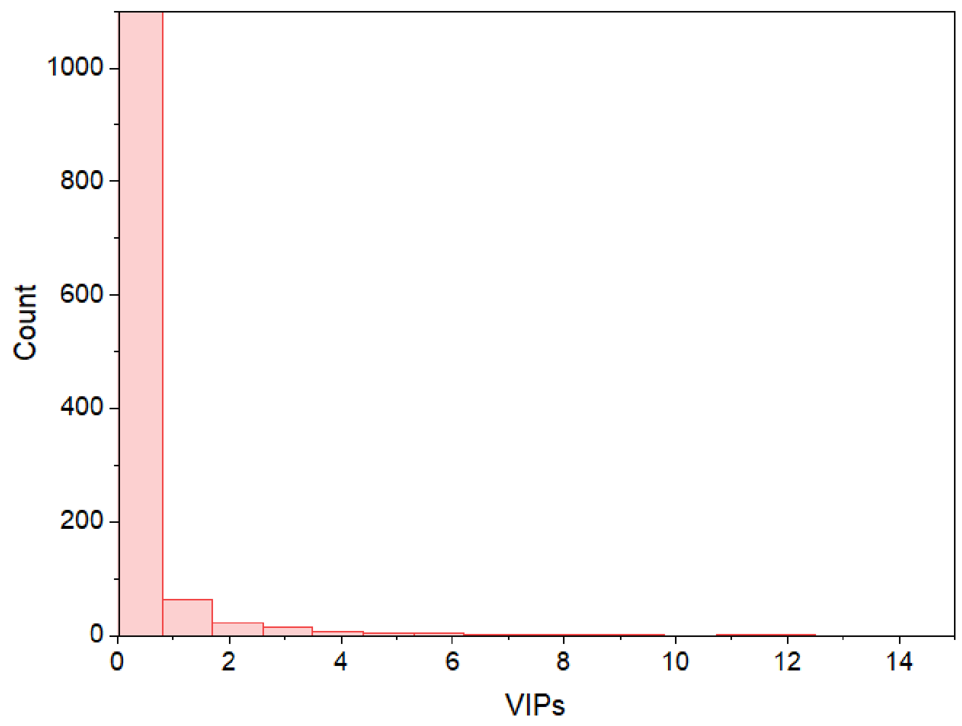


**Figure S35. VIP scores corresponding to the PLS-DA model for OSFC and OSFL**

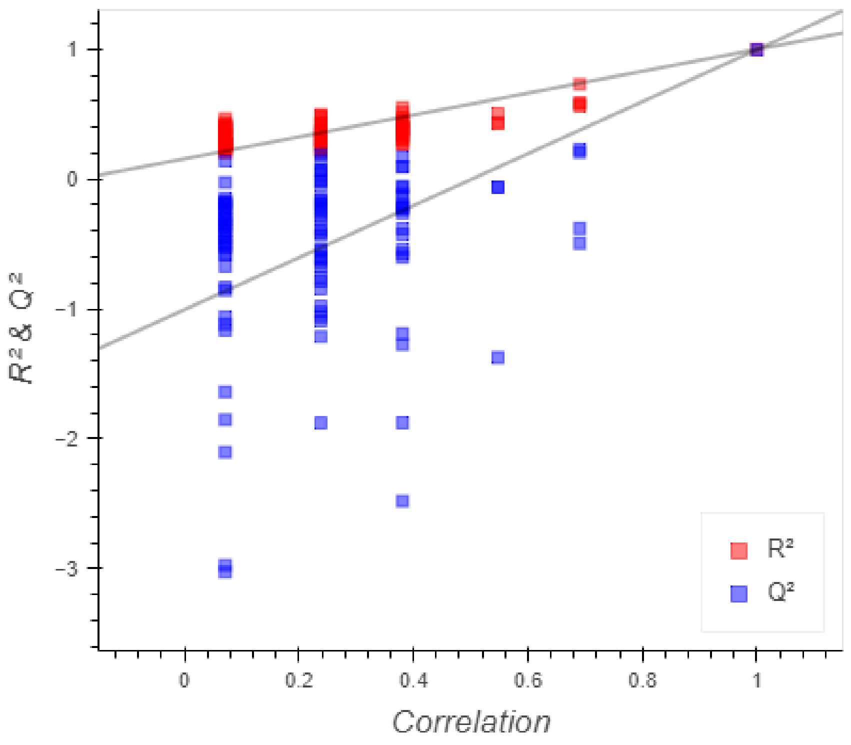


**Figure S36. Permutation Test corresponding to the PLS-DA model for GSFC and GSFL**

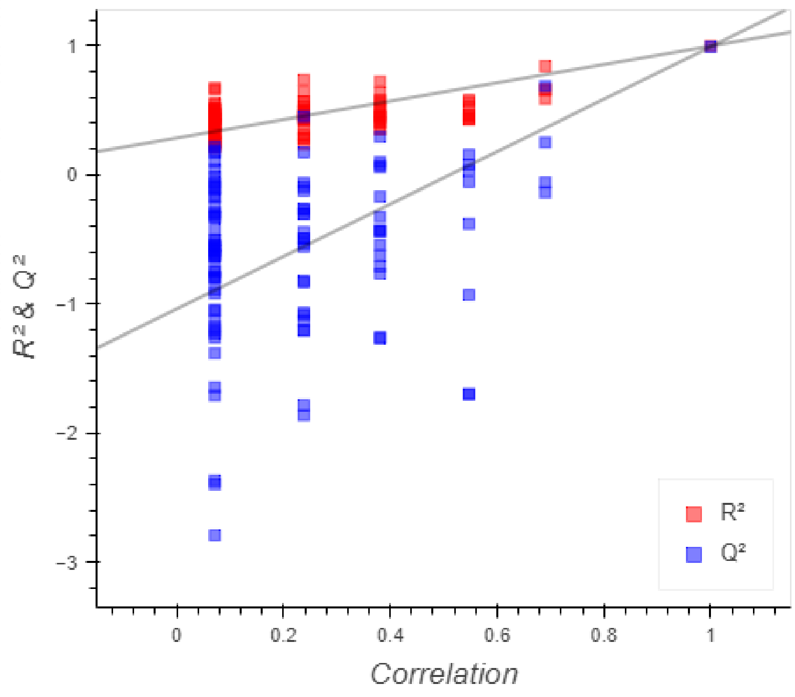


**Figure S37. Permutation Test corresponding to the PLS-DA model for OSFC and OSFL**

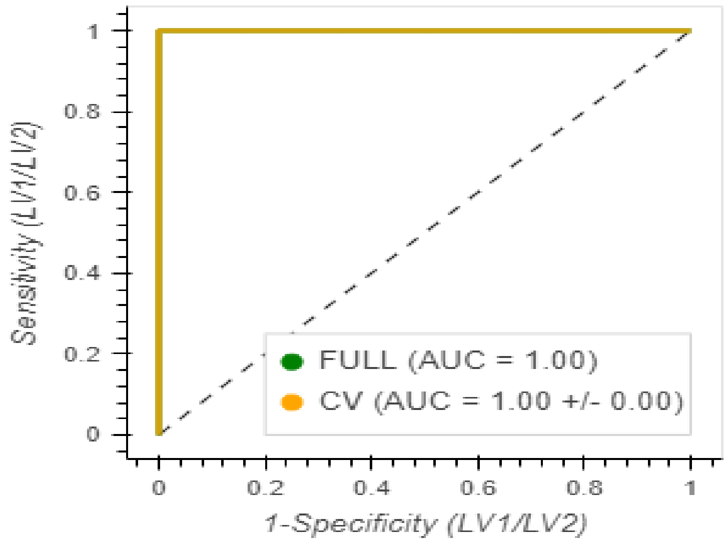


**Figure S38. ROC Curve corresponding to the PLS-DA model for GSFC and GSFL (LV1, LV2: Explanatory variables)**

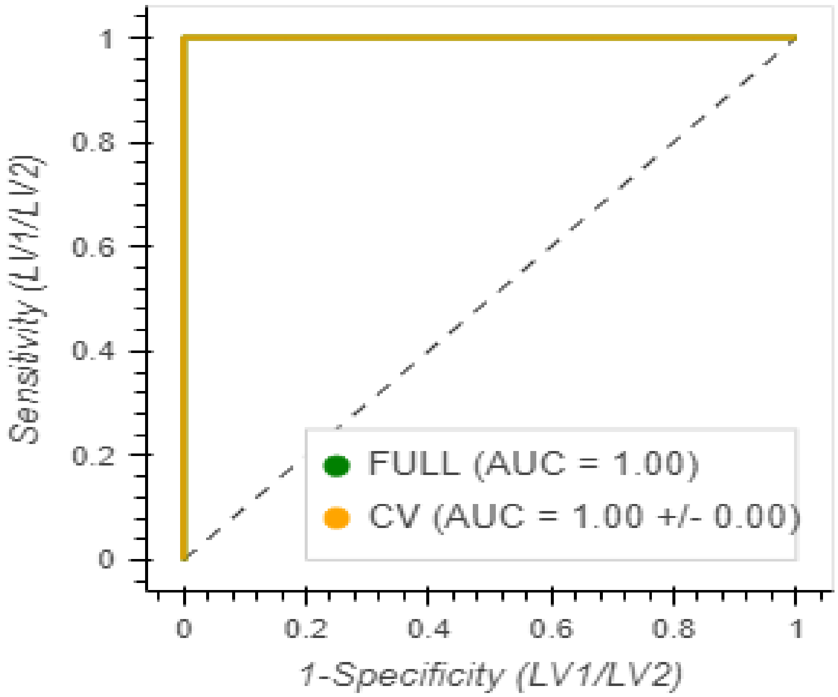


**Figure S39. ROC Curve corresponding to the PLS-DA model for OSFC and OSFL (LV1, LV2: Explanatory variable**
